# Allosteric modulators of M_1_ muscarinic receptors enhance acetylcholine efficacy and decrease locomotor activity and turning behaviors in zebrafish

**DOI:** 10.1038/s41598-024-65445-y

**Published:** 2024-06-28

**Authors:** Corey J. Widman, Sestina Ventresca, Jillian Dietrich, Gwendolynne Elmslie, Hazel Smith, Gina Kaup, Aaron Wesley, Madeline Doenecke, Frederick E. Williams, Isaac T. Schiefer, John Ellis, William S. Messer Jr.

**Affiliations:** 1 Department of Medicinal and Biological Chemistry, College of Pharmacy and Pharmaceutical Sciences, University of Toledo, Mail Stop #1015, 3000 Arlington Ave., Toledo, OH 43614 USA; 2 Department of Pharmacology and Experimental Therapeutics, College of Pharmacy and Pharmaceutical Sciences, University of Toledo, Toledo, OH 43614 USA; 3 Departments of Psychiatry and Pharmacology, College of Medicine, Penn State University, Hershey, PA 17033 USA; 4 Center for Drug Design and Development, College of Pharmacy and Pharmaceutical Sciences, University of Toledo, Toledo, OH 43614 USA

**Keywords:** Medicinal chemistry, Receptor pharmacology, Motor control

## Abstract

Allosteric modulation of muscarinic acetylcholine receptors (mAChR) has been identified as a potential strategy for regulating cholinergic signaling in the treatment of various neurological disorders. Most positive allosteric modulators (PAMs) of mAChR enhance agonist affinity and potency, while very few PAMs (e.g., amiodarone) selectively enhance G protein coupling efficacy. The key structural features of amiodarone responsible for enhancement of mAChR efficacy were examined in CHO cells expressing M_1_ receptors. Subsequent incorporation of these structural features into previously identified allosteric modulators of potency (i.e., n-benzyl isatins) generated ligands that demonstrated similar or better enhancement of mAChR efficacy, lower in vivo toxicity, and higher allosteric binding affinity relative to amiodarone. Notable ligands include **8a**, **c** which respectively demonstrated the strongest binding affinity and the most robust enhancement of mAChR efficacy as calculated from an allosteric operational model. Amiodarone derivatives and hybrid ligands were additionally screened in wildtype zebrafish (*Danio rerio*) to provide preliminary in vivo toxicity data as well as to observe effects on locomotor and turning behaviors relative to other mAChR PAMs. Several compounds, including **8a**, **c**, reduced locomotor activity and increased measures of turning behaviors in zebrafish, suggesting that allosteric modulation of muscarinic receptor efficacy might be useful in the treatment of repetitive behaviors associated with autism spectrum disorder (ASD) and other neuropsychiatric disorders.

## Introduction

The family of G protein-coupled receptors (GPCRs) is one of the most common drug targets in modern drug design^1^; an estimated 35% of approved drugs in the United States and European Union interact with GPCRs^2^. GPCRs are attractive targets due to their accessibility on the extracellular surface of cell membranes and involvement in a wide range of physiological responses in the central nervous system (CNS) and peripheral nervous system (PNS). mAChR are members of the class A GPCR family that respond to acetylcholine (ACh) and are classified into five distinct receptor subtypes (M_1_, M_2_, M_3_, M_4_ and M_5_). The various subtypes are associated with regulating memory and cognitive function (M_1_)^3^, drug reinforcement (M_5_)^4^, locomotor activity (M_1_/M_4_)^5–7^, and cardiovascular/renal/gastrointestinal function (M_2_/M_3_)^8,9^. The five receptor subtypes are characterized by the sequence similarity/identity of highly conserved regions of protein structure, especially amino acids within the seven transmembrane domains and in the membrane-proximal portions of the intracellular and extracellular loops^10,11^. Each mAChR forms a complex with corresponding intracellular G proteins that activate intracellular signaling cascades. M_1_, M_3_ and M_5_ receptors couple to Gα_q/11_ subunits, which stimulate phospholipase Cβ, leading to activation of protein kinase C and calcium mobilization; M_2_ and M_4_ receptors couple to Gα_i/o_ subunits, which inhibit adenylyl cyclase, thereby decreasing cAMP levels^12^. Stimulation of M_1_ muscarinic receptor activity is of potential utility for treating diseases associated with altered cholinergic signaling such as Alzheimer’s disease (AD), Parkinson’s disease (PD), schizophrenia, ASD, dementia, and various other CNS disorders^13–16^.

All mAChR subtypes feature two types of ligand binding, via orthosteric and allosteric sites. The orthosteric site is primarily responsible for receptor activation by the endogenous ligand (ACh) and other orthosteric agonists (e.g., oxotremorine and arecoline). Ligand binding to allosteric sites results in modulation of receptor activity. Early generations of drugs targeting mAChRs included ACh precursors (e.g., choline), acetylcholinesterase inhibitors (e.g., tetrahydroaminoacridine and donepezil), and mAChR agonists (e.g., pilocarpine) all of which directly or indirectly promote interactions with the orthosteric binding site^17^. Many CNS disorders involve altered activity of specific mAChR subtypes, and treatment of these disorders with subtype non-specific agents frequently results in adverse effects^18^. In general, orthosteric ligands all exhibit poor selectivity between the different mAChR subtypes due to the location of orthosteric binding sites in highly conserved regions of the receptor (within the transmembrane domains). The allosteric binding site(s) of the different mAChR subtypes are located in comparatively less highly conserved regions of the receptors (within the extracellular loops); thus, targeting allosteric sites has become an increasingly favored strategy for the development of subtype-selective ligands^14,19^.

There are two classes of models for allosteric regulation of G protein-coupled receptors (including muscarinic receptors): the two-state (and by extension, multistate) models^20^ and operational models^21^. Unless otherwise stated, we will be referring to operational models in the present discussion. These models suggest that it is theoretically possible to modify receptor activity in multiple ways, including (1) enhancing or decreasing agonist affinity, (2) elevating or inhibiting agonist efficacy, and (3) direct activation or inhibition of receptor activity by allosteric ligands. The parameters associated with each of these distinct allosteric modulating effects include: (1) the cooperativity factor α, reflecting changes in agonist affinity; (2) the cooperativity factor β, reflecting changes in agonist efficacy; (3) intrinsic efficacy τ_B_; and (4) the equilibrium dissociation constant K_B_ that reflects the affinity of the ligand for the allosteric binding site^21^. Using these characteristics, allosteric ligands are subsequently categorized as either positive allosteric modulators (PAMs) which enhance potency or efficacy; negative allosteric modulators (NAMs) which diminish potency or efficacy; silent allosteric modulators (SAMs) which bind to the allosteric site but have no effect; and intrinsic allosteric ligands (IALs) which demonstrate allosteric agonist properties^22^. The activity of allosteric ligands that exclusively modulate orthosteric effects are dependent on simultaneous binding of the orthosteric ligand to the receptor. This tertiary complex results in allosteric modulators preserving the endogenous spatiotemporal specificity of the system. Allosteric modulators also exhibit a maximal “ceiling effect” regulated by the concentration of the orthosteric ligand^23^.

Amiodarone is an unusual muscarinic PAM. Simulations by ourselves and others with an allosteric operational model^21^ and with the allosteric two-state model^20,24^ have shown that PAMs that significantly enhance efficacy should also lead to a significant increase in potency of the orthosteric ligand, when other conditions remain the same. However, extensive studies of amiodarone at the M_3_ muscarinic receptor revealed that it did not enhance the potency of acetylcholine, even under conditions that significantly increased the maximal response of acetylcholine. We suggested that this was most likely due to amiodarone’s concomitant negative effect on ACh affinity^24^. A similar phenomenon has been observed at the M_1_ and M_5_ muscarinic subtypes^25,26^. We are not aware of any other PAM of a G protein-coupled receptor that increases the maximal response of the endogenous ligand without enhancing its potency.

The design of allosteric modulators that modify receptor activity appropriately could prove useful in the development of novel therapeutics for CNS disorders with an array of mAChR activity profiles. Positive allosteric modulation of ACh efficacy has potential therapeutic implications for disorders involving low mAChR expression or low mAChR activity. Such is the case with approximately 25% of schizophrenia patients that have 75% fewer M_1_ receptors than healthy patients^27^. Enhancement of M_1_ receptor activity could alleviate cognitive deficits associated with schizophrenia and related disorders. Indeed, in cases where an increase in the maximal effect of neurotransmitter stimulation would be the therapeutic endpoint, efficacy-based PAMs could be selective for just the brain regions that experience a deficit. Regions with sufficient expression or activity to reach the system maximum (E_m_) would not be enhanced^28^, perhaps preventing unwanted effects. On the other hand, psychopharmacological studies show that administration of the muscarinic antagonist scopolamine provides an effective treatment for major depressive disorder^29^. Similar therapeutic outcomes for related cholinergic hypersensitivities may be achieved with greater selectivity by negative allosteric modulation of mAChRs.

Neurochemical abnormalities have implicated the cholinergic system in developmental disorders such as ASD^30^. Recent studies indicate that a selective M_1_ muscarinic agonist alleviates behavioral flexibility deficits and attenuates repetitive behaviors in the BTBR mouse model of ASD^31^, suggesting that selective activation of M_1_ muscarinic receptors could provide an alternative approach for the treatment of ASD patients. M_1_ muscarinic receptor PAMs are of particular interest for the treatment of neuropsychiatric disorders^14,32–34^. Importantly, in systems where ACh responses have failed, PAMs that enhance ACh efficacy could revitalize failed cholinergic signaling^35^, including in neurodevelopmental disorders associated with reduced M_1_ receptor signaling^36^.

In vitro M_1_, M_3_ and M_5_ receptor activity screens typically measure intracellular concentrations of downstream signaling molecules. Intracellular calcium concentrations are transient signal responses as calcium is transported into and out of cellular compartments by several biochemical pathways^37^. In vitro screens that measure intracellular calcium are relatively high-throughput and excellent for demonstrating effects on mAChR potency. Another biochemical signal generated by activation of M_1_, M_3_, and M_5_ receptors is the release of arachidonic acid (AA) from cells. The rate of cellular AA reuptake is nonsignificant over the time course of in vitro screens that measure extracellular AA, thereby enabling the measurement of cumulative mAChR activity, which can clearly demonstrate effects on efficacy modulation^38^.

GPCR signaling cascades are complex biochemical mechanisms that interact with a variety of intracellular enzymes which can complicate their study. By screening GPCR-targeting ligands for activity in vivo using natively expressed receptors followed by a more comprehensive characterization using in vitro assays provides two convergent sets of related data. Comparisons of activity can be enhanced by combining data from multiple distinct screens expressing the same target GPCR (i.e., M_1_ receptors in vitro and M_1_ receptors in vivo)^39^.

**Figure 1:**
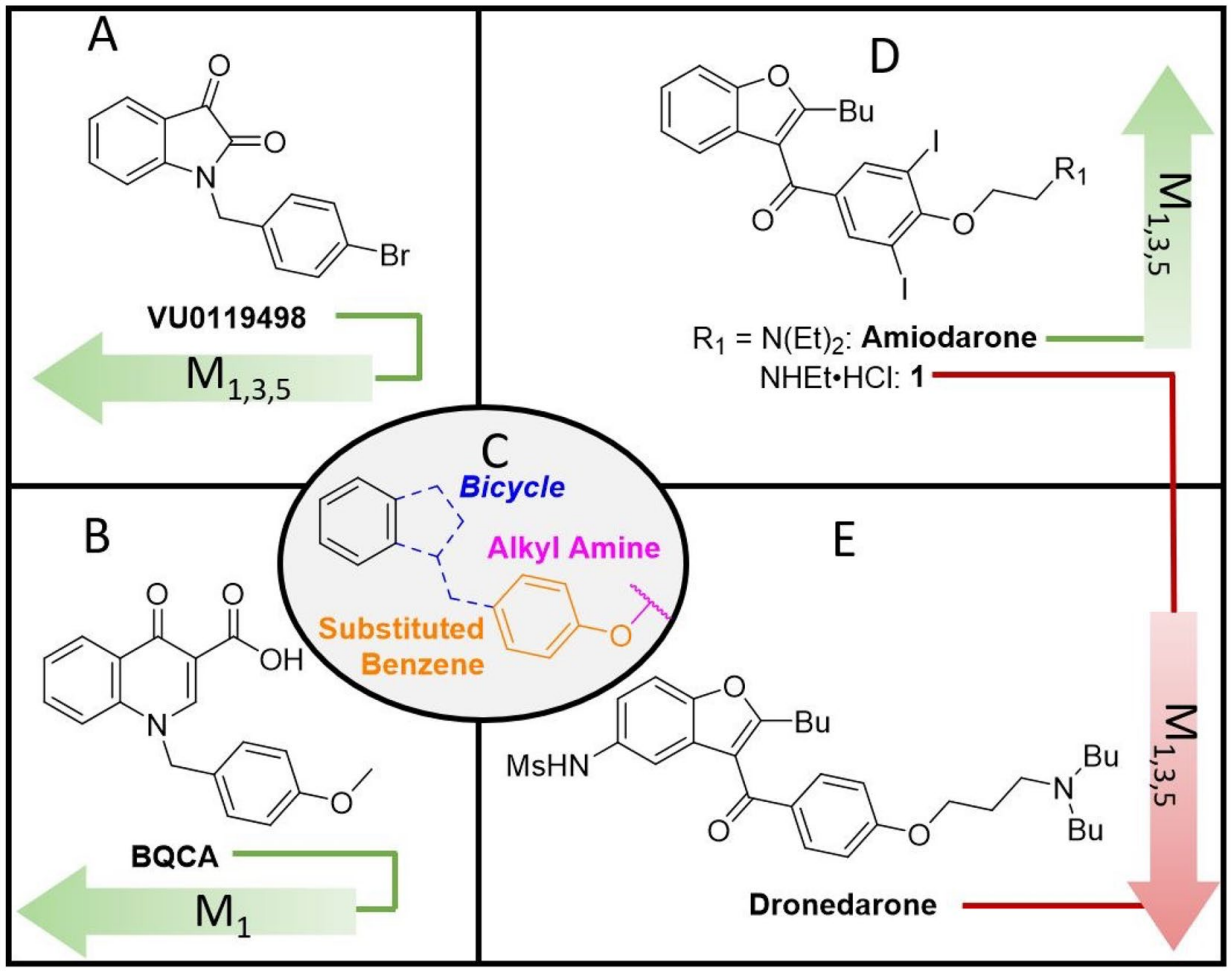
Chemical space overview. (**A**) a non-selective potency PAM (α > 1) containing an isatin ring system (VU0119498); (**B**) an M_1_ selective potency PAM (α > 1) with a methoxy group (BQCA); (**C**) overlapping structural motif highlighting the bicyclic ring system, the substituted benzyl ring and the ether linkage; (**D**) the non-selective efficacy PAM (β > 1) amiodarone and efficacy NAM (β < 1) **1**; (**E**) the non-selective efficacy NAM (β < 1) dronedarone.

In the studies outlined here, the relative importance of functional groups found in amiodarone were explored through the synthesis of several derivatives. Amiodarone is of interest due to its unusual mAChR allosteric profile as detailed above. Although amiodarone has been known to cause pulmonary toxicity with chronic administration^40^, toxicity appears to be generalized to benzofuran containing drugs, including amiodarone and dronedarone^41^. Thus, a focused library of allosteric modulators devoid of benzofurans was designed with a careful consideration of physiochemical properties (i.e., molecular weight, topological surface area, clogP) to enhance CNS activity^42^. Preliminary evaluations of activity were assessed in vitro by measuring [^3^H]-arachidonic acid (AA) release in the absence and presence of 0.1 µM and 100 µM ACh in CHO cells expressing human M_1_ muscarinic receptors. Compounds exhibiting activity in preliminary assays were evaluated further in vitro using full ACh dose response curves. Compounds also were tested in vivo to assess preliminary toxicology and effects on locomotor behaviors in zebrafish (*Danio rerio*). Based on the structures of BQCA/VU0119498 (potency modulators) and amiodarone/dronedarone (efficacy modulators)^25^, we hypothesized that compounds combining key structural features could result in useful additive effects (i.e., potency and efficacy modulation, Fig. 1). Such compounds could bind to multiple distinct binding sites as has been found previously with bitopic ligands composed of multiple pharmacophores attached via a linker^43^. The ligands discussed herein alternatively incorporate moieties or features of various structurally related ligands that interact with distinct binding sites. Specifically, the isatin ring system in VU0119498, the methoxy moiety in BQCA, and the di-iodo-phenyl system in amiodarone were identified as key molecular features to explore in various combinations in the development of allosteric modulators of M_1_ receptor efficacy.

## Results

**Figure 2:**
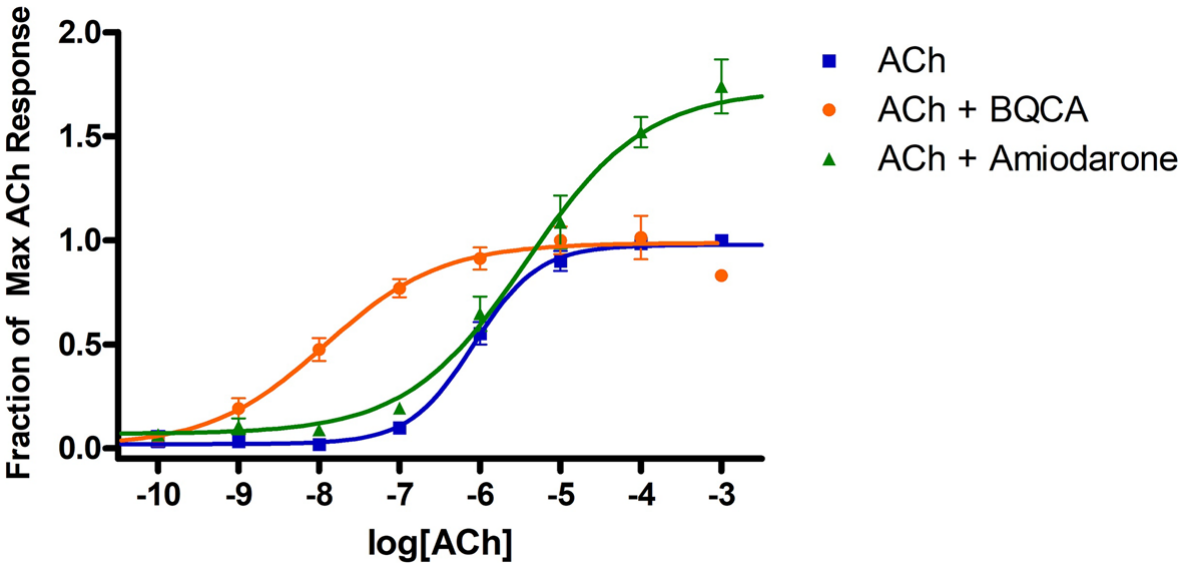
Modulation of ACh response by BQCA and amiodarone at M_1_ receptors. Release of [^3^H]AA by the indicated concentrations of ACh from CHO cells expressing M_1_ muscarinic receptors was measured in the presence and absence of 10 µM BQCA or 30 µM amiodarone. The points are the mean ± SEM from at least four determinations. Curves represent best fits to the four-parameter model described in Methods. To minimize the agonist effect of BQCA, these assays included pretreatment of the cells with 0.3 µM phenoxybenzamine (POB) as described in Methods. The treatment with POB reduced receptor number by 90%.

**Figure 3:**
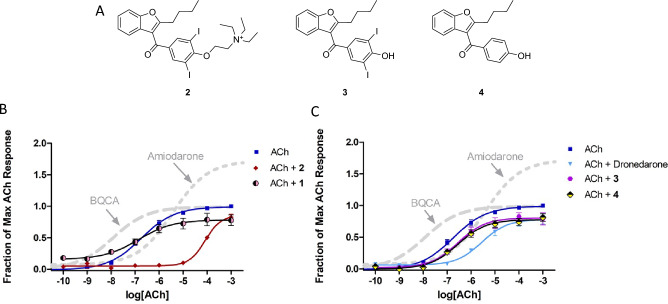
Modulation of ACh response by allosteric modulators at M_1_ receptors. Assays were conducted as in Fig. 2, except that the cells were not pretreated with phenoxybenzamine. (**A**) Structures of compounds included in the figure; (**B**, **C**) Data from Fig. 2 for BQCA (long dashes) and amiodarone (short dashes) are included for reference; the other allosteric modulators were all tested at 10 µM. Data points are the mean ± SEM of at least four determinations. Curves represent the best fits to the four-parameter model described in Methods.

Amiodarone is best known as an antiarrhythmic agent that blocks potassium channels, but it has also been shown to enhance mAChR activity through an allosteric mechanism^24–26^. Initial studies confirmed that amiodarone is a PAM of ACh at M_1_ receptors, based on measurements of [^3^H]-AA release in stably transfected CHO cells (Fig. 2; Table 1). Note that amiodarone enhanced the maximal response of ACh without any appreciable effect on potency. In contrast, under the same conditions, BQCA enhanced potency without appreciable effect on maximal response, as reported by many others. Compounds that are structurally related to amiodarone were also evaluated, including dronedarone, des-ethyl amiodarone (**1**), N-ethyl amiodarone (**2**), a related phenol (**3**), and an analogous phenol lacking the bis-halide substitution (**4**) (Fig. 3A; Table 2). Interestingly, 2 was a NAM of mAChR potency and efficacy (Fig. 3B) in a similar fashion as previously reported for dronedarone^25^ (Fig. 3C). In contrast, compound **1** and the phenols (**3** and **4**) suppressed the maximal ACh response (Fig. 3B,C).

**Table 1:** Stimulation of ^3^[H]AA release by allosteric ligands in the presence and absence of ACh at M_1_ receptors.

Compound	Concentration ( µM)	No ACh	0.1 µM ACh	100 µM ACh
BQCA	10	0.02 ± 0.00	1.80 ± 0.10	1.03 ± 0.09
Amiodarone	30	0.10 ± 0.04	0.45 ± 0.07	1.54 ± 0.06
Dronedarone	10	0.08 ± 0.01	0.25 ± 0.05	0.75 ± 0.07
VU0119498	10	0.06 ± 0.01	1.28 ± 0.12	1.03 ± 0.05

The agonist effect of the allosteric ligand by itself (“No ACh”) is expressed as the fraction of the maximal response that could be elicited by ACh. The ability of each allosteric ligand to modulate the response of 0.1 or 100 µM ACh is expressed as the response to that concentration of ACh with the modulator present divided by the response to that concentration of ACh alone. The response to ACh alone was taken as the average of all assays in this study; for 0.1 µM, that average was 0.428, for 100 µM, it was 0.980. The entries for BQCA, amiodarone, and dronedarone can be compared with the curves shown in Fig. 3C. BQCA is a potency-based PAM–it enhances the response to the low concentration of ACh by 80% but does not have a significant effect on the high concentration of ACh. Amiodarone is an efficacy-based PAM–it enhances the maximal effect of ACh, while actually reducing the response at the low concentration. Dronedarone is a NAM that reduces the response at both concentrations of ACh. Data represent the means ± s.e.m. for at least 4 determinations.

**Table 2:** Stimulation of ^3^[H]AA release by ACh in the presence and absence of potential benzofuran allosteric modulators of M_1_ receptors expressed in CHO cells. Data are presented as in Table 1, as means ± s.e.m. for 4 or more determinations.

		Assay conditions		
Compound	Structure	No ACh	0.1 µM ACh	100 µM ACh
**1**		0.18 ± 0.03	1.01 ± 0.11	0.83 ± 0.09
**2**		0.04 ± 0.02	0.13 ± 0.04	0.50 ± 0.03
**3**		0.04 ± 0.01	0.63 ± 0.10	0.90 ± 0.07
**4**		0.04 ± 0.01	0.62 ± 0.07	0.81 ± 0.06
**5**		0.22 ± 0.03	1.86 ± 0.42	1.47 ± 0.09
**6a**		0.05 ± 0.01	1.37 ± 0.07	1.06 ± 0.04
**6b**		0.08 ± 0.02	1.78 ± 0.12	1.36 ± 0.07
**6c**		0.04 ± 0.01	0.40 ± 0.11	1.04 ± 0.05

**Figure 4:**
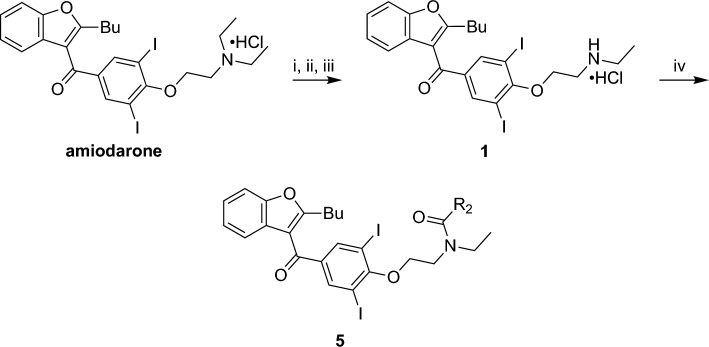
Synthesis of benzofuran amide derivatives. Reagents and conditions: (i) ACE-Cl [5.0 eq], 1,2-DCE, 0 °C, 1 h; (ii) 1,2-DCE, 80 °C, 2 h; (iii) MeOH, 80 °C, 1 h, 16%; (iv) acetic anhydride [2.0 eq], Et_3_N [2.0 eq], DMAP [cat], DCM, 0 °C to RT, 16 h, 14–41%.

**Figure 5:**
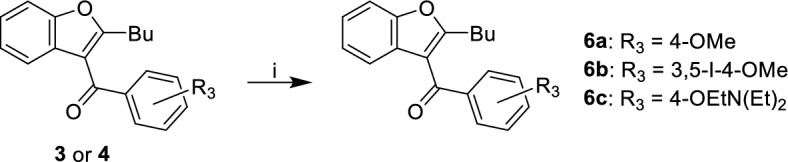
Synthesis of benzyl-substituted benzofurans. Reagents and conditions: (i) alkyl-halide [1.5 eq], Cs_2_CO_3_ [2.0 eq], NaI [cat], DMF, 60 °C, 2 h, 40–50%.

The most compelling aspect of the initial amiodarone SAR studies was that modification of the electronics of the amine resulted in both positive allosteric effects (with amiodarone) and negative allosteric effects (with **1**, **2** and dronedarone). With this in mind, we modified the amine via acetylation to afford **5**. These analogues were synthesized through two synthetic routes. The first route (Fig. 4) started with the n-dealkylation reaction of amiodarone with 1-chloroethyl chloroformate in refluxing 1,2-dichloroethane to afford the corresponding carbamate intermediate, followed by a decomposition and reaction with the corresponding anhydride to yield **5**. Additionally, a second route (Fig. 5) employed the commercially available 2-butyl-3-benzoyl-benzofurans **3** and **4**, which were reacted with cesium carbonate and various alkyl-halides in a Williamson ether synthesis to produce the corresponding ethers (**6a–c**) to confirm the SAR observations seen with **3**, **4**, and amiodarone.

**Figure 6:**
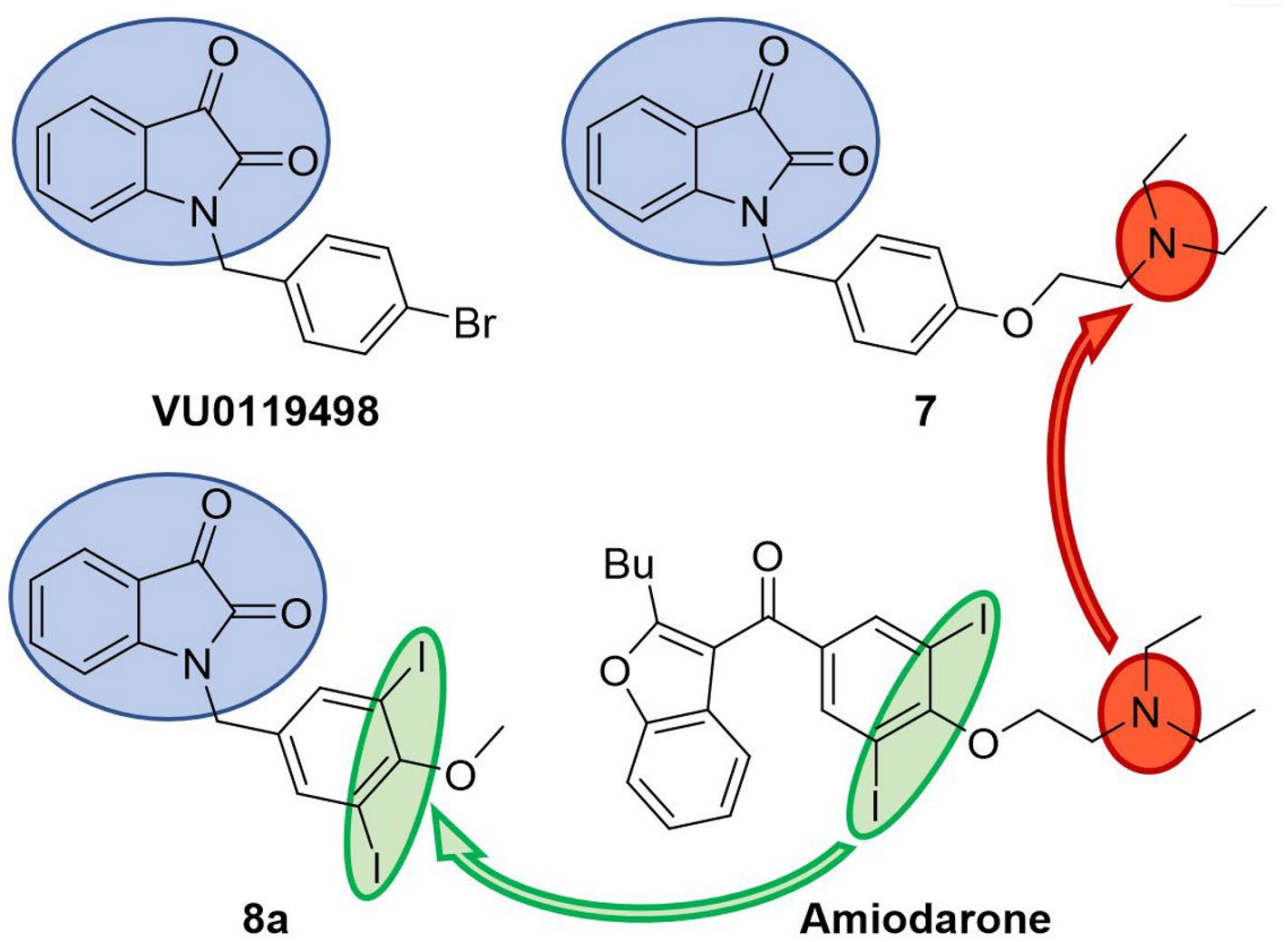
Design of novel allosteric modulators. Depiction of combinations with the isatin heteroaromatic core from VU0119498 and structural features of interest from amiodarone.

The design of novel ligands was inspired by a perceivable overlap of the structure activity relationships (SAR) that have been reported by others for select allosteric modulators^44,45^. For instance, the mAChR allosteric modulators VU0119498^46^ and BQCA^32^ contain similar structural components; a bicyclic heterocycle bridged to a para-ether substituted benzene ring. However, despite having similar structural motifs, these compounds exhibit an array of allosteric effects on mAChR and differ in receptor subtype specificities (or lack thereof). Furthermore, the ether linkage may be extended to feature an alkyl amine, while substitutions may be added to the benzene ring, such as iodides as seen in amiodarone. Such changes, which in addition to influencing allosteric modulation of potency, may also invert modulatory activity from positive to negative (or vice versa). Hence, we chose to examine if the drastic activity cliffs observed in the amiodarone SAR described above applied across other chemical classes of allosteric modulators. Emphasis was placed on combining amiodarone structural features with isatin, incorporating this heteroaromatic core in the design of potent and selective muscarinic NAMs and PAMs, **7** and **8a**, respectively (Fig. 6).

**Figure 7:**
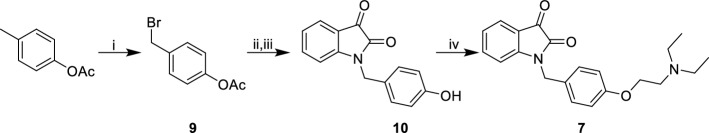
Synthesis of isatin hybrids **7** and **10**. Reagents and conditions: (i) NBS [1.3 eq], AIBN [cat], CCl_4_, 76 °C, 2 h, 86%; (ii) isatin [0.77 eq], K_2_CO_3_ [1.54 eq], KI [0.77 eq], DMF, RT, 4 h ; (iii) K_2_CO_3_ [2.0 eq], MeOH, RT, 2 h, 25% ; (iv) ClEtN(Et_2_)HCl [1.5 eq], Cs_2_CO_3_ [2.0 eq], NaI [cat], DMF, 60 °C, 2 h, 20%.

**Figure 8:**
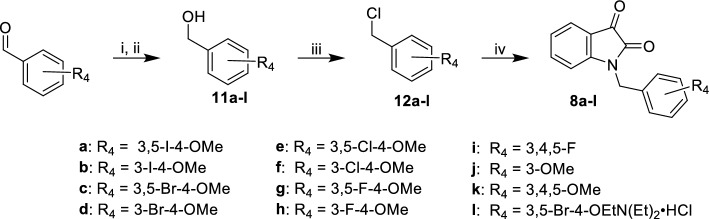
Synthesis of isatin hybrids **8a-l** Reagents and conditions: (i) alkyl-halide [1.5 eq], Cs_2_CO_3_ [2.0 eq], NaI [cat], DMF, 60 °C, 2 h; (ii) NaBH_4_ [1.5 eq], MeOH, RT, 2 h, 31–98%; (iii) SOCl_2_ [1.7 eq], pyridine [1.3 eq], DCM, 0 °C, 16 h; (iv) isatin [0.77 eq], K_2_CO_3_ [1.54 eq], KI [0.77 eq], DMF, RT, 16 h, 8–63%.

Novel allosteric modulators were synthesized via two routes. The first route (Fig. 7) started with the radical bromination of para-tolyl acetate with NBS to afford the benzyl bromide compound **9**. Subsequent alkylation with isatin followed by deacetylation produced compound **10**. The final step was a Williamson ether synthesis with the corresponding alkyl chloride to yield **7**. The synthesis of **8a** and related compounds began with commercially available benzaldehydes (Fig. 8). Benzaldehydes with unsubstituted phenols were first alkylated in the presence of base and then reduced with sodium borohydride to afford the corresponding benzyl alcohols **11a–l**. The benzyl alcohols were treated with thionyl chloride to yield benzyl chlorides **12a–l**. Commercially available isatin underwent *n*-alkylation with benzyl halides to produce hybrid compounds **8a–l**.

In vitro evaluation of the library proceeded in two steps: (1) measuring [^3^H]-AA release in the absence and presence of either 0.1 µM or 100 µM ACh and in the absence or presence of potential allosteric modulator (Tables 1–4); and (2) generation of full ACh dose response curves to further characterize some of the more interesting compounds. The first set of studies provided preliminary assessments of the effects of putative allosteric modulators, including intrinsic activity (when measured in the absence of ACh), enhancement of potency (as measured in the presence of 0.1 µM ACh), and enhancement of efficacy (as measured in the presence of 100 µM ACh). Full ACh dose response curves further evaluated the effects of compounds on ACh potency and efficacy.

Within the benzofuran series, **5** increased mAChR activity by 70% compared to treatment with ACh alone (Table 2). However, **5** also exhibited significant intrinsic agonist activity. These preliminary results were encouraging based on the novel observation of positive modulation of ACh activity predominantly at higher doses of ACh. Furthermore, these results strengthened the hypothesis that the electronics of the alkyl-amine are important in modulating mAChR activity.

Initial evaluations of the benzofurans **6a–c** revealed that **6a** was inactive, while **6b** increased ACh activity, and **6c** decreased ACh activity (Table 2). Compound **7** primarily decreased potency and slightly enhanced the efficacy of ACh, whereas **8a** resulted in a significant enhancement of ACh activity. Compound **7** lacked intrinsic activity at the 10 µM concentration. The data from the amiodarone derivatives and **8a** suggested that the aryl iodides ortho to the phenol ether contributed to the modulation of mAChR activity.

A second library was designed featuring a complete series of isatin core-mono/bis o-halogen benzene substitutions (I, Br, Cl, F), **8a–h**, as well as other SAR controls, **8i–k**, to systematically assess the impact of various ring substitutions. An observed increase in ACh activity was favored by bis-aryl-iodide (**8a**) and bis-aryl-bromide (**8c**) substitutions, both demonstrating similar amounts of positive allosteric modulation of ACh activity (Table 3). Compounds **8a–h** were rendered in Spartan’20, which was used to calculate various physical and electronic qualities. Positive correlations were found between the area of the molecules and the associated activity data (Supplemental Fig 1). Correlations were observed at the 10 µM concentration; in the absence of ACh (R^2^ = 81.00%), with 0.1 µM ACh (R^2^ = 94.74%), and with 100 µM ACh (R^2^ = 85.15%). No correlation was observed with any of the calculated electronic values, suggesting that an increase in steric bulk of the halogen substitutions is related to the increase in allosteric modulation of ACh activity.

**Table 3:** Stimulation of ^3^[H]AA release by ACh in the presence and absence of potential *N*-benzyl isatin allosteric modulators of M_1_ receptors expressed in CHO cells. Data are presented as in Table 1, as means ± s.e.m. for 4 or more determinations.

		Assay conditions		
Compound	Structure	No ACh	0.1 µM ACh	100 µM ACh
**7**		0.02 ± 0.01	0.06 ± 0.01	1.11 ± 0.06
**8a**		0.24 ± 0.04	2.44 ± 0.21	1.62 ± 0.06
**8b**		0.08 ± 0.01	2.13 ± 0.08	1.24 ± 0.01
**8c**		0.19 ± 0.03	2.62 ± 0.19	1.42 ± 0.09
**8d**		0.04 ± 0.01	1.57 ± 0.06	0.90 ± 0.03
**8e**		0.13 ± 0.04	2.29 ± 0.14	1.21 ± 0.07
**8f**		0.09 ± 0.04	1.67 ± 0.12	1.08 ± 0.06
**8g**		0.03 ± 0.01	1.41 ± 0.13	0.95 ± 0.05
**8h**		0.05 ± 0.03	1.32 ± 0.10	0.97 ± 0.06

Perusal of the data from the amiodarone series as well as the isatin derivatives, with respect to the observed effects of the aryl-halides as well as the alkyl amines, led to the evaluation of a compound differing only by the heteroaromatic core. Compound **8l** features the isatin heteroaromatic core as in VU0119498, alongside the bis o-halo(bromo) benzene ring and a tertiary alkyl amine as in amiodarone; This compound only decreased ACh activity (Table 4), which was more akin to the negative modulatory effect of **7** and unlike the positive modulatory effect of **8c**. This result further supports the paramount role of the amine in decreasing ACh potency in the evaluated allosteric modulators.

**Table 4:** Stimulation of ^3^[H]AA release by ACh in the presence and absence of potential *N*-benzyl isatin allosteric modulators of M_1_ receptors expressed in CHO cells. Data are presented as in Table 1, as means ± s.e.m. for 4 or more determinations.

		Assay conditions		
Compound	Structure	No ACh	0.1 µM ACh	100 µM ACh
**8i**		0.06 ± 0.01	1.47 ± 0.12	1.04 ± 0.02
**8j**		0.02 ± 0.02	1.09 ± 0.31	0.96 ± 0.01
**8k**		0.03 ± 0.00	1.55 ± 0.07	0.94 ± 0.06
**8l**		0.05 ± 0.01	0.10 ± 0.07	0.86 ± 0.04
**10**		0.01 ± 0.01	1.42 ± 0.24	0.92 ± 0.06

**Figure 9:**
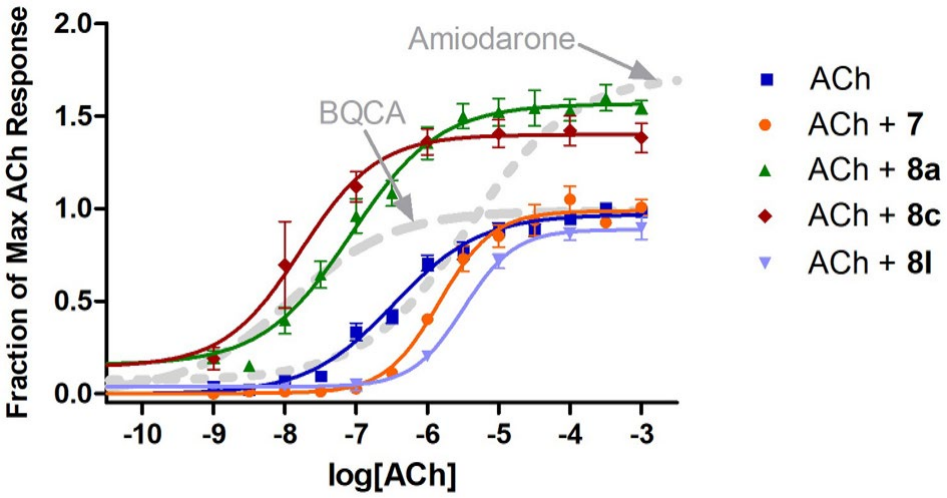
Modulation of ACh response by allosteric modulators at M_1_ receptors. Assays were conducted as in Fig. 3 and the curves for BQCA and amiodarone are included for reference, as in Fig. 3. Each allosteric modulator was included at 10 µM. Data points represent mean ± SEM for four or more determinations. Curves represent the best fits to the four-parameter model described in Methods.

Full ACh dose–response curves were obtained for selected compounds based on the results from the preliminary screen. An interesting range of activities was found as **8a**, **c** dramatically enhanced ACh efficacy, while **7** and **8l** decreased ACh potency with little impact on ACh efficacy (Fig. 9). These SAR studies revealed clear trends within the isatin heteroaromatic core; alkyl amines predominantly lessened ACh potency and, in the absence of alkyl amines, bulkier halides increased ACh efficacy. Within the amiodarone series the trends were clear with one key exception (**2**). Increases in ACh activity occurs with aryl-halides in conjunction with various amines (tertiary, secondary, acyl) although in conjunction with quaternary amines the ACh activity is lessened. Omission of the phenol ether results in a loss of ACh activity modulation, and omission of the aryl-halides results in the loss of ACh efficacy modulation.

**Figure 10:**
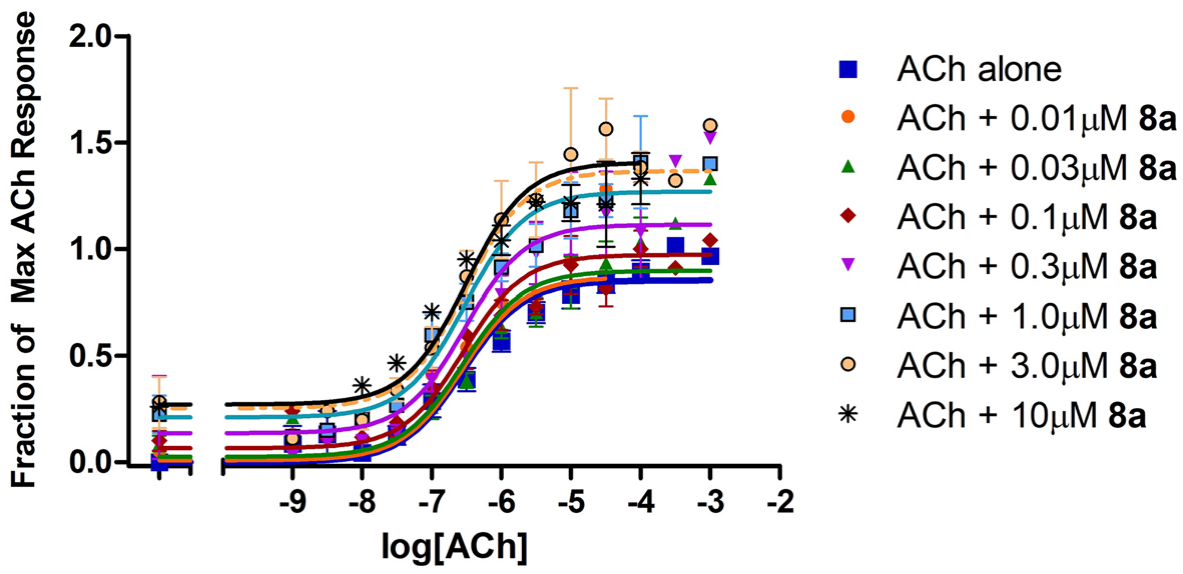
Dose response curves for **8a** at M_1_ receptors Stimulation of [^3^H]-arachidonic acid release by ACh at M_1_ muscarinic receptors expressed in CHO cells in the absence and presence of **8a** from 0.01–10 μM. Assays were conducted as in Fig. 3 and data are presented as the fraction of the maximal response produced by ACh. Curves are based on an allosteric operational model as described in Methods; see Table 5 for best-fit parameters.

Based on the interesting activities observed at the 10 μM ligand concentration, three compounds were evaluated further over a wide range of concentrations to assess the parameters K_B_, τ_B_, α, and β by fitting the data to an allosteric operational model. It is very important to carry out this further modeling because the results of the 4-parameter fitting by itself can be deceiving. For example, cursory evaluation of Fig. 9 might suggest that compounds **8a**, **c** exerted both efficacy effects and affinity effects on the response to ACh. However, it has been well-demonstrated that efficacy-based PAMs exert a concomitant effect on potency, in both 2-state models^20,24^ and in operational models^21^. Indeed, the lack of effect of amiodarone on potency in spite of its strong effect on efficacy has been attributed to a combination of an efficacy-based PAM effect and an affinity-based NAM effect that cancel each other out on the potency axis, at M_3_ muscarinic receptors^24^. Thus, while an increase in the maximal effect is conclusive evidence of an efficacy-based PAM effect, an increase in potency can be ambiguous. Figure 10 shows the results for **8a**, which clearly potentiated the effects of ACh in a dose-dependent manner. The results from the detailed analyses of **7**, **8a**, **c** according to Eq. 2 are summarized in Table 5. As expected, based on the initial studies at 10 µM, both **8a**, **c** exhibited strong activation cooperativity with ACh, with β values over 3, yet little binding cooperativity with α near unity. In contrast, **7** exhibited strong negative binding cooperativity (α < 1) with no impact on activation cooperativity with β near unity.

**Table 5:** Calculated best-fit parameters for three allosteric modulators of ACh at M_1_ muscarinic receptors based on an allosteric operational model.

Characteristic	Parameter	**7**	**8a**	**8c**
Operational efficacy-B	τ_B_	0.032 ± 0.02	0.17 ± 0.03	0.33 ± 0.06
Binding cooperativity, A-B	α	0.18 ± 0.095	0.69 ± 0.24	0.98 ± 0.39
Activation cooperativity, A-B	β	0.99 ± 0.12	3.26 ± 0.43	3.89 ± 0.61
Affinity-B	logK_B_	−6.20 ± 0.33	−6.26 ± 0.15	−5.53 ± 0.15
Goodness of global fit	R^2^	0.923	0.860	0.922

The mean (± s.e.m.) efficacy of ACh (τ_A_) was 0.82 ± 0.09, while the mean (± s.e.m.) affinity of ACh (expressed as logK_A_) was −6.23 ± 0.09, and the efficacy of the system (E_m_) was set to 2.0 across the three evaluations (see “Methods”).

To determine maximum tolerated concentrations and identify target concentrations for behavioral studies, each compound was tested for signs of toxicity (abnormal swimming, startle response or death) in wild-type (AB) zebrafish (*Danio rerio*) over a wide range of concentrations. The MTC was 10 µM for both BQCA and amiodarone, while the MTC was > 30 µM (the highest concentration tested) for both **8a**, **c** (Supplemental Fig. 2). The MTCs for all compounds are shown in Table 6. Amiodarone was the only compound that produced death (at 100 µM).

**Table 6:** Effects of potential allosteric modulators of M_1_ receptors on locomotor activity in zebrafish.

Compound	MTC (µM)	MEC (µM)	Therap. index	Distance moved	Angular velocity	Variance turn angle
Amiodarone	10	> 10	–	85.7 ± 10.2	83.2 ± 12.0	102.2 ± 10.4
Dronedarone	10	10	1	28.3 ± 5.3***	172.8 ± 25.7***	141.6 ± 12.5***
**1**	3	3	1	114.1 ± 8.4	84.5 ± 5.5	84.4 ± 6.3
**2**	1	> 1	–	95.8 ± 11.0	128.9 ± 18.5	97.6 ± 10.3
**3**	3	> 3	–	145.4 ± 21.7	101.8 ± 19.2	93.4 ± 9.4
**4**	0.3	> 0.3	–	56.7 ± 9.3	105.7 ± 14.8	115.8 ± 12.4
**5**	10	> 10	–	108.0 ± 12.9	104.0 ± 10.7	102.5 ± 8.1
**6a**	3	> 3	–	115.4 ± 11.7	95.9 ± 13.3	115.8 ± 13.0
**6b**	30	> 30	–	75.3 ± 8.5*	132.3 ± 16.9	133.7 ± 14.0*
**6c**	3	0.1	30	59.8 ± 8.8	129.9 ± 20.1	136.4 ± 11.8
BQCA	10	3	3.3	72.0 ± 10.0*	141.8 ± 14.9*	119.6 ± 7.0*
VU0238408	30	1	30	34.6 ± 4.0***	180.1 ± 20.3***	146.6 ± 12.4***
**7**	10	> 10	–	72.5 ± 11.8	122.4 ± 6.9	93.9 ± 10.6
**8a**	> 30	0.3	> 100	45.8 ± 6.4***	197.1 ± 19.8***	161.1 ± 13.4***
**8b**	10	1	10	52.7 ± 6.6***	175.3 ± 19.0**	143.0 ± 13.5**
**8c**	> 30	0.1	> 30	26.9 ± 3.7***	201.6 ± 20.2***	150.8 ± 12.0***
**8d**	100	1	100	38.4 ± 4.5***	201.4 ± 27.5***	170.2 ± 15.1***
**8e**	100	1	100	29.1 ± 5.3***	222.1 ± 25.0***	158.8 ± 14.3***
**8f**	10	3	3.3	37.4 ± 6.4***	157.1 ± 24.5*	127.9 ± 16.2
**8g**	30	1	30	65.7 ± 11.7***	144.5 ± 18.6**	126.5 ± 10.4*
**8h**	30	3	10	52.7 ± 6.6***	201.4 ± 27.5**	148.0 ± 12.4***
**8i**	10	1	10	11.6 ± 1.2***	412.1 ± 28.7***	146.1 ± 16.1**
**8k**	30	1	30	66.2 ± 8.4**	154.2 ± 18.0**	126.3 ± 10.8*
**8l**	10	10	1	33.5 ± 4.4**	184.7 ± 17.0***	166.1 ± 12.5***
**10**	10	3	3.3	72.4 ± 8.6*	131.2 ± 13.4*	135.3 ± 13.5*

Maximum tolerated concentration was determined after 24 h exposure. Data represent the percentage change in locomotor behaviors by compounds at 10 µM relative to controls and are presented as means ± s.e.m. N = 12 for each measurement.

*p < 0.05, **p < 0.01, ***p < 0.001 compared to control.

**Figure 11:**
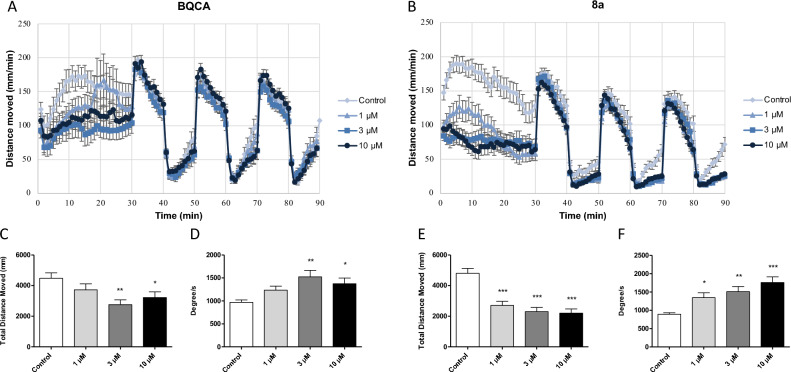
Zebrafish locomotor behavior. Locomotor behavior following administration of (**A**) BQCA and (**B**) **8a**. Distance moved during the 30-min spontaneous swimming period following administration of (**C**) BQCA and (**E**) **8a**. Repetitive behaviors (as measured by angular velocity) during the 30-min spontaneous swimming period following administration of (**D**) BQCA and (**F**) **8a**. Increases in angular velocity reflect a decrease in repetitive behaviors.

All compounds were tested for effects on locomotor activity and turning behaviors in wild-type (AB) zebrafish (*Danio rerio*). BQCA, a well-known, selective PAM of M_1_ muscarinic receptors, was used as a benchmark for these studies. BQCA decreased overall locomotor activity during the 30 min spontaneous swimming period in AB zebrafish(Fig. 11A,C). These data are consistent with a role for M_1_ muscarinic receptors in regulating locomotor activity^5^. BQCA treatment also increased angular velocity (Fig. 11D) and variance of turn angle (data not shown) in AB zebrafish during the 30 min spontaneous swimming period suggesting a decrease in repetitive behaviors. The data for the entire series of compounds are shown in Table 6. Several compounds produced significant decreases in locomotor activity and increases in angular velocity, including compounds **8a–k** within the isatin series. Of note, the novel PAM of ACh efficacy **8a** also reduced locomotor activity (Fig. 11B,E) and increased angular velocity (Fig. 11F) and variance of turn angle (data not shown) in AB zebrafish during the spontaneous swimming period, again reflecting a decrease in repetitive behaviors. In contrast, the negative allosteric modulator of ACh potency **7** was not effective in reducing locomotor activity or elevating angular velocity or variance of turn angle. In addition, none of the compounds in the benzofuran series (i.e., amiodarone derivatives) significantly reduced locomotor activity or decreased repetitive behaviors except for dronedarone and **6b** (Table 4).

Both **8a** and **8c** appeared to be more potent than BQCA with significant effects at 1 μM, which is consistent with their affinities for M_1_ muscarinic receptors (K_B_ values of 550 nM and 3.0 µM, respectively). Additional studies were conducted using lower concentrations of **8a** to establish EC_50_ values for decreasing locomotor activity (5.6 µM) and repetitive behaviors (2.6 µM for increasing angular velocity). Similar studies were conducted with **8c** yielding EC_50_ values for decreasing locomotor activity (1.0 µM) and repetitive behaviors (1.1 µM for increasing angular velocity). In addition, neither **8a** nor **8c** produced any signs of toxicity at concentrations up to 30 μM, the highest concentration tested, suggesting a high therapeutic index.

## Discussion

Previous work identified the unique properties of amiodarone as a positive allosteric modulator of ACh efficacy at muscarinic receptors^24,26^. Although used clinically, amiodarone has several limitations due to its physicochemical properties that contribute to photo- and chemical reactivity, toxicity, and limited CNS activity. The studies described above provide useful information regarding the structural features of amiodarone that contribute to its unique properties as an allosteric modulator of acetylcholine at muscarinic receptors. Six novel benzofuran derivatives and ten isatin derivatives were synthesized and tested for their activities in cells expressing M_1_ muscarinic receptors. Compounds containing di-iodo (**5**, **6b**, and **8a**) and di-bromo (**8c**) substituents exhibited the highest activity in enhancing ACh activity. In contrast, compounds with tertiary amines (i.e., **7** and **8l**) acted as negative allosteric modulators of ACh potency. The studies highlight the importance of the halogens in the positive allosteric modulation of ACh activity by amiodarone and related compounds. In addition, replacement of the benzofuran ring system with isatin afforded active compounds with reduced toxicity in vivo.

At 10 µM, both **8a**, **c** dramatically enhanced ACh activity, shifting the ACh dose response curve to the left and enhancing the maximal response. As discussed previously, the apparent potency enhancement seen with **8a**, **c** appears to be due to their efficacy-based PAM effects and there is no need to invoke affinity-based PAM properties in these cases. In contrast, **7** exhibited no activation cooperativity with ACh with a β value near unity yet displayed negative binding cooperativity with α significantly less than unity. **8a** exhibited lower efficacy (τ_B_) and higher affinity (K_B_) than **8c**. Like **8a**, **7** bound with high affinity, yet it lacked significant efficacy, with τ_B_ near zero.

Taken together with the pharmacological studies, the behavioral studies in zebrafish suggest that allosteric enhancers of ACh efficacy could be useful in alleviating symptoms associated with ASD (and related neurological disorders). A particular advantage of allosteric enhancers of ACh efficacy is the sensitivity of such compounds to levels of receptor reserve^24^. Pathological conditions associated with decreased levels of M_1_ muscarinic receptors, as found in some cases of schizophrenia and in neurodevelopmental disorders^36,47^, would exhibit reduced receptor reserve in specific brain regions. Allosteric enhancers of ACh efficacy could enhance responses in affected tissues (i.e., brain pathways) with minimal impact on tissues with normal muscarinic receptor reserve (e.g., unaffected brain regions or pathways and peripheral targets such as salivary and sweat glands), thereby reducing the potential for adverse effects. Further studies are needed to evaluate effects on repetitive behaviors in other animal models, including mouse models of ASD. Moreover, pharmacological studies of zebrafish muscarinic receptor subtypes are necessary to help verify that the behavioral effects of compounds are related to the activities observed at human M_1_ receptors. Finally, evaluations of compound activity at other mAChRs are needed to assess selectivity for M_1_ receptors.

In summary, evaluation of novel mAChR allosteric ligands elucidated key structural motifs that result in distinct allosteric profiles. Principally the bis-ortho iodo/bromo phenol ether exhibited a pronounced positive activation cooperativity, while the phenoxy ethylamine produced negative binding cooperativity. Combination of these structural motifs did not result in additive effects; rather the negative allosteric modulatory effects on potency predominated with the isatin heteroaromatic derivative (**8l**) while the positive allosteric modulatory effects predominated within the benzofuran series (amiodarone). Most importantly, we report two novel drug-like compounds **8a**, **c** that display positive activation cooperativity with ACh at the M_1_ mAChR, demonstrate low in vivo toxicity, and decrease repetitive behaviors in the zebrafish model. These findings provide unique insights into the design of mAChR allosteric therapeutic ligands for disease states associated with deficits/alterations in cholinergic signaling. Subsequent studies will evaluate the existing library and second-generation compounds incorporating similar substructures for receptor subtype selectivity, activity, and brain permeability in rodent models of ASD.

## Methods

### General

Efforts were made to adhere to the essential 10 ARRIVE guidelines, including appropriate control groups, inclusion of all data from each experiment, randomization of treatments, blinding of researchers to the identity of compounds for behavioral studies, the use of one-way and two-way ANOVA with follow-up Tukey tests for individual comparisons, and the expression of results as the mean (± s.e.m) for each treatment^48^.

### Cell culture

CHO cells expressing human M_1_ muscarinic receptors were cultured at 37 °C in a 5% CO_2_ atmosphere in F12 medium supplemented with 5% fetal bovine serum, 100 units/ml penicillin, and 100 µg/ml streptomycin. These cells were obtained from Mark Brann’s laboratory when he was at NIH and have been in use by us and others for many years^49^.

### Arachidonic acid release

Measurement of [^3^H]AA release was performed as described in Stahl and Ellis^24^. Stably transfected CHO-hM_1_ cells were seeded on 24-well plates (Greiner Bio-One GmbH, Frickenhausen, Germany) at a density of 5.0 × 10^4^ cells/well in 0.5 ml of F-12 medium. Cells were incubated until they attached (approximately 3 h), followed by the addition of [^3^H]AA to a final concentration of 0.05 μCi of [^3^H]AA per well. The cells were then grown for 16 to 20 h before the assay was performed. [^3^H]AA release was measured in Eagle’s basal medium with 20 mM HEPES and 2 mg/ml fatty acid-free bovine serum albumin (EM-BSA). Cells were rinsed twice with EM-BSA, followed by the addition of EM-BSA media containing experimental agents and incubated for 1 h at 37 °C. The assay was terminated by aspiration of the media, and the amount of [^3^H]AA released was determined by liquid scintillation counting (Beckman-Coulter LS6500).

Where indicated (Fig. 2), cells were pretreated with POB to reduce the number of available receptors per cell. Stock POB was made up at 10 mM in ethanol and stored at −20C. Each well was pretreated with 0.5 ml of the appropriate concentration in PBS with 1 mM CaCl_2_ and 1 mM MgCl_2_ (PBS+ +) for 30 min at 37 °C, followed by 5 washes with PBS+  + . After pretreatment, the normal protocol follows as above.

### In vitro pharmacological data analysis

Tabulated [^3^H]AA release data are represented as a fraction of maximal release elicited by ACh or as the fraction of release elicited by the relevant concentration of ACh alone, as indicated. Dose-response curves were normalized to the maximal response of ACh without allosteric ligand. Where indicated, response curves were analyzed using the empirical four-parameter equation:

$$Y = Top + \frac{Bottom - Top}{{1 + \left( {\frac{X}{{C_{50} }}} \right)^{n} }}$$

where X is the concentration of the ligand used, Y is the amount of response, C_50_ is the concentration of the ligand that produces 50% of the maximal effect, *Top* and *Bottom* are the top and bottom plateaus of the curve, and n is related to the Hill slope for the curve. Where indicated, families of response curves were analyzed according to an allosteric operational model^50,51^:

$$Y = E_{M} \frac{{[\tau_{A} A(K_{B} + \alpha \beta B) + \tau_{B} BK_{A} ]}}{{[\tau_{A} A(K_{B} + \alpha \beta B) + \tau_{B} BK_{A} + AK_{B} + K_{A} K_{B} + K_{A} B + \alpha AB]}}$$

where E_M_ is the maximal response; A is the concentration of ACh; B is the concentration of the allosteric ligand; τ_A_ and τ_B_ represent the operational efficacies of A and B, respectively; K_A_ and K_B_ are the equilibrium dissociation constants of A and B; and α and β represent the cooperativities between A and B in terms of binding and activation, respectively. Curve-fitting was carried out with GraphPad Prism, version 5.0 or 6.0 (San Diego CA).

A rigorous and laborious method has been described to attempt to overcome the interdependencies of several of the parameters in this allosteric operational model^50,51^. To accomplish this, the authors employed receptor-G-protein fusion proteins. This method was deemed to be beyond the scope of the present study. Instead, we fitted the above parameters as variables simultaneously, with the exception of E_M_. Because we wanted the maximal effect of ACh to be unity (in agreement of the 4-parameter fits) and because we had seen amiodarone and the other efficacy PAMs approach but not exceed a doubling of the maximal response of ACh, we set the value of E_M_ at 2 for these fits. We are not aware of any muscarinic arachidonic acid release data having been previously fitted to an allosteric operational model.

### Animals and husbandry

Larval zebrafish were produced in the University of Toledo zebrafish core facility and housed in an incubator maintained at 28 °C on a 14:10 h cycle. Fish were acclimated to the testing room conditions the evening prior to testing. All testing took place during the 14 h light cycle after the lights had been on for at least 2 h. All experiments and animal husbandry practices were approved by the University of Toledo Institutional Animal Care and Use Committee (IACUC #400091) and all experiments were performed in accordance with the relevant guidelines and regulations.

### Maximum tolerated concentration (MTC) determination

Determination of maximum tolerated concentration (adapted from Berghmans)^52^ was completed by placing 5 days post fertilization (dpf) wild-type zebrafish larvae in a 24 well plate at 6 fish per well, and one compound concentration per well. Seven concentrations were tested ranging from 0.1 μM to 1 mM at half log intervals, solubility permitting. Death was determined by the absence of heartbeat. Additional endpoints were used to assess toxicity at non-lethal concentrations such as startle capacity (poke stimulus), abnormalities in swimming behavior (loss of dorsoventral balance), and gross morphological deformities (bent body). The MTC was defined as the highest concentration that did not cause death and where not more than 2 out of 12 larvae exhibited any sign of locomotor impairment, including no touch response after 24 h. The MTC determination was used to select three doses of each compound (low, medium, high dose) to be used in behavioral assays.

### General locomotor assay

The movement of larval zebrafish was monitored with the Noldus behavior recording system (Noldus Information Technology, Leesburg VA) and quantified using EthoVision® XT 15 software (Noldus Information Technology, Leesburg VA). Five-dpf zebrafish larvae were exposed to a range of nonlethal concentrations of compounds in a 24-well plate with appropriate controls. The plate was immediately placed in the incubator for a 30-min exposure period. After 30 min, the plate was transferred to the Noldus system and swimming behavior was recorded in 100% light for 30 min to measure spontaneous swimming behavior. The light was switched off for 10 min (dark period) and then switched on for 10 min (light period), which was then repeated for two additional cycles. In total, the larvae were exposed to three cycles of alternating 10 min dark and light periods (100% darkness-to-100% light). Each drug challenge was conducted on three separate plates with n = 6 larvae/dose/plate.

Absolute turn angle (TA) and angular velocity provided measures of the extent of turning behavior, while the intra-individual variance of turn angle quantified how consistently (low variance) or inconsistently (high variance) fish turned. A lowered intra-individual variance of turning reflected repeatedly performing the same turning behavior, a stereotypy^53^.

### Zebrafish behavior statistical analysis

All data for exposure and the behavioral end points were analyzed by one-way ANOVA (factor: drug concentration) followed by a Dunnett’s multiple comparisons test for post hoc significance between drug concentration and control group. Statistically significant differences are noted as follows; *p < 0.05; **p < 0.01; ***p < 0.001.

### General synthetic methods

Reagents and solvents were purchased from common commercial suppliers Fisher (Durham, NC), or Sigma-Aldrich (St. Louis, MO) and used as received. All reactions were carried out under atmospheric conditions at room temperature unless otherwise indicated. Reactions were monitored by thin-layer chromatography (TLC, LuxPlate silica gel 60 F_254_ plates) and revealed by UV light (254 nm). Column chromatography was performed using Teledyne Combiflash R_f_ with RediSepR_f_ Gold columns. HPLC analysis was performed using a Shimadzu Prominence HPLC (LCD-20AD) with temperature controlled autosampler (SIL-20AC), refractive index (RID-20A) and PDA (SPD-M20A) detectors. Separations utilized a Phenomenex Kinetix® core column (2.6 µm, C18, 100 Å, 100 × 4.6 mm column). HPLC conditions: Mobile phase A = H_2_O (0.1% formic acid [FA]) and mobile phase B = acetonitrile (0.1% FA); 1.0 ml/min at 30% B for 1 min followed by gradient increase to 95% B over 6 min followed by 1 min at 95% B and re-equilibration at 30% B for 4 min resulting in a total run time of 12 min. Purity of tested compounds was found to be > 95% pure at two wavelengths, 254 and 280 nm unless otherwise indicated, or by elemental analysis performed by Atlantic Microlabs (Norcross, GA). NMR (^1^H, ^13^C) spectra were taken using a Bruker Avance 600 MHz spectrometer (cryoprobe). High resolution mass spectra (HRMS) were recorded using Waters Synapt high-definition mass spectrometer (HDMS) equipped with nano-ESI source positive mode. Compound **5** has cis and trans isomeric states that interconvert, each with distinctive NMR spectra^54,55^.

### Synthetic chemistry

(2-butylbenzofuran-3-yl)(4-(2-(ethylamino)ethoxy)-3,5-diiodophenyl)methanone hydrochloride **(1)**. Amiodarone HCl (500.0 mg, 733.4 µmol) was stirred in a biphasic solution of EA (30 ml) and saturated NaHCO_3_ (aq, 20 ml) for 1 h. The EA layer was washed with brine, dried over anhydrous Na_2_SO_4_, and concentrated *in vacuo* to afford a yellow oil, amiodarone free base. The residue was taken up in DCE (10 ml) and cooled down to 0 °C with stirring. To this solution 1-chloroethyl carbonochloridate (395.6 µL, 3.7 mmol) was added and allowed to stir for 30 min. The reaction mixture was then heated to reflux for 1 h. After cooling to room temperature, the reaction mixture was concentrated *in vacuo*, dissolved in anhydrous MeOH (10 ml), and refluxed with stirring for 1 h. After cooling to room temperature, the reaction mixture was concentrated *in vacuo* and purified by flash chromatography (SiO_2_, EA/MeOH;0–30%) to afford **1** as white crystals (75.6 mg, 15.8%). ^1^H NMR (DMSO-d_6_, 600 MHz): *δ* 8.99 (2H, b); 8.20 (2H, s); 7.68–7.67 (1H, d, *J* = 8.22 Hz); 7.51–7.50 (1H, d, *J* = 7.8 Hz); 7.39–7.37 (1H, m); 7.32–7.30 (1H, m); 4.28–4.27 (2H, m); 3.49–3.47 (2H, t, *J* = 4.38 Hz); 3.16–3.12 (2H, q, *J* = 7.14 Hz); 2.73–2.70 (2H, t, *J* = 7.62 Hz); 1.72–1.67 (2H, quint, *J* = 7.56 Hz); 1.30–1.23 (5H, m); 0.86–0.84 (3H, t, *J* = 7.32 Hz). ^13^C NMR (DMSO-d_6_, 600 MHz): *δ* 187.9, 166.1, 160.4, 153.5, 140.2, 139.2, 126.6, 125.4, 124.4, 121.3, 116.0, 111.7, 92.7, 68.6, 46.6, 42.8, 40.5, 29.8, 28.0, 22.5, 13.9, 11.5. Measured purity by elemental analysis: calculated for (C: 42.27, H: 3.98, N: 2.14, Cl: 5.43, I: 38.84); found (C: 42.06, H: 3.86, N: 2.11, Cl: 5.43, I: 38.71).

N-(2-(4-(2-butylbenzofuran-3-carbonyl)-2,6-diiodophenoxy)ethyl)-N-ethylacetamide **(5)**. **1** (250.0 mg, 382.4 µmol) was dissolved in DCM (5 ml) and cooled to 0 °C. Et_3_N (117.3 µL, 841.3 µmol) was added to the reaction mixture and stirred for 10 min, then Ac_2_O (54.2 µL, 573.6 µmol) was added and the mixture was raised to room temperature over 16 h. The reaction mixture was concentrated *in vacuo* and purified by flash chromatography (SiO_2_, Hex/EA;0–30%) to afford **5** as a yellow oil (102.7 mg, 40.7%). ^1^H NMR (CDCl_3_ DMSO-d_6_, 600 MHz): *δ* 8.20–8.19 (2H, m); 7.48–7.46 (1H, m); 7.39–7.38 (1H, d, *J* = 7.84z); 7.31–7.27 (1H, m); 7.24–7.21 (1H, m); 4.22–4.20 (1.3H, t, *J* = 5.10 Hz); 4.15–4.14 (0.7H, t, *J* = 5.83 Hz); 3.83–3.80 (2H, m); 3.61–3.53 (2H, m); 2.86–2.82 (2H, q, *J* = 6.98 Hz); 2.24 (1H, s); 2.15 (2H, s); 1.78–1.73 (2H, m); 1.38–1.31 (2H, m); 1.28–1.25 (2H, t, *J* = 6.98 Hz); 1.19–1.17 (1H, t, *J* = 7.76 Hz); 0.91–0.89 (3H, m). ^13^C NMR (CDCl_3_ DMSO-d_6_, 600 MHz): *δ* 187.7, 187.6, 170.6, 166.3, 166.2, 160.6, 160.2, 153.6, 140.7, 138.7, 138.4, 126.3, 124.7, 123.8, 121.0, 115.8, 115.7, 111.1, 90.8, 90.7, 71.8, 70.5, 47.5, 45.6, 45.5, 41.2, 30.0, 28.2, 22.5, 22.0, 21.4, 14.1, 13.7, 12.8. Measured purity at 254 nm: 97.32%; 280 nm: 99.9%. HRMS: calculated for (C_25_H_27_I_2_NO_4_): 659.0029, found 681.9944 (M + Na^+^).

(2-butylbenzofuran-3-yl)(4-methoxyphenyl)methanone **(6a)**. Commercially available **4** (1.00 g, 3.4 mmol) was dissolved in anhydrous DMF (10 ml), Cs_2_CO_3_ (2.21 g, 6.8 mmol) was added, and heated to 60 °C. To the reaction mixture methyl iodide (846.0 µL, 13.6 mmol) was added and stirred for 1 h. After cooling to room temperature, the reaction mixture was quenched with dH_2_O (20 ml) and extracted with EA (2 × 20 ml). The combined organic extracts were washed with dH_2_O (2 × 20 ml), brine (20 ml), dried over anhydrous Na_2_SO_4_, and concentrated *in vacuo* to afford a yellow oil. The residue was purified by flash chromatography (SiO_2_, Hex/EA;0–30%) to afford **6a** as a clear oil (621.8 mg, 59.2%). ^1^H NMR (CDCl_3_, 600 MHz): *δ* 7.86–7.84 (2H, m); 7.49–7.47 (1H, d, *J* = 8.22 Hz); 7.37–7.35 (1H, d, *J* = 7.82 Hz); 7.29–7.26 (1H, m); 7.20–7.17 (1H, m); 6.98–6.95 (2H, m); 3.90 (3H, s); 2.93–2.90 (2H, *J* = 7.49 Hz); 1.78–1.73 (2H, quint, *J* = 8.02 Hz); 1.39–1.33 (2H, sext, *J* = 7.23 Hz); 0.91–0.88 (3H, t, *J* = 8,02 Hz). ^13^C NMR (CDCl_3_, 600 MHz): *δ* 190.5, 164.7, 163.4, 153.5, 131.8, 131.7, 127.1, 124.1, 123.3, 121.2, 116.7, 113.6, 110.9, 55.5, 30.1, 27.8, 22.3, 13.7. Measured purity at 254 nm: 95.6%; 280 nm: 95.9%. HRMS: calculated for (C_20_H_20_O_3_): 308.1412, found 309.1501 (M + H^+^).

(2-butylbenzofuran-3-yl)(3,5-diiodo-4-methoxyphenyl)methanone** (6b)**. Commercially available **3** (1.21 g, 2.2 mmol) was dissolved in anhydrous DMF (10 ml), K_2_CO_3_ (612.4 mg, 4.4 mmol) was added, and heated to 60 °C. To the reaction mixture methyl iodide (551.7 µL, 8.9 mmol) was added and stirred for 1 h. After cooling to room temperature, the reaction mixture was quenched with dH_2_O (20 ml) and extracted with EA (2 × 20 ml). The combined organic extracts were washed with dH_2_O (2 × 20 ml), brine (20 ml), dried over anhydrous Na_2_SO_4_, and concentrated *in vacuo* to afford a yellow oil. The residue was purified by flash chromatography (SiO_2_, Hex/EA;0–30%) to afford **6b** as a yellow oil (495.1 mg, 39.9%). ^1^H NMR (CDCl_3_, 600 MHz): *δ* 8.22 (2H, s); 7.50–7.49 (1H, d, *J* = 8.21 Hz); 7.44–7.43 (1H, d, *J* = 7.30 Hz); 7.33–7.30 (1H, m); 7.27–7.24 (2H, m); 3.95 (3H, s); 2.87–2.85 (2H, t, *J* = 7.69 Hz); 1.81–1.75 (2H, m); 1.40–1.34 (2H, sext, *J* = 7.41 Hz); 0.94–0.91 (3H, t, *J* = 7.41 Hz). ^13^C NMR (CDCl_3_, 600 MHz): *δ* 187.8, 166.2, 162.3, 153.6, 140.7, 138.4, 126.4, 124.7, 123.8, 121.0, 115.8, 111.1, 90.5, 60.8, 30.0, 28.2, 22.5, 13.7. Measured purity at 254 nm: 95.7%; 280 nm: 98.9%. HRMS: calculated for (C_20_H_18_I_2_O_3_): 559.9345, found 560.9465 (M + H^+^).

(2-butylbenzofuran-3-yl)(4-(2-(diethylamino)ethoxy)phenyl)methanone **(6c)**. Commercially available **4** (1.00 g, 3.4 mmol) was dissolved in anhydrous DMF (10 ml), Cs_2_CO_3_ (4.43 g, 13.6 mmol) was added, and heated to 60 °C. To the reaction mixture 2-chloro-N,N-diethylethan-1-amine hydrochloride (584.7 mg, 3.4 mmol) was added and stirred for 1 h. After cooling to room temperature, the reaction mixture was quenched with dH_2_O (20 ml) and extracted with EA (2 × 20 ml). The combined organic extracts were washed with dH_2_O (2 × 20 ml), brine (20 ml), dried over anhydrous Na_2_SO_4_, and concentrated *in vacuo* to afford a yellow oil. The residue was purified by flash chromatography (SiO_2_, Hex/EA;0–30%) to afford **6c** as a yellow oil (671.5 mg, 50.1%). ^1^H NMR (CDCl_3_, 600 MHz): *δ* 7.83–7.81 (2H, d, *J* = 8.72 Hz); 7.47–7.46 (1H, d, *J* = 8.20 Hz); 7.35–7.34 (1H, d, *J* = 7.65 Hz); 7.27–7.25 (1H, m); 7.19–7.16 (1H, m); 6.96–6.94 (2H, d, *J* = 8.74 Hz); 4.13–4.11 (2H, t, *J* = 6.19 Hz); 2.91–2.89 (4H, t, *J* = 6.73 Hz); 2.67–2.63 (4H, q, *J* = 7.40 Hz); 1.77–1.72 (2H, m); 1.37–1.32 (2H, m); 1.09–1.07 (6H, t, *J* = 7.73 Hz); 0.90–0.87 (3H, t, *J* = 7.73 Hz). ^13^C NMR (CDCl_3_, 600 MHz): *δ* 190.5, 164.6, 162.8, 153.5, 131.8, 131.6, 127.2, 124.1, 123.3, 121.2, 116.7, 114.2, 110.9, 66.9, 51.5, 47.9, 30.1, 27.8, 22.3, 13.7, 11.8. Measured purity at 254 nm: 99.1%; 280 nm: 99.2%. HRMS: calculated for (C_25_H_31_NO_3_): 393.2304, found 416.2211 (M + Na^+^).

4-(bromomethyl)phenyl acetate **(9)**. p-tolyl acetate (11.28 g, 75.11 mmol) was dissolved in CCl_4_ (100 ml), then NBS (14.71 g, 82.6 mmol) and AIBN (616.7 mg, 3.8 mmol) were added. The reaction mixture was heated to reflux for 1 h, then cooled to room temperature, and solids were filtered off. The filtrate was washed with saturated Na_2_HCO_3_ (aq, 2 × 100 ml), brine (100 ml), dried over anhydrous Na_2_SO_4_, and concentrated *in vacuo* to afford **9** as white crystals (14.76 g, 85.8%). **9** was immediately used in the next synthetic step.

1-(4-hydroxybenzyl)indoline-2,3-dione **(10)**. **9** (8.30 g, 36.2 mmol) was dissolved in DMF (10 ml) and added to a heterogenous mixture of isatin (5.86 g, 39.9 mmol) and K_2_CO_3_ (11.02 g, 79.7 mmol) in DMF (25 ml). The reaction mixture was stirred at room temperature for 4 h, quenched with dH_2_O (100 ml), and extracted with EA (2 × 100 ml). The combined organic extracts were washed with brine (100 ml), dried over anhydrous Na_2_SO_4_, and concentrated *in vacuo* to afford a red crystal. The residue was dissolved in MeOH (60 ml), then K_2_CO_3_ (10.02 g, 72.5 mmol) was added and stirred at room temperature for 2 h. The reaction mixture was quenched with 1N HCl (50 ml), extracted with EA (2 × 100 ml), dried over anhydrous Na_2_SO_4_, concentrated *in vacuo,* and purified by flash chromatography (SiO_2_, DCM/EA;0–40%) to afford **10** as a red crystal (2.30 g, 25.1%). ^1^H NMR (DMSO-d_6_, 600 MHz): *δ* 9.41 (1H, s); 7.59–7.54 (2H, m); 7.23–7.21 (2H, d, *J* = 8.74 Hz); 7.11–7.08 (1H, m); 7.00–6.99 (1H, d, *J* = 7.87 Hz); 6.71–6.70 (2H, d, *J* = 8.74 Hz); 4.77 (2H, s). ^13^C NMR (DMSO-d_6_, 600 MHz): *δ* 183.7, 158.6, 157.3, 150.8, 138.4, 129.3, 125.9, 124.9, 123.7, 118.1, 115.8, 111.6, 42.9. Measured purity by elemental analysis: calculated for (C: 71.14, H: 4.38, N: 5.53); found (C: 70.65, H: 4.26, N: 5.54). HRMS: calculated for (C_15_H_11_NO_3_): 253.0739, found 276.0641 (M + Na^+^).

1-(4-(2-(diethylamino)ethoxy)benzyl)indoline-2,3-dione **(7)**. **10** (300.0 mg, 1.9 mmol) was dissolved in DMF (10 ml), then Cs_2_CO_3_ (1.54 g, 4.7 mmol) was added, and heated to 60 °C. To the reaction mixture 2-chloro-N,N-diethylethan-1-amine hydrochloride (203.9 mg, 1.2 mmol) and NaI (cat) were added and allowed to stir for 2 h. The reaction mixture was cooled to 35 °C and quenched with 1N HCl (5 ml), then cooled to room temperature, washed with saturated NaHCO_3_, and extracted with EA (3 × 20 ml). The organic extracts were washed with dH_2_O (30 ml), brine (30 ml), dried over anhydrous Na_2_SO_4_, concentrated *in vacuo,* and purified by flash chromatography (SiO_2_, DCM/MeOH;0–10%) to afford **7** as a red wax (81.5 mg, 19.5%). ^1^H NMR (CDCl_3_, 600 MHz): *δ* 7.54–7.53 (1H, d, *J* = 6.96 Hz); 7.46–7.43 (1H, m); 7.23–7.21 (2H, d, *J* = 8.12 Hz); 7.05–7.02 (1H, m); 6.84–6.82 (2H, m); 6.78–6.76 (1H, d, *J* = 8.12 Hz); 4.81 (2H, s); 4.00–3.97 (2H, m); 2.84–2.82 (2H, m); 2.62–2.58 (4H, m); 1.04–1.01 (6H, m). ^13^C NMR (CDCl_3_, 600 MHz): *δ* 183.4, 158.6, 158.2, 150.7, 138.3, 128.8, 126.4, 125.3, 123.7, 117.6, 114.9, 111.0, 66.4, 51.5, 47.6, 43.5, 11.6. Measured purity by elemental analysis: calculated for (C: 71.57, H: 6.86, N: 7.95); found (C: 70.99, H: 6.69, N: 7.67). HRMS: calculated for (C_21_H_24_N_2_O_3_): 352.1787, found 353.1857 (M + H^+^).

General phenol alkylation procedure: The appropriate phenol (1.0 eq) was dissolved in DMF, then K_2_CO_3_ (2.0—4.0 eq) was added. After the reaction mixture was heated to 60 °C the corresponding alkyl halide (1.0—4.0 eq) was added and allowed to stir for 2 h. After cooling to room temperature, the reaction mixture was quenched with dH_2_O and extracted with EA. The organic extracts were washed with DI, brine, dried over anhydrous Mg_2_SO_4_, concentrated *in vacuo*, and filtered through a pad of celite to afford the corresponding phenol ethers as described below.

General reduction procedure: The appropriate benzaldehyde (1.0 eq) was dissolved in anhydrous MeOH and cooled to 0 °C. NaBH_4_ (1.5 eq) was added in two parts over 30 min. After stirring for an additional 30 min, the reaction mixture was quenched with dH_2_O and extracted with EA. The organic extracts were washed with DI, brine, dried over anhydrous Na_2_SO_4_, concentrated *in vacuo*, and filtered through a pad of celite to afford the corresponding benzyl alcohols as described below.

(3,5-diiodo-4-methoxyphenyl)methanol** (11a)**. Synthesized using the general phenol alkylation procedure with the following quantities: 4-hydroxy-3,5-diiodobenzaldehyde (2.00 g, 5.35 mmol); K_2_CO_3_ (2.96 g, 21.40 mmol); methyl iodide (1.33 ml, 21.40 mmol); afforded 3,5-diiodo-4-methoxybenzaldehyde as a white solid (1.86 g, 89.4%). ^1^H NMR (DMSO-d_6_, 600 MHz): *δ* 9.83 (1H, s); 8.31 (2H, s); 3.81 (3H, s). ^13^C NMR (DMSO-d_6_, 600 MHz): *δ* 190.3, 163.5, 141.0, 135.9, 92.9, 60.9.

Synthesized using the general reduction procedure with the following quantities: 3,5-diiodo-4-methoxybenzaldehyde (1.80 g, 4.64 mmol); NaBH_4_ (263.3 mg, 6.96 mmol); afforded **11a** as white solid (1.62 g, 89.7%). ^1^H NMR (DMSO-d_6_, 600 MHz): *δ* 7.74 (2H, s); 5.33–5.31 (1H, t, *J* = 5.42 Hz); 4.40–4.39 (2H, d, *J* = 4.70 Hz); 3.72 (3H,s). ^13^C NMR (DMSO-d_6_, 600 MHz): *δ* 157.4, 143.3, 137.8, 91.5, 61.1, 60.7.

(3-iodo-4-methoxyphenyl)methanol **(11b)**. Synthesized using the general reduction procedure with the following quantities: 3-iodo-4-methoxybenzaldehyde (1.00 g, 3.82 mmol); NaBH_4_ (216.6 mg, 5.72 mmol); afforded **11b** as a white solid (715.2 mg, 70.8%). ^1^H NMR (DMSO-d_6_, 600 MHz): *δ* 7.70 (1H, d, *J* = 2.06 Hz); 7.29–7.27 (1H, dd); 6.95–6.94 (1H, d, *J* = 8.43 Hz); 5.16–5.14 (1H, t, *J* = 7.13 Hz); 4.39–4.38 (2H, d, *J* = 5.71 Hz); 3.79 (3H, s). ^13^C NMR (DMSO-d_6_, 600 MHz): *δ* 157.0, 137.6, 137.3, 128.4, 111.6, 86.1, 62.1, 56.8.

(3,5-dibromo-4-methoxyphenyl)methanol **(11c)**. Synthesized using the general phenol alkylation procedure with the following quantities: 4-hydroxy-3,5-dibromobenzaldehyde (3.00 g, 10.72 mmol); K_2_CO_3_ (5.92 g, 42.87 mmol); methyl iodide (2.67 ml, 42.87 mmol); afforded 3,5-dibromo-4-methoxybenzaldehyde as an off-yellow solid (2.83 g, 89.7%). ^1^H NMR (DMSO-d_6_, 600 MHz): *δ* 9.88 (1H, s); 8.16 (2H, s); 3.87 (3H, s). ^13^C NMR (DMSO-d_6_, 600 MHz): *δ* 190.5, 158.5, 134.9, 134.2, 119.0, 61.2.

Synthesized using the general reduction procedure with the following quantities: 3,5-dibromo-4-methoxybenzaldehyde (2.80 g, 9.53 mmol); NaBH_4_ (540.6 mg, 14.29 mmol); afforded **11c** as an off-yellow solid (2.77 g, 98.2%). ^1^H NMR (DMSO-d_6_, 600 MHz): *δ* 7.57 (2H, s); 5.41–5.39 (1H, m); 4.45–4.44 (2H, d, *J* = 5.92 Hz); 3.77 (3H, s). ^13^C NMR (DMSO-d_6_, 600 MHz): *δ* 152.3, 142.6, 130.9, 117.6, 61.5, 60.8.

(3-bromo-4-methoxyphenyl)methanol **(11d)**. Synthesized using the general reduction procedure with the following quantities: 3-bromo-4-methoxybenzaldehyde (2.00 g, 9.30 mmol); NaBH_4_ (527.8 mg, 13.95 mmol); afforded **11d** as an off-yellow oil (1.95 g, 96.5%). ^1^H NMR (CDCl_3_ DMSO-d_6_, 600 MHz): *δ* 7.52 (1H, d, *J* = 2.06 Hz); 7.27–7.26 (1H, dd); 7.02–7.01 (1H, d, *J* = 8.45 Hz); 5.25–5.23 (1H, t, *J* = 5.85 Hz); 4.45–4.44 (2H, d, *J* = 6.67 Hz); 3.81 (3H, s). ^13^C NMR (CDCl_3_ DMSO-d_6_, 600 MHz): *δ* 154.62, 136.7, 131.5, 127.5, 112.6, 110.7, 62.3, 56.5.

(3,5-dichloro-4-methoxyphenyl)methanol** (11e)**. Synthesized using the general phenol alkylation procedure with the following quantities: 4-hydroxy-3,5-dichlorobenzaldehyde (1.00 g, 5.24 mmol); K_2_CO_3_ (2.90 g, 20.94 mmol); methyl iodide (1.35 ml, 20.94 mmol); afforded 3,5-dichloro-4-methoxybenzaldehyde as an off-yellow oil (773.8 mg, 72.0%). ^1^H NMR (DMSO-d_6_, 600 MHz): *δ* 9.90 (1H, s); 8.01 (2H, s); 3.91 (3H, s). ^13^C NMR (DMSO-d_6_, 600 MHz): *δ* 190.7, 156.6, 133.9, 130.5, 129.8, 61.4.

Synthesized using the general reduction procedure with the following quantities: 3,5-dichloro-4-methoxybenzaldehyde (758.2 mg, 3.70 mmol); NaBH_4_ (209.9 mg, 5.55 mmol); afforded **11e** as an off-yellow oil (639.4 mg, 83.5%). ^1^H NMR (DMSO-d_6_, 600 MHz): *δ* 7.38 (2H, s); 5.42–5.40 (1H, t, *J* = 5.77 Hz); 4.45–4.44 (2H, d, *J* = 6.17 Hz); 3.79 (3H, s). ^13^C NMR (DMSO-d_6_, 600 MHz): *δ* 150.4, 141.5, 128.4, 127.2, 61.7, 60.9.

(3-chloro-4-methoxyphenyl)methanol** (11f)**. Synthesized using the general phenol alkylation procedure with the following quantities: 3-chloro-4-hydroxybenzaldehyde (880.0 mg, 5.62 mmol); K_2_CO_3_ (3.11 g, 22.48 mmol); methyl iodide (1.40 ml, 22.48 mmol); afforded 3-chloro-4-methoxybenzaldehyde as a white solid (748.0 mg, 78.0%). ^1^H NMR (CDCl_3_, 600 MHz): *δ* 9.69 (1H, s); 7.71 (1H, d, *J* = 1.95 Hz); 7.62–7.60 (1H, dd); 6.92–6.90 (1H, d, *J* = 8.36 Hz); 3.84 (3H, s). ^13^C NMR (CDCl_3_, 600 MHz): *δ* 189.6, 159.6, 130.7, 130.6, 130.1, 123.4, 111.6, 56.4.

Synthesized using the general reduction procedure with the following quantities: 3-chloro-4-methoxybenzaldehyde (748.0 mg, 4.38 mmol); NaBH_4_ (248.8 mg, 6.58 mmol); afforded **11f.** as a clear oil (740.2 mg, 97.8%). ^1^H NMR (CDCl_3_, 600 MHz): *δ* 7.30–7.29 (1H, d, *J* = 2.07 Hz); 7.14–7.12 (1H, dd); 6,85–6.83 (1H, d,* J* = 8.27 Hz); 4.49 (2H, s); 3.84 (3H, s); 2.83 (1H, b). ^13^C NMR (CDCl_3_, 600 MHz): *δ* 154.3, 134.1, 129.0, 126.5, 122.2, 111.9, 64.0, 56.1.

(3,5-difluoro-4-methoxyphenyl)methanol** (11 g)**. Synthesized using the general reduction procedure with the following quantities: 3,5-difluoro-4-methoxybenzaldehyde (600.0 mg, 3.49 mmol); NaBH_4_ (197.8 mg, 5.23 mmol); afforded **11 g** as a yellow oil (468.1 mg, 77.1%). ^1^H NMR (DMSO-d_6_, 600 MHz): *δ* 7.15–7.13 (1H, d, *J* = 11.68 Hz); 7.08–7.04 (2H, m); 5.27–5.25 (1H, t, *J* = 5.74 Hz); 4.46–4.45 (2H, d, *J* = 5.81 Hz); 3.80 (3H, s). ^13^C NMR (DMSO-d_6_, 600 MHz): *δ* 156.2, 154.6, 139.5–139.4, 134.6–134.4, 110.4–110.2, 62.1, 61.9.

(3-fluoro-4-methoxyphenyl)methanol **(11h)**. Synthesized using the general reduction procedure with the following quantities: 3-fluoro-4-methoxybenzaldehyde (1.00 g, 6.49 mmol); NaBH_4_ (368.2 mg, 9.73 mmol); afforded **11h** as a yellow oil (984.4 mg, 97.5%). ^1^H NMR (DMSO-d_6_, 600 MHz): *δ* 7.15–7.13 (1H, d, *J* = 11.74 Hz); 7.08–7.04 (2H, m); 5.27–5.25 (1H, t, *J* = 5.76 Hz); 4.46–4.45 (2H, d,* J* = 5.76 Hz); 3.80 (3H, s). ^13^C NMR (DMSO-d_6_, 600 MHz): *δ* 152.6, 151.0, 146.3, 146.2, 136.1, 136.0, 122.8, 114.5–114.4, 113.7, 62.5, 56.2.

(3,4,5-trifluorophenyl)methanol **(11i)**. Synthesized using the general reduction procedure with the following quantities: 3,4,5-trifluorobenzaldehyde (2.00 g, 12.49 mmol); NaBH_4_ (708.9 mg, 18.74 mmol); afforded **11i** as a yellow oil (1.93 g, 95.1%). ^1^H NMR (DMSO-d_6_, 600 MHz): *δ* 7.20 (2H, s); 5.50–5.48 (1H, t, *J* = 5.77 Hz); 4.47–4.46 (2H, d, *J* = 5.20 Hz). ^13^C NMR (DMSO-d_6_, 600 MHz): *δ* 151.4–151.3, 149.7–149.6, 140.6, 138.6–138.4, 136.9–136.7, 110.7–110.6, 61.7.

(3,5-dibromo-4-(2-(diethylamino)ethoxy)phenyl)methanol **(11l)**. Synthesized using the general phenol alkylation procedure with the following quantities: 3,5-dibromo-4-hydroxybenzaldehyde (1.00 g, 3.57 mmol); K_2_CO_3_ (1.97 g, 14.29 mmol); 2-chloro-N,N-diethylethan-1-amine hydrochloride (614.8 mg, 3.57 mmol); afforded 3,5-dibromo-4-(2-(diethylamino)ethoxy)benzaldehyde as a yellow oil (413.4 mg, 30.6%). ^1^H NMR (CDCl_3_, 600 MHz): *δ* 9.84 (1H, s); 8.02 (2H, s); 4.17–4.15 (2H, t, *J* = 6.56 Hz); 3.03–3.01 (2H, t, *J* = 6.40 Hz); 2.70–2.66 (4H, q, *J* = 7.11 Hz); 1.09–1.07 (6H, t, *J* = 7.22 Hz). ^13^C NMR (CDCl_3_, 600 MHz): *δ* 188.4, 158.4, 134.0, 133.9, 119.4, 71.7, 52.1, 47.5, 11.8.

Synthesized using the general reduction procedure with the following quantities: 3,5-dibromo-4-(2-(diethylamino)ethoxy)benzaldehyde (413.4 mg, 1.09 mmol); NaBH_4_ (61.9 mg, 1.64 mmol); afforded **11l** as a yellow oil (360.3 mg, 86.7%). ^1^H NMR (CDCl_3_ DMSO-d_6_, 600 MHz): *δ* 7.56 (2H, s); 5.40 (1H, b); 4.44 (2H, s); 3.97–3.95 (2H, t, *J* = 6.39 Hz); 2.88–2.86 (2H, t, *J* = 6.70 Hz); 2.59–2.56 (4H, q, *J* = 7.31 Hz); 0.99–0.96 (6H, t, *J* = 7.07 Hz). ^13^C NMR (CDCl_3_ DMSO-d_6_, 600 MHz): *δ* 151.5, 142.4, 130.9, 117.7, 71.9, 61.5, 52.0, 47.5, 12.3.

General Chlorination Procedure: The appropriate benzyl alcohol (1.0 eq) was dissolved in DCM and cooled to 0 °C. Pyridine (1.3 eq) was added, followed by the addition of SOCl_2_ (1.7 eq), and allowed to warm to room temperature over 16 h. The reaction mixture was quenched with saturated NaHCO_3_, extracted with DCM, washed with DI, brine, dried over anhydrous Na_2_SO_4_, and concentrated *in vacuo* to afford the corresponding crude benzyl chloride which was immediately used in the next reaction.

5-(chloromethyl)-1,3-diiodo-2-methoxybenzene **(12a)**. Synthesized using the general chlorination procedure with the following quantities: **11a** (1.62 g, 4.15 mmol); pyridine (368.1 µL, 4.57 mmol); SOCl_2_ (512.3 µL, 7.06 mmol); afforded **12a** as a yellow oil (1.37 g, 80.6%).

4-(chloromethyl)-2-iodo-1-methoxybenzene **(12b)**. Synthesized using the general chlorination procedure with the following quantities: **11b** (750.0 mg, 2.84 mmol); pyridine (297.4 µL, 3.69 mmol); SOCl_2_ (350.2 µL, 4.83 mmol); afforded **12b** as a yellow oil (crude, Th 802.4 mg).

1,3-dibromo-5-(chloromethyl)-2-methoxybenzene **(12c)**. Synthesized using the general chlorination procedure with the following quantities: **11c** (2.50 g, 8.45 mmol); pyridine (748.5 µL, 9.29 mmol); SOCl_2_ (1.04 ml, 14.36 mmol); afforded **12c** as a yellow oil (crude, Th 2.66 g).

2-bromo-4-(chloromethyl)-1-methoxybenzene **(12d)**. Synthesized using the general chlorination procedure with the following quantities: **11d** (1.95 g, 8.98 mmol); pyridine (974.5 µL, 12.10 mmol); SOCl_2_ (1.15 ml, 15.82 mmol); afforded **12d** as a yellow oil (crude, Th 2.19 g).

1,3-dichloro-5-(chloromethyl)-2-methoxybenzene **(12e)**. Synthesized using the general chlorination procedure with the following quantities: **11e** (639.4 mg, 3.09 mmol); pyridine (323.4 µL, 4.01 mmol); SOCl_2_ (380.8 µL, 5.25 mmol); afforded **12e** as a yellow oil (crude, Th 696.4 mg).

2-chloro-4-(chloromethyl)-1-methoxybenzene **(12f)**. Synthesized using the general chlorination procedure with the following quantities: **11f** (740.2 mg, 4.29 mmol); pyridine (449.1 µL, 5.57 mmol); SOCl_2_ (528.8 µL, 7.29 mmol); afforded **12f** as a yellow oil (crude, Th 819.29 mg).

5-(chloromethyl)-1,3-difluoro-2-methoxybenzene** (12g)**. Synthesized using the general chlorination procedure with the following quantities: **11g** (461.1 mg, 2.69 mmol); pyridine (281.5 µL, 3.49 mmol); SOCl_2_ (331.5 µL, 4.57 mmol); afforded **12g** as a yellow oil (crude, Th 517.7 mg).

4-(chloromethyl)-2-fluoro-1-methoxybenzene **(12h)**. Synthesized using the general chlorination procedure with the following quantities: **11h** (984.4 mg, 6.30 mmol); pyridine (660.1 µL, 8.20 mmol); SOCl_2_ (777.4 µL, 10.72 mmol); afforded **12h** as a yellow oil (crude, Th 1.10 g).

5-(chloromethyl)-1,2,3-trifluorobenzene **(12i)**. Synthesized using the general chlorination procedure with the following quantities: **11i** (1.50 g, 9.25 mmol); pyridine (819.9 µL, 10.18 mmol); SOCl_2_ (1.14 µL, 15.73 mmol); afforded **12i** as a yellow oil (crude, Th 1.67 g).

5-(bromomethyl)-1,2,3-trimethoxybenzene **(12k)**. (3,4,5-Trimethoxyphenyl)methanol (2.03 ml, 12.61 mmol) and PPh_3_ (4.96 g, 18.92 mmol) were dissolved in DCM (100 ml) and cooled to 0 °C. CBr_4_ (8.37 g, 25.22 mmol) was added to the reaction mixture and allowed to warm to room temperature over 16 h. The reaction mixture was quenched with dH_2_O (100 ml) and extracted with DCM (2 × 75 ml). The combined organic extracts were washed with brine (75 ml), dried over anhydrous Mg_2_SO_4_, filtered through a pad of celite, concentrated *in vacuo*, and purified by flash chromatography (SiO_2_, Hex/EA;0–30%) to afford **12k** as a white solid (2.40 g, 72.9%).

2-(2,6-dibromo-4-(chloromethyl)phenoxy)-N,N-diethylethan-1-amine hydrochloride **(12l)**. **11l** (415.6 mg, 1.09 mmol) was dissolved in anhydrous THF (10 ml) and cooled to 0 °C. SOCl_2_ (118.7 µL, 1.64 mmol) was added dropwise and stirred for 2 h. The reaction mixture was diluted with diethyl ether (DE, 20 ml) and cooled to −10 °C with no stirring, resulting in an oily precipitate. The residual solution was decanted off affording **12i** as a clear oil (crude, Th 474.5 mg).

General *N*-Alkylation Procedure: Isatin (1.0 eq) was dissolved in DMF, then K_2_CO_3_ (2.0 eq) and KI (1.0 eq) were added and stirred at room temperature. To the reaction mixture the appropriate benzyl halide (1.15–2.7 eq) was added and stirred for 16 h. The reaction mixture was quenched with 1N HCl (2.0 eq) and extracted with EA. The combined organic extracts were washed with DI, brine, dried over anhydrous Na_2_SO_4_, and concentrated *in vacuo*. The residue was recrystallized from EA/Hex to afford the corresponding *N*-alkylated derivative.

1-(3,5-diiodo-4-methoxybenzyl)indoline-2,3-dione **(8a)**. Synthesized using the general *N*-alkylation procedure with the following quantities: **12a** (682.8 mg, 1.67 mmol); isatin (205.0 mg, 1.39 mmol); K_2_CO_3_ (385.1 mg, 2.79 mmol); KI (231.3 mg, 1.39 mmol); afforded **8a** as a red solid (356.8 mg, 49.3%). ^1^H NMR (DMSO-d_6_, 600 MHz): *δ* 7.93 (2H, s); 7.60–7.56 (2H, m); 7.13–7.10 (1H, m); 6.98–6.96 (1H, d, *J* = 8.06 Hz); 4.81 (2H, s); 3.71 (3H, s). ^13^C NMR (DMSO-d_6_, 600 MHz): *δ* 183.2, 159.0, 158.1, 150.3, 138.7, 138.1, 136.2, 124.8, 123.7, 118.5, 111.2, 92.0, 60.6, 41.3. Measured purity at 254 nm: 97.6%; 280 nm: 98.2%. HRMS: calculated for (C_16_H_11_I_2_NO_3_): 518.8828, found 519.8903 (M + H^+^).

1-(3-iodo-4-methoxybenzyl)indoline-2,3-dione **(8b)**. Synthesized using the general *N*-alkylation procedure with the following quantities: **12b** (802.4 mg, 2.84 mmol); isatin (300.0 mg, 2.04 mmol); K_2_CO_3_ (563.59 mg, 4.08 mmol); KI (338.5 mg, 2.04 mmol); afforded **8b** as a red solid (437.3 mg, 54.5%). ^1^H NMR (DMSO-d_6_, 600 MHz): *δ* 7.86 (1H, d, *J* = 2.28 Hz); 7.59–7.55 (2H, m); 7.44–7.43 (1H, dd); 7.12–7.09 (1H, t, *J* = 8.06 Hz); 6.99–6.94 (2H, m); 4.81 (2H, s); 3.79 (3H, s). ^13^C NMR (DMSO-d_6_, 600 MHz): *δ* 183.5, 158.8, 157.6, 150.6, 138.3, 138.2, 130.0, 129.5, 124.8, 123.7, 118.3, 111.9, 111.4, 86.8, 56.8, 42.0. Measured purity at 254 nm: 99.7%; 280 nm: 99.5%. HRMS: calculated for (C_16_H_12_INO_3_): 392.9862, found 393.9939 (M + H^+^).

1-(3,5-dibromo-4-methoxybenzyl)indoline-2,3-dione **(8c)**. Synthesized using the general *N*-alkylation procedure with the following quantities: **12c** (2.66 g, 8.45 mmol); isatin (500.0 mg, 3.4 mmol); K_2_CO_3_ (939.3 mg, 6.80 mmol); KI (564.1 mg, 3.4 mmol); afforded **8c** as a red solid (708.7 mg, 49.2%). ^1^H NMR (DMSO-d_6_, 600 MHz): *δ* 7.79 (2H, s); 7.60–7.56 (2H, m); 7.13–7.11 (1H, m); 6.97–6.96 (1H, d,* J* = 7.94 Hz); 4.86 (2H, s); 3.76 (3H, s). ^13^C NMR (DMSO-d_6_, 600 MHz): *δ* 183.2, 159.0, 153.1, 150.3, 138.1, 135.5, 132.1, 124.8, 123.7, 118.6, 118.0, 111.2, 60.8, 41.7. Measured purity at 254 nm: 98.1%; 280 nm: 96.7%. HRMS: calculated for (C_16_H_11_Br_2_NO_3_): 422.9106, found 423.9189 (M + H^+^).

1-(3-bromo-4-methoxybenzyl)indoline-2,3-dione **(8d)**. Synthesized using the general *N*-alkylation procedure with the following quantities: **12d** (2.19 g, 9.31 mmol); isatin (500.0 mg, 3.40 mmol); K_2_CO_3_ (939.3 mg, 6.80 mmol); KI (564.1 mg, 3.40 mmol); afforded **8d** as a red solid (478.2 mg, 40.5%). ^1^H NMR (CDCl_3_, 600 MHz): *δ* 7.63–7.62 (1H, m); 7.54–7.50 (2H, m); 7.27–7.25 (2H, m); 7.13–7.10 (1H, m); 6.87–8.86 (1H, d, *J* = 8.20 Hz); 6.79–6.78 (1H, d, *J* = 7.52 Hz); 4.85 (2H, s); 3.88 (3H, s). ^13^C NMR (CDCl_3_, 600 MHz): *δ* 183.0, 158.2, 155.8, 150.4, 138.3, 132.4, 127.9, 127.8, 125.7, 125.5, 124.0, 121.5, 117.7, 112.2, 112.1, 110.8, 56.3, 42.9. Measured purity at 254 nm: 99.3%; 280 nm: 98.0%. HRMS: calculated for (C_16_H_12_BrNO_3_): 345.0001, found 346.0075 (M + H^+^).

1-(3,5-dichloro-4-methoxybenzyl)indoline-2,3-dione **(8e)**. Synthesized using the general *N*-alkylation procedure with the following quantities: **12e** (696.4 mg, 3.09 mmol); isatin (300.0 mg, 2.04 mmol); K_2_CO_3_ (563.6 mg, 4.08 mmol); KI (338.5 mg, 2.04 mmol); afforded **8e** as a red solid (312.9 mg, 45.6%). ^1^H NMR (DMSO-d_6_, 600 MHz): *δ* 7.63 (2H, s); 7.59–7.56 (2H, m); 7.13–7.11 (1H, td); 6.97–6.95 (1H, d, *J* = 7.91 Hz); 4.87 (1H, s); 3.79 (3H, s). ^13^C NMR (DMSO-d_6_, 600 MHz): *δ* 183.2, 159.0, 151.2, 150.2, 138.1, 134.5, 128.8, 128.5, 124.8, 123.7, 118.5, 111.2, 61.0, 42.0. Measured purity at 254 nm: 98.7%; 280 nm: 97.4%. HRMS: calculated for (C_16_H_11_Cl_2_NO_3_): 335.0116, found 336.0196 (M + H^+^).

1-(3-chloro-4-methoxybenzyl)indoline-2,3-dione **(8f)**. Synthesized using the general *N*-alkylation procedure with the following quantities: **12f** (819.29 mg, 4.29 mmol); isatin (400.0 mg, 2.72 mmol); K_2_CO_3_ (751.5 mg, 5.44 mmol); KI (451.3 mg, 2.72 mmol); afforded **8f** as a red solid (326.7 mg, 39.8%). ^1^H NMR (CDCl_3_, 600 MHz): *δ* 7.62–7.61 (1H, m); 7.52–7.49 (1H, m); 7.36–7.35 (1H, d,* J* = 2.32 Hz); 7.21–7.20 (1H, dd); 7.12–7.09 (1H, td); 6.89–6.88 (1H, d,* J* = 8.35 Hz); 6.78–6.77 (1H, d,* J* = 7.99 Hz); 4.84 (2H, s); 3.88 (3H, s). ^13^C NMR (CDCl_3_, 600 MHz): *δ* 183.0, 158.2, 154.9, 150.4, 138.3, 129.4, 127.5, 127.0, 125.5, 124.0, 123.0, 117.7, 112.4, 110.8, 56.2, 43.0. Measured purity at 254 nm: 99.8%; 280 nm: 99.7%. HRMS: calculated for (C_16_H_12_ClNO_3_): 301.0506, found 302.0590 (M + H^+^).

1-(3,5-difluoro-4-methoxybenzyl)indoline-2,3-dione **(8g)**. Synthesized using the general *N*-alkylation procedure with the following quantities: **12g** (517.7 mg, 2.69 mmol); isatin (300.0 mg, 2.04 mmol); K_2_CO_3_ (563.6 mg, 4.08 mmol); KI (338.5 mg, 2.04 mmol); afforded **8g** as a red solid (211.2 mg, 34.2%). ^1^H NMR (DMSO-d_6_, 600 MHz): *δ* 7.59–7.56 (2H, m); 7.31–7.27 (2H, m); 7.13–7.10 (1H, td); 6.93–6.92 (1H, m); 4.85 (2H, s); 3.88 (3H, s). ^13^C NMR (DMSO-d_6_, 600 MHz): *δ* 183.2, 158.9, 156.3, 154.7–154.6, 150.3, 138.1, 135.4–135.2, 132.2–132.1, 124.8, 123.7, 118.5, 112.0–111.9, 111.2, 62.2, 42.2. Measured purity at 254 nm: 97.6%; 280 nm: 97.9%. HRMS: calculated for (C_16_H_11_F_2_NO_3_): 303.0707, found 304.0782 (M + H^+^).

1-(3-fluoro-4-methoxybenzyl)indoline-2,3-dione **(8h)**. Synthesized using the general *N*-alkylation procedure with the following quantities: **12h** (1.10 g, 6.30 mmol); isatin (500.0 mg, 3.40 mmol); K_2_CO_3_ (939.3 mg, 6.80 mmol); KI (564.1 mg, 3.40 mmol); afforded **8h** as a red solid (608.6 mg, 62.8%). ^1^H NMR (DMSO-d_6_, 600 MHz): *δ* 7.48–7.55 (2H, m); 7.34–7.31 (1H, dd); 7.23–7.21 (1H, dd); 7.13–7.09 (2H, m); 6.96–6.95 (1H, d, *J* = 7.49 Hz); 4.83 (2H, s); 3.80 (3H, s). ^13^C NMR (DMSO-d_6_, 600 MHz): *δ* 182.4, 158.8, 152.6, 151.0, 150.5, 146.9, 138.2, 128.7, 124.8–123.7, 118.3, 115.7–115.5, 114.3, 111.4, 56.4, 42.4. Measured purity at 254 nm: 99.9%; 280 nm: 99.7%. HRMS: calculated for (C_16_H_12_FNO_3_): 285.0801, found 286.0883 (M + H^+^).

1-(3,4,5-trifluorobenzyl)indoline-2,3-dione **(8i)**. Synthesized using the general *N*-alkylation procedure with the following quantities: **12i** (1.67 g, 9.25 mmol); isatin (500.0 mg, 3.40 mmol); K_2_CO_3_ (939.3 mg, 6.80 mmol); KI (564.1 mg, 3.40 mmol); afforded **8i** as a red solid (318.1 mg, 32.1%). ^1^H NMR (CDCl_3_, 600 MHz): *δ* 7.66–7.65 (1H, m); 7.56–7.53 (1H, m); 7.17–7.14 (1H, td); 6.99–6.96 (2H, t,* J* = 7.10 Hz); 6.74–6.73 (1H, d,* J* = 7.81 Hz); 4.86 (2H, s). ^13^C NMR (CDCl_3_, 600 MHz): *δ* 182.4, 158.1, 152.4, 152.3, 150.7, 150.6, 149.8, 140.4, 138.7, 138.5, 130.9, 130.8, 125.8, 124.4, 117.7, 111.6, 111.5, 110.4, 42.9. Measured purity at 254 nm: 99.8%; 280 nm: 99.7%. HRMS: calculated for (C_15_H_8_F_3_NO_2_): 291.0507, found 292.0593 (M + H^+^).

1-(3-methoxybenzyl)indoline-2,3-dione **(8j)**. Synthesized using the general *N*-alkylation procedure with the following quantities: **12j** (1.01 ml, 7.48 mmol); isatin (500.0 mg, 3.40 mmol); K_2_CO_3_ (939.3 mg, 6.80 mmol); KI (564.1 mg, 3.40 mmol); afforded **8j** as a red solid (321.7 mg, 35.4%). ^1^H NMR (CDCl_3_, 600 MHz): *δ* 7.62–7.60 (1H, m); 7.49–7.46 (1H, td); 7.27–7.25 (2H, m); 7.10–7.07 (1H, td); 6.92–6.90 (1H, dd); 6.86–6.82 (2H, m); 6.79–6.77 (1H, d,* J* = 7.93 Hz); 4.90 (2H, s); 3.78 (3H, s). ^13^C NMR (CDCl_3_, 600 MHz): *δ* 183.2, 160.1, 158.2, 150.7, 138.3, 136.0, 130.1, 125.4, 123.8, 119.6, 117.6, 113.3, 113.2, 111.0, 55.5, 44.0. Measured purity at 254 nm: 99.9%; 280 nm: 99.9%. HRMS: calculated for (C_16_H_13_NO_3_): 267.0895, found 290.0789 (M + Na^+^).

1-(3,4,5-trimethoxybenzyl)indoline-2,3-dione **(8k)**. Synthesized using the general *N*-alkylation procedure with the following quantities: **12k** (1.02 g, 3.91 mmol); isatin (500.0 mg, 3.40 mmol); K_2_CO_3_ (939.3 mg, 6.80 mmol); KI (564.1 mg, 3.40 mmol); afforded **8k** as a red solid (507.4 mg, 45.7%). ^1^H NMR (CDCl_3_, 600 MHz): *δ* 7.63–762 (1H, dd); 7.53–7.50 (1H, td); 7.12–7.10 (1H, td), 6.83–6.81 (1H, d,* J* = 7.81 Hz); 6.53 (2H, s); 4.85 (2H, s); 3.82 (6H, s); 3.82 (3H, s). ^13^C NMR (CDCl_3_, 600 MHz): *δ* 183.2, 158.3, 153.7, 150.7, 138.3, 137.8, 130.1, 125.5, 124.0, 117.6, 111.0, 104.5, 60.9, 56.2, 44.4. Measured purity at 254 nm: 99.8%; 280 nm: 99.3%. HRMS: calculated for (C_18_H_17_NO_5_): 327.1107, found 328.1201 (M + H^+^).

1-(3,5-dibromo-4-(2-(diethylamino)ethoxy)benzyl)indoline-2,3-dione hydrochloride **(8l)**. Synthesized using the general *N*-alkylation procedure with the following quantities: **12l** (475.5 mg, 1.09 mmol); isatin (160.5 mg, 1.09 mmol); K_2_CO_3_ (452.1 mg, 3.27 mmol); KI (181.0 mg, 1.09 mmol); The crude material was purified by flash chromatography (SiO_2_, DCM/MeOH;0–5%) to afford **8l** as a red wax (45.2 mg, 8.1%). ^1^H NMR (CDCl_3_, 600 MHz): *δ* 7.61–7.65 (1H, dd); 7.57–7.54 (1H, td); 7.48 (2H, s); 7.17–7.14 (1H, td); 6.78–6.77 (1H, d,* J* = 7.49 Hz); 4.83 (2H, s); 4.09–4.07 (2H, t,* J* = 6.40 Hz); 3.01 (2H, s); 2.70 (4H, s); 1.10–1.07 (6H, t,* J* = 6.99 Hz). ^13^C NMR (CDCl_3_, 600 MHz): *δ* 182.6, 158.1, 153.4, 150.0, 138.5, 133.0, 131.5, 125.7, 124.3, 119.0, 117.7, 110.6, 52.0, 47.5, 42.5, 29.7, 11.7. Measured purity by elemental analysis: calculated for (C: 49.44, H: 4.35, N: 5.49, Br: 31.32); found (C: 49.43, H: 4.32, N: 5.49, Br: 31.09). HRMS: calculated for (C_21_H_23_Br_2_N_2_O_3_): 507.9997, found 509.0086 (M + H^+^).

### Supplementary Information

Supplementary Figures.

## Data Availability

Supporting information is available in Supplemental Materials.pdf. The datasets generated during and/or analyzed during the current study are available from the corresponding author on reasonable request.

## Abbreviations

*mAChR*: Muscarinic acetylcholine receptor
*PAM*: Positive allosteric modulator
*NAM*: Negative allosteric modulator
*ASD*: Autism spectrum disorder
*CNS*: Central nervous system
*CHO*: Chinese hamster ovary
*Eqn*: Equation
*GPCR*: G protein-coupled receptor
*AD*: Alzheimer’s disease
*PD*: Parkinson’s disease
*ACh*: Acetylcholine
*AA*: Arachidonic acid
*SAR*: Structure activity relationship
*POB*: Phenoxy benzamine
*BSA*: Bovine serum albumin
*TLC*: Thin layer chromatography
*DMSO*: Dimethyl sulfoxide
*CCl*_*4*_: Carbon tetrachloride
*DMF*: Dimethylformamide
*EA*: Ethyl acetate
*DCM*: Dichloromethane
*Hex*: Hexane
*HRMS*: High-resolution mass spectrum
