# Supplementary material for: Allosteric modulators of M1 muscarinic receptors enhance acetylcholine efficacy and decrease locomotor activity and turning behaviors in zebrafish
Source: Sci Rep. 2024 Jun 28;14:14901. doi: 10.1038/s41598-024-65445-y (PMC11213934; doi:10.1038/s41598-024-65445-y)

Supporting Information

Allosteric Modulators of M_1 Muscarinic Receptors Enhance Acetylcholine Efficacy and Decrease Locomotor Activity and Turning Behaviors in Zebrafish

Corey J. Widman[1], Sestina Ventresca[2], Jillian Dietrich[2], Gwendolynne Elmslie[3], Hazel Smith[1], Gina Kaup[1], Aaron Wesley[2], Madeline Doenecke[2], Frederick E. Williams[2], Isaac T. Schiefer[1,4], John Ellis[3], William S. Messer, Jr.[1,2]

[1]Department of Medicinal and Biological Chemistry, College of Pharmacy and Pharmaceutical Sciences, University of Toledo, Toledo, OH 43614

[2]Department of Pharmacology and Experimental Therapeutics, College of Pharmacy and Pharmaceutical Sciences, University of Toledo, Toledo, OH 43614

[3]Departments of Psychiatry and Pharmacology, College of Medicine, Penn State University, Hershey, PA 17033

[4]Center for Drug Design and Development, College of Pharmacy and Pharmaceutical Sciences, University of Toledo, Toledo, OH 43614

List of Contents

Supplement Figure S1: Correlation data of molecular surface area vs activity.

Supplemental Figure S2: Toxicity of test compounds during a 24-hour exposure represented as the percentage of animals exhibiting an abnormal phenotype. Embryos were observed periodically during exposure for abnormalities and death. An abnormal phenotype consisted of three endpoints: startle capacity, swim position, and morphological abnormalities.

Supplement Figure S3 - S21: Characterization data of novel final compounds.

Area	No ACh	0.1 µM ACh	100 µM ACh
286.38	0.02	1.07	0.74
291.44	0.04	1.17	1.03
302.85	0.08	1.49	1.13
305.65	0.04	1.31	0.87
312.2	0.08	1.81	1.24
297.05	0.03	1.33	0.94
317.65	0.08	2.13	1.28
325.96	0.23	2.15	1.58
338.01	0.24	2.64	1.62

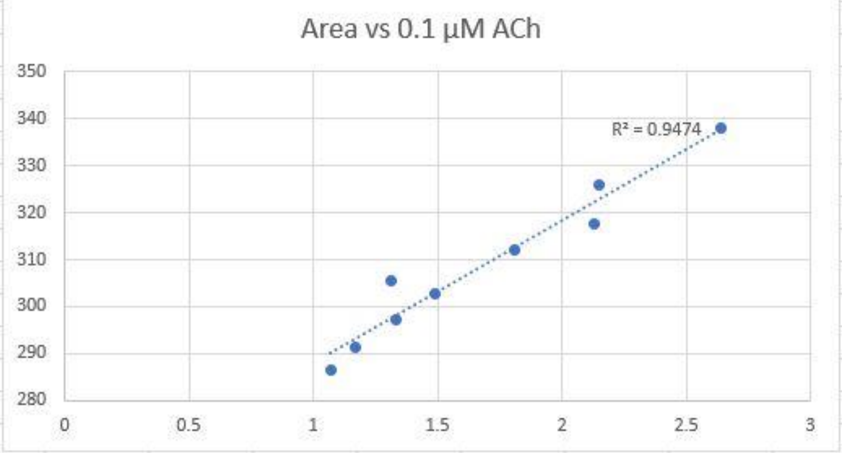

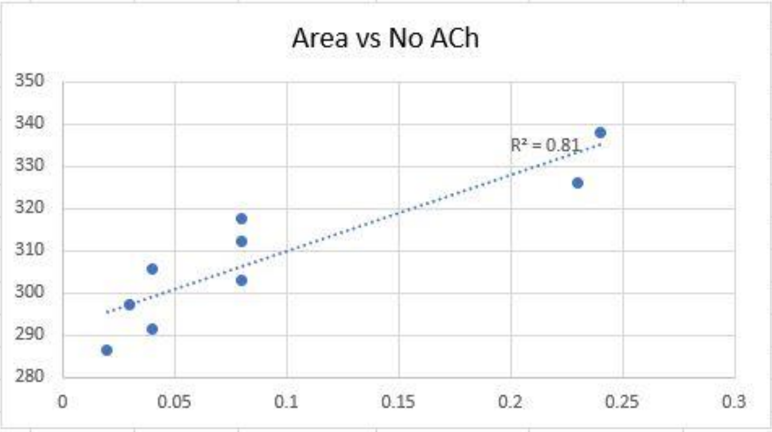

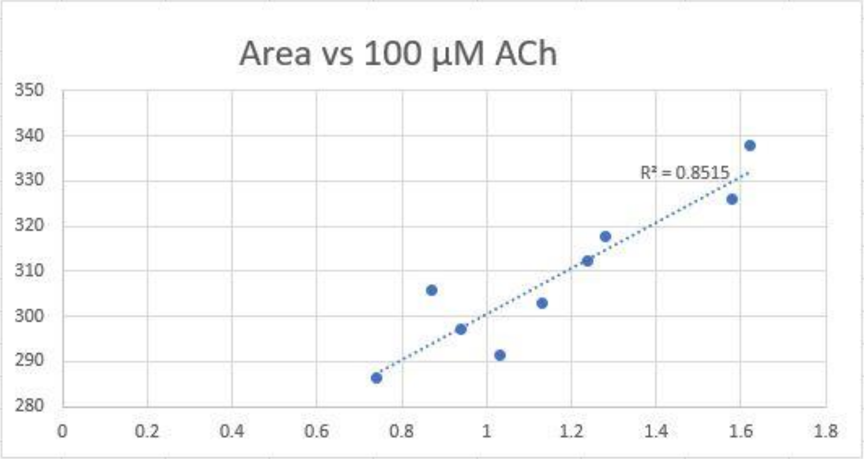

Supplement Figure S1: Correlation data of molecular surface area vs activity

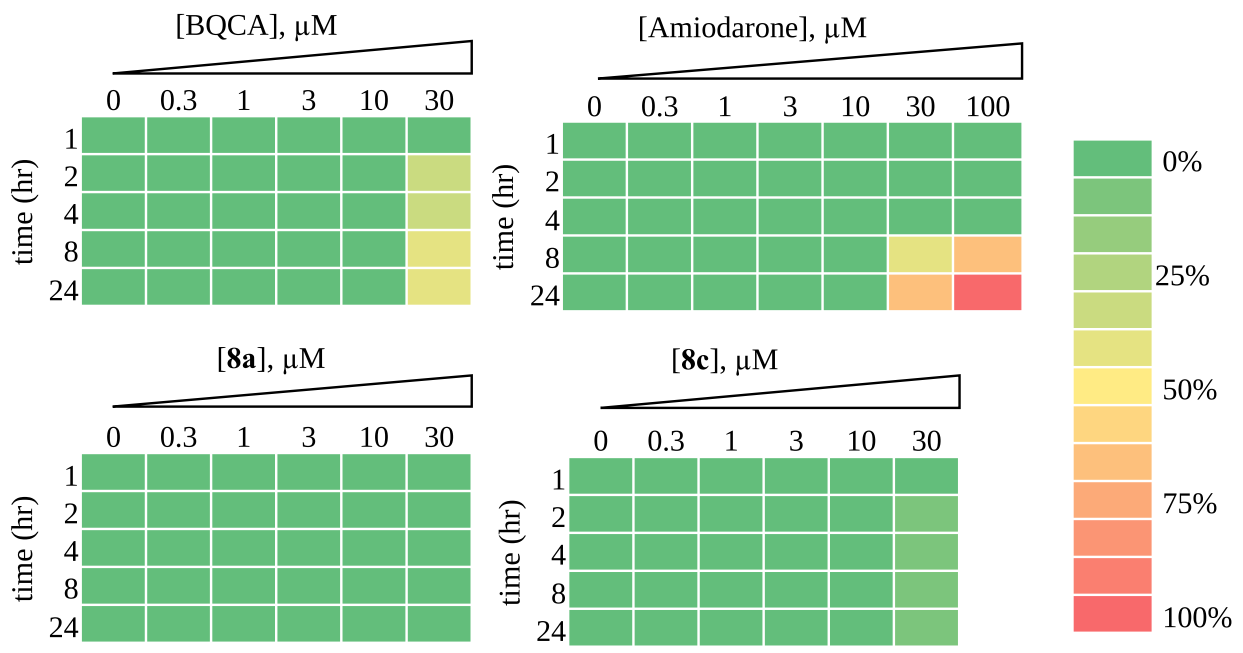

Supplemental Figure S2: Toxicity of test compounds during a 24-hour exposure represented as the percentage of animals exhibiting an abnormal phenotype. Embryos were observed periodically during exposure for abnormalities and death. An abnormal phenotype consisted of three endpoints: startle capacity, swim position, and morphological abnormalities.

Analysis Report

^{1}H NMR

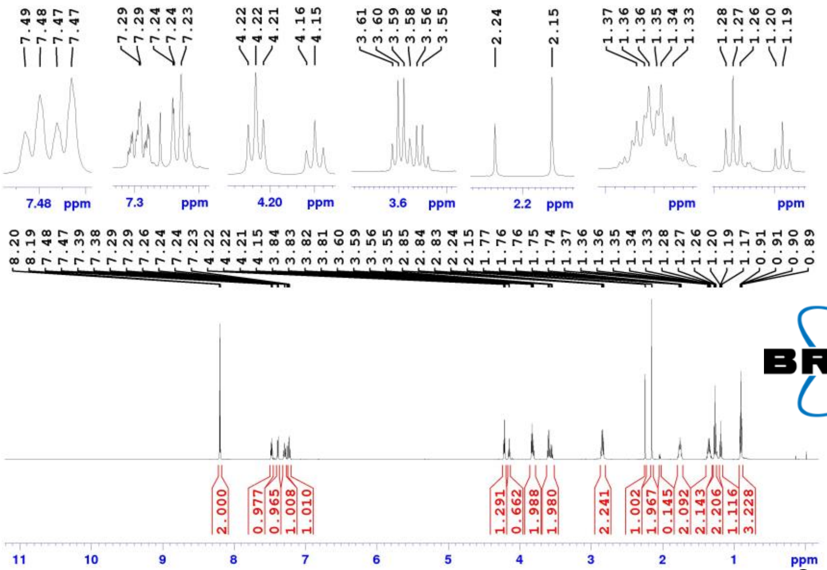

^{13}C APT NMR

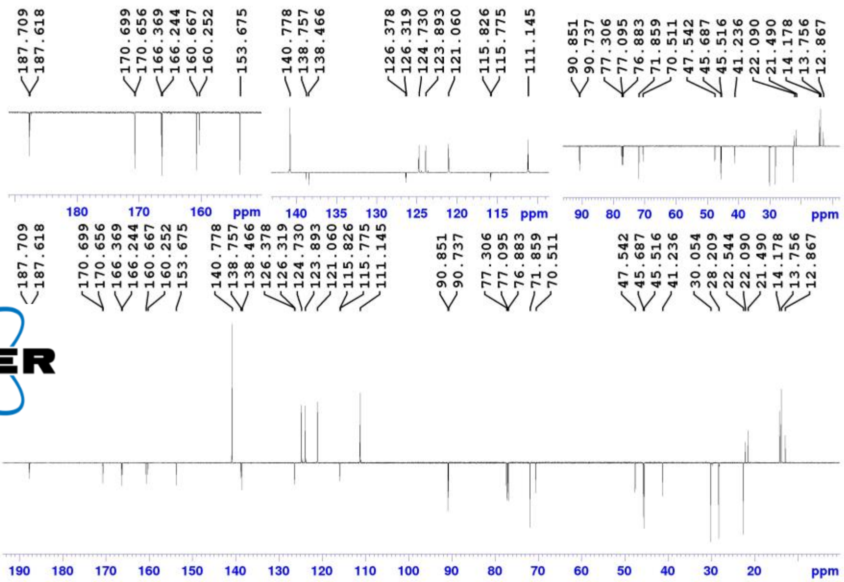

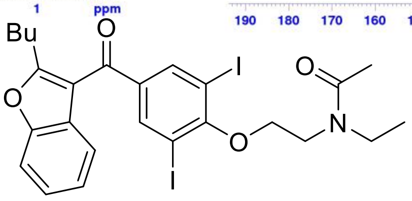

5

HPLC purity (2 wavelength)

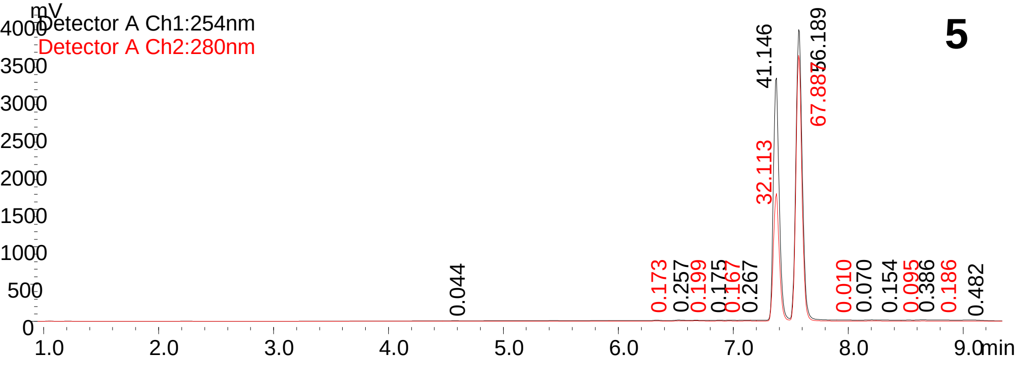

HRMS

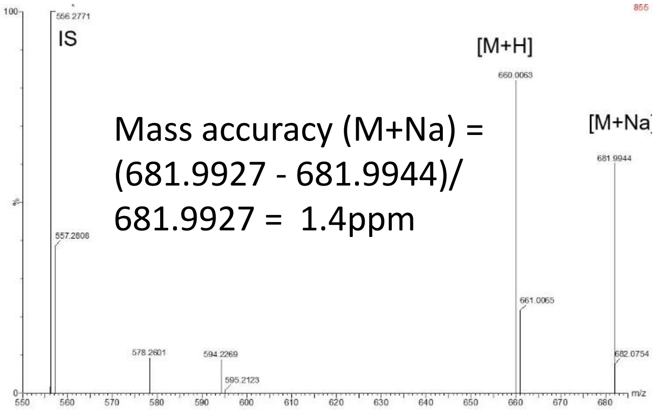

Mass accuracy (M+Na) = (681.9927 - 681.9944)/ 681.9927 = 1.4ppm

Analysis Report

^{1}H NMR

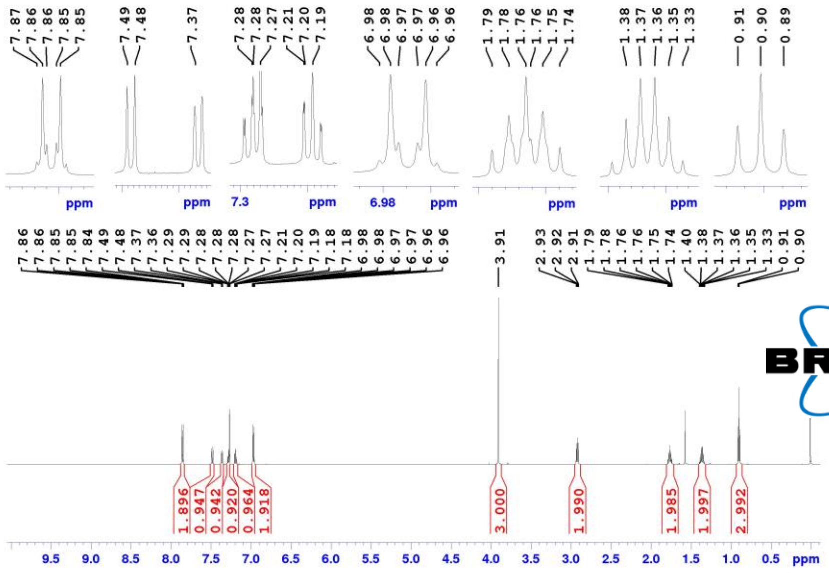

^{13}C APT NMR

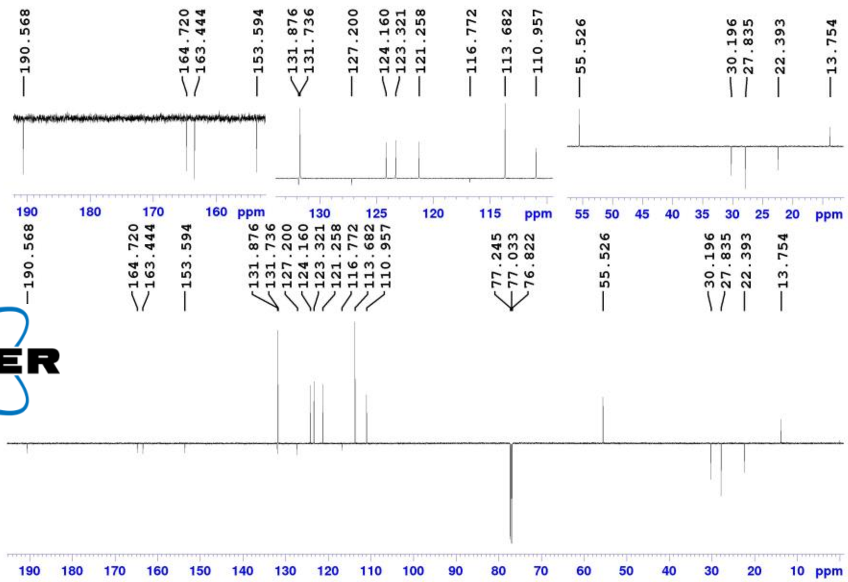

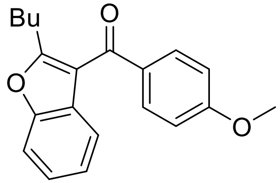

6a

HPLC purity
(2 wavelength)

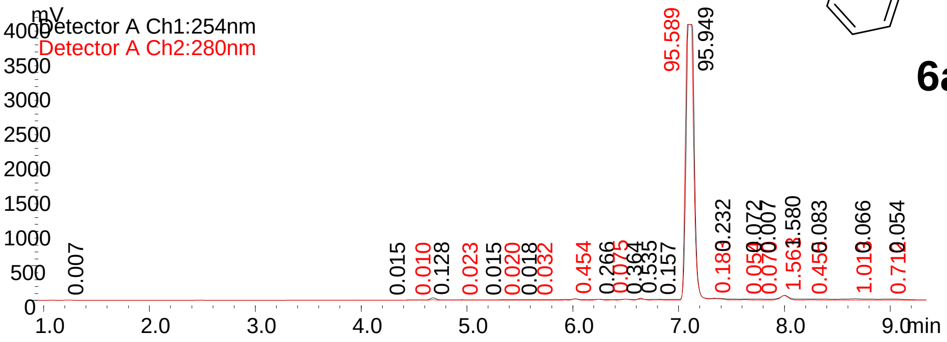

HRMS

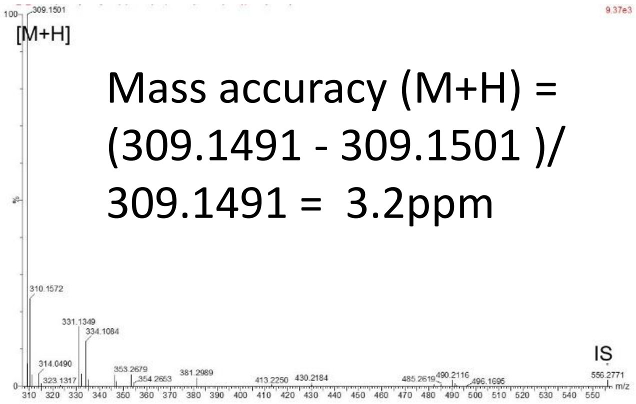

Mass accuracy (M+H) = (309.1491 - 309.1501)/ 309.1491 = 3.2ppm

Analysis Report

^{1}H NMR

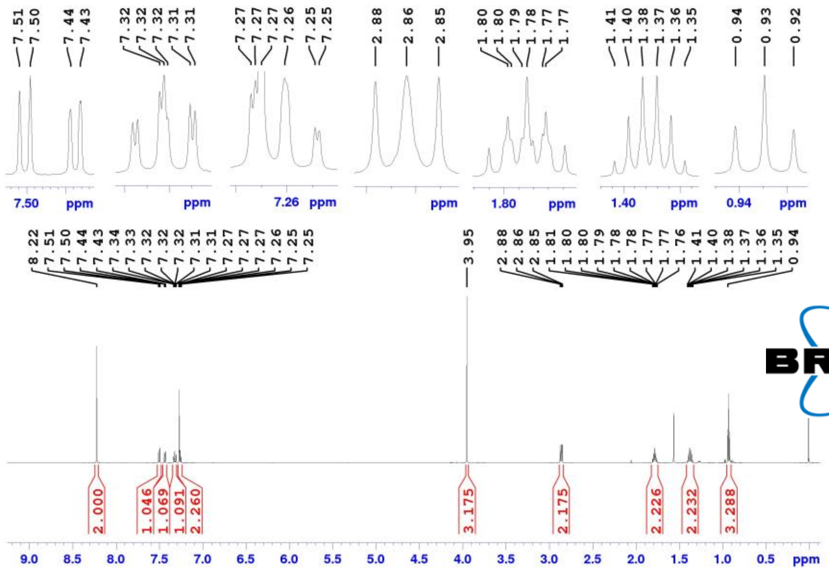

^{13}C APT NMR

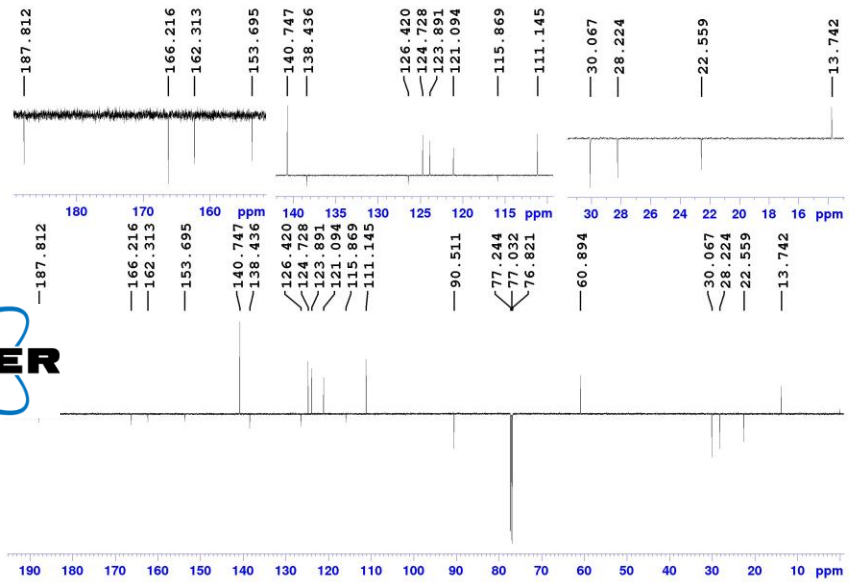

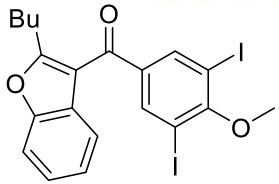

6b

HPLC purity
(2 wavelength)

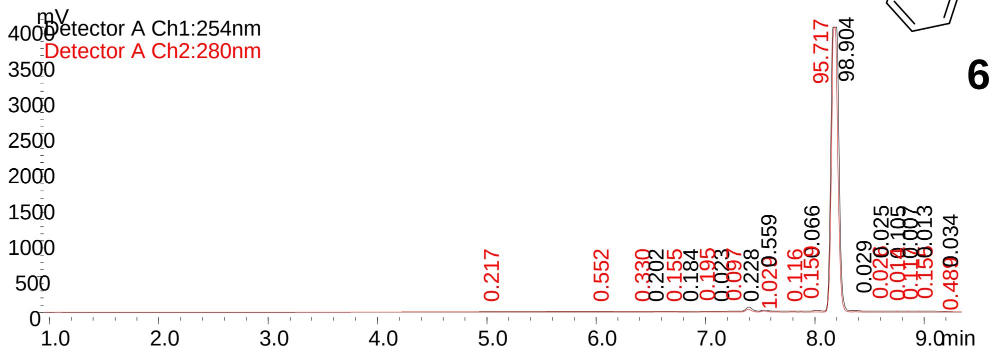

HRMS

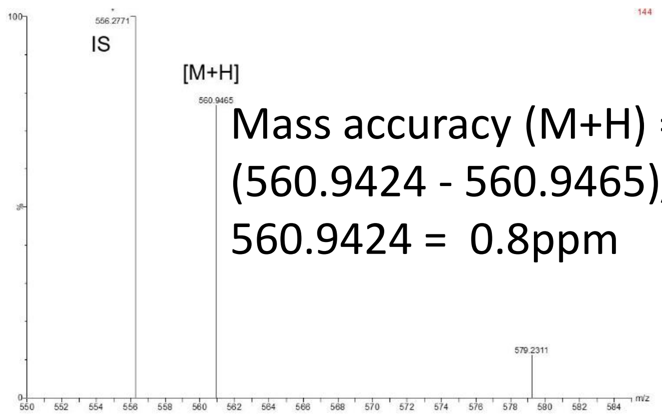

Mass accuracy (M+H) = (560.9424 - 560.9465)/ 560.9424 = 0.8ppm

Analysis Report

^{1}H NMR

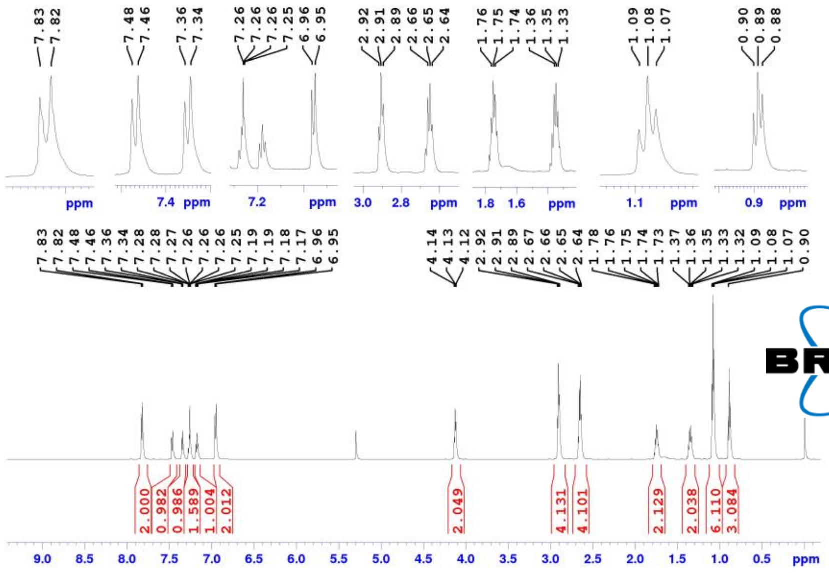

^{13}C APT NMR

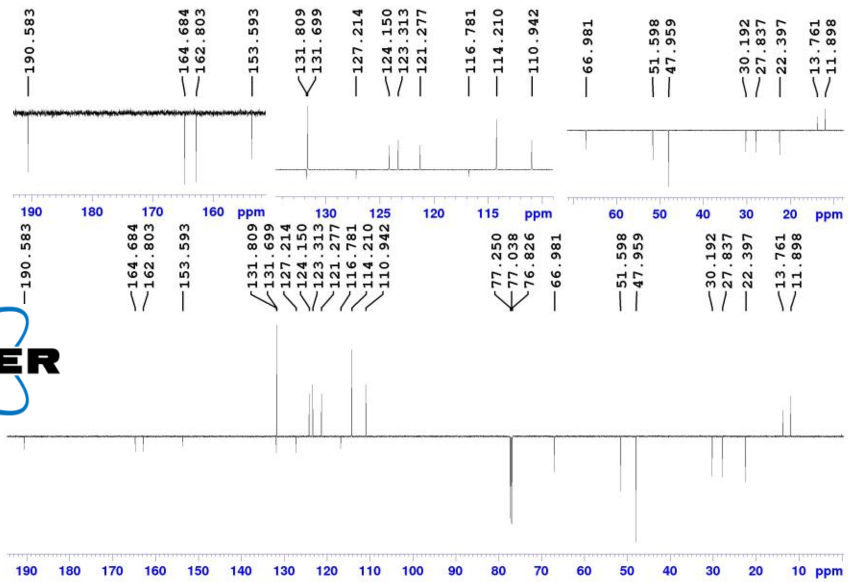

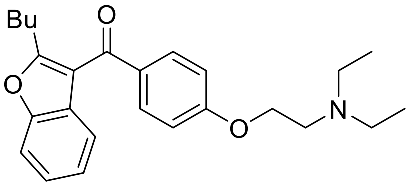

6c

HPLC purity
(2 wavelength)

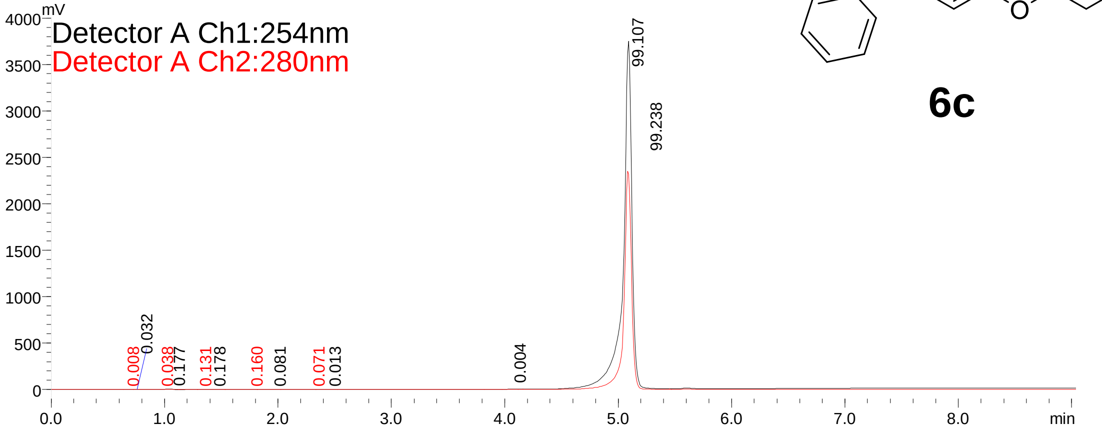

HRMS

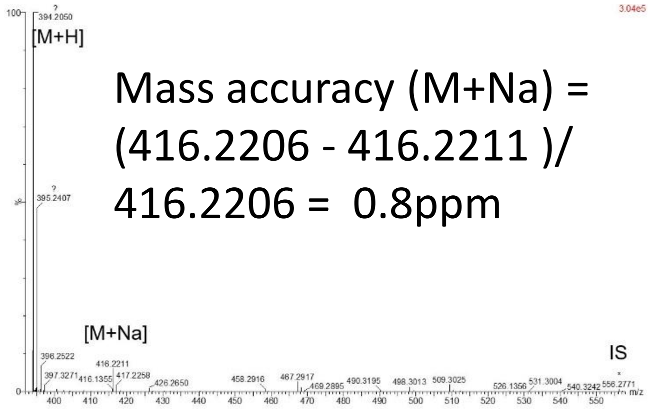

Mass accuracy (M+Na) = (416.2206 - 416.2211)/ 416.2206 = 0.8ppm

Analysis Report

^{1}H NMR

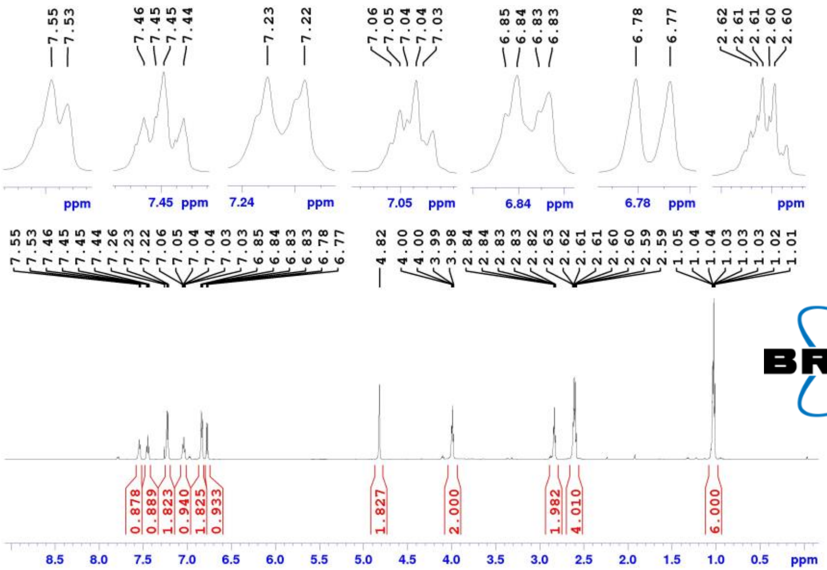

^{13}C APT NMR

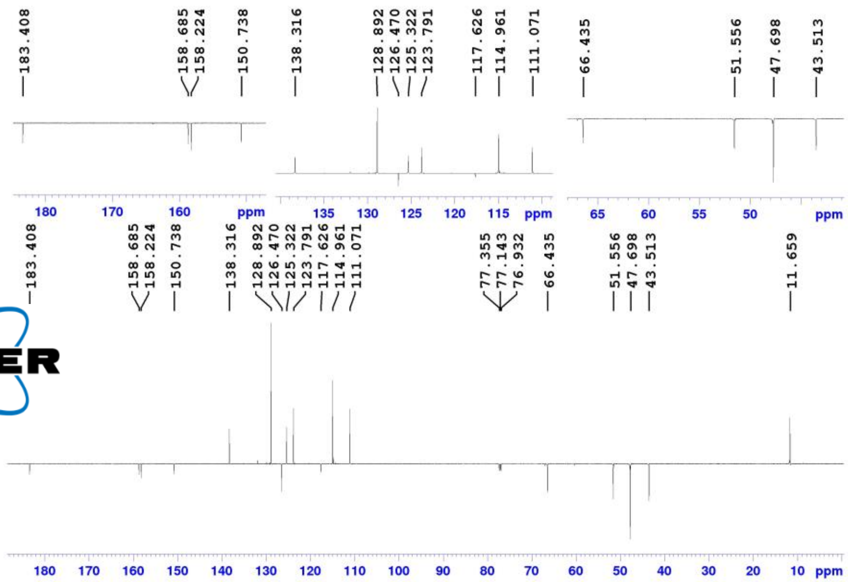

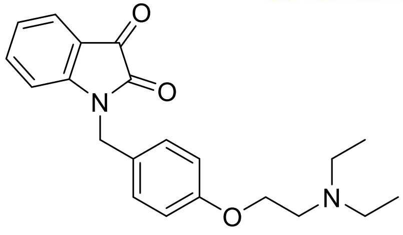

7

Elemental Analysis purity

Element	Theory	Found	
C	71.57	70.99	70.85
H	6.86	6.69	6.67
N	7.95	7.67	7.62

HRMS

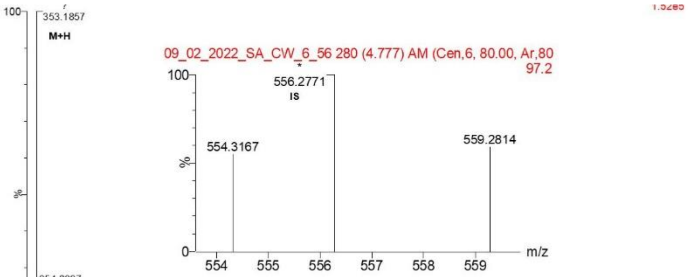

Mass accuracy (M+H) = (353.1865 - 353.1857)/ 353.1865)* 10^{6} = 2.2 ppm

Analysis Report

^{1}H NMR

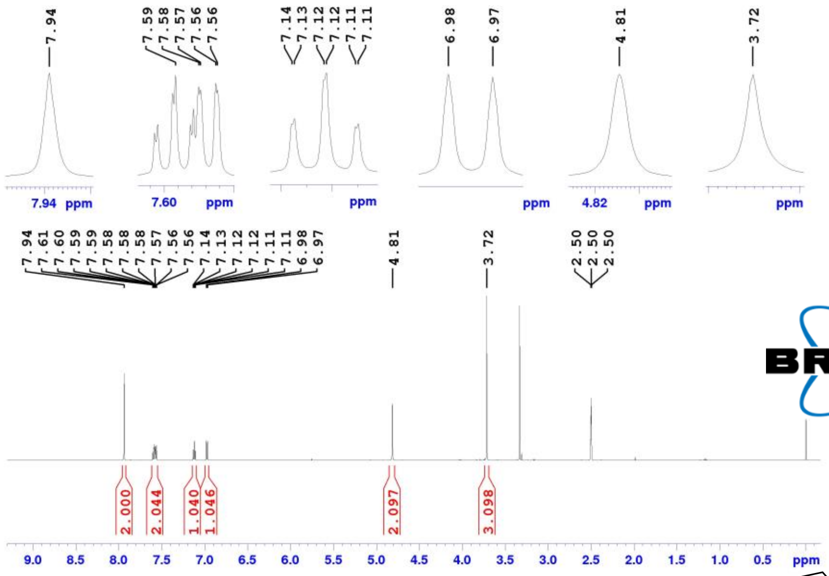

^{13}C APT NMR

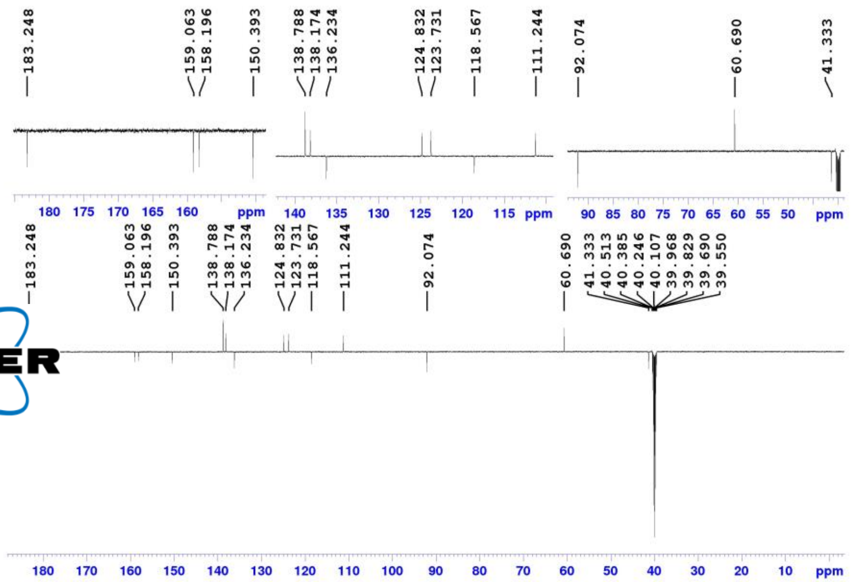

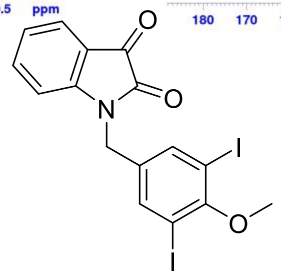

8a

HPLC purity
(2 wavelength)

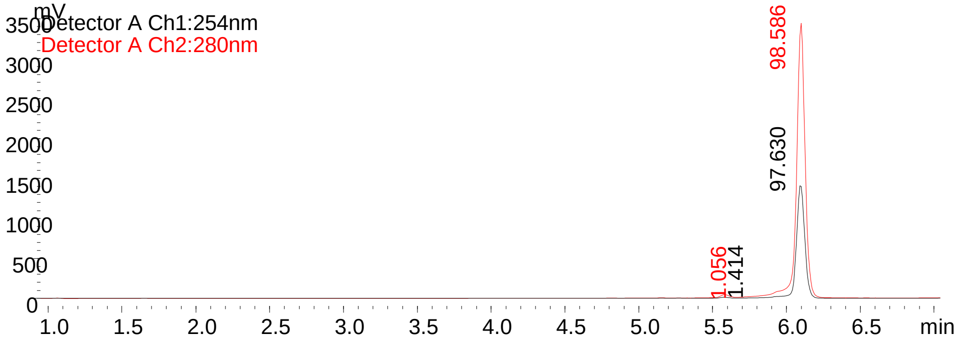

HRMS

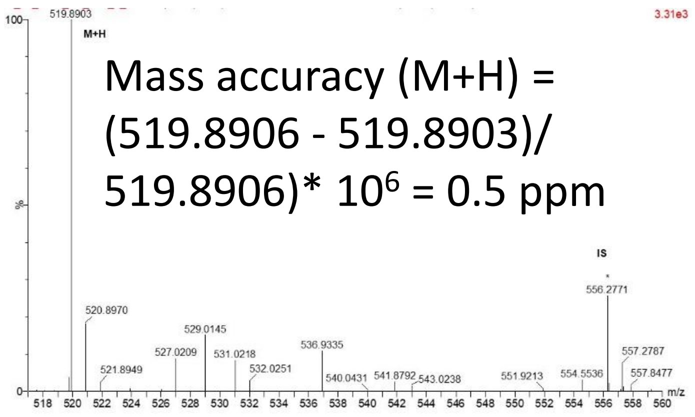

Mass accuracy (M+H) = (519.8906 - 519.8903)/ 519.8906)* 10^{6} = 0.5 ppm

Analysis Report

^{1}H NMR

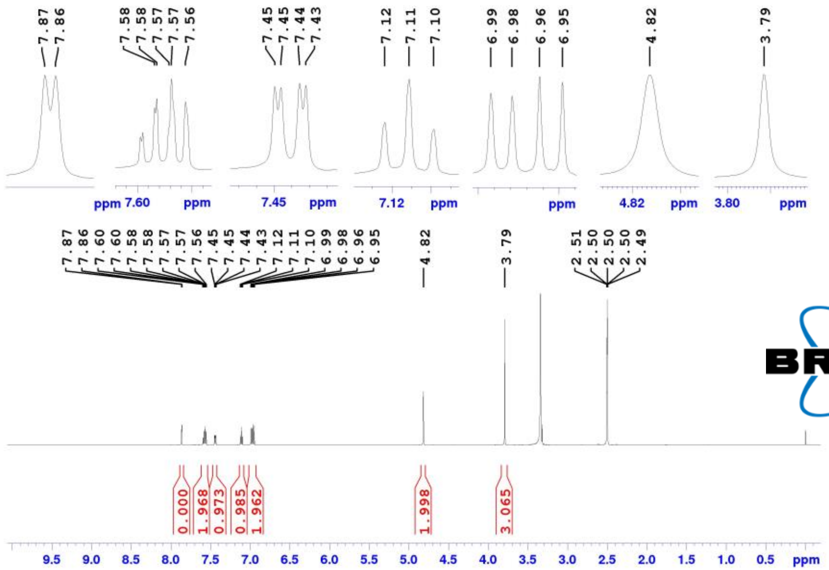

^{13}C APT NMR

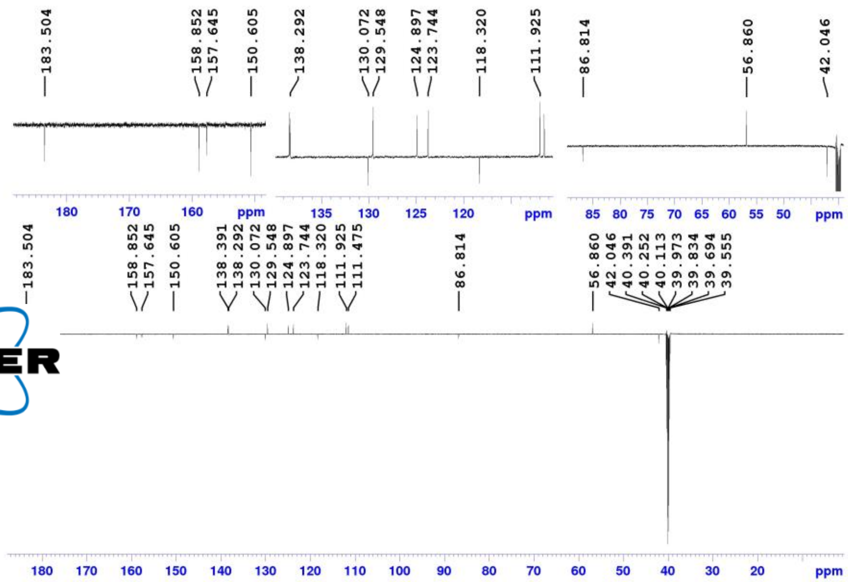

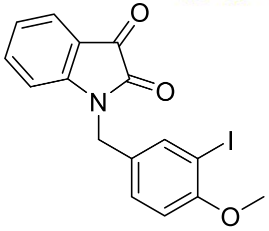

8b

HPLC purity (2 wavelength)

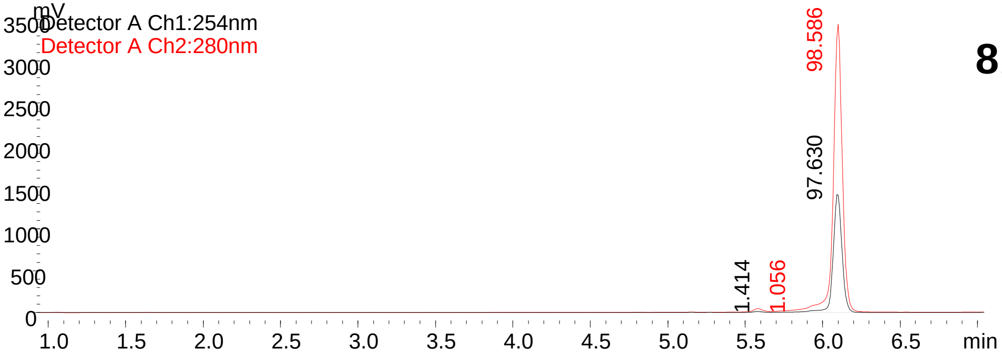

HRMS

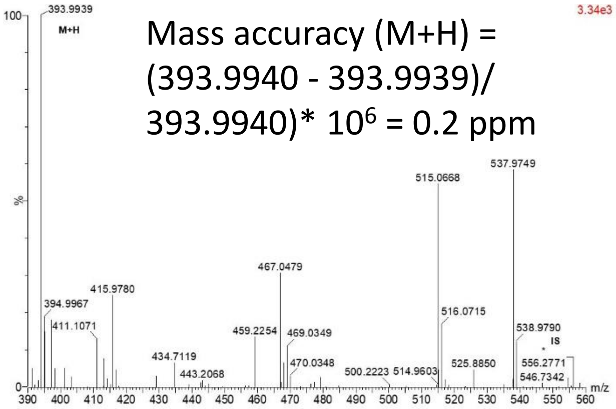

Mass accuracy (M+H) = (393.9940 - 393.9939)/ 393.9940)* 10^6 = 0.2 ppm

Analysis Report

1H NMR

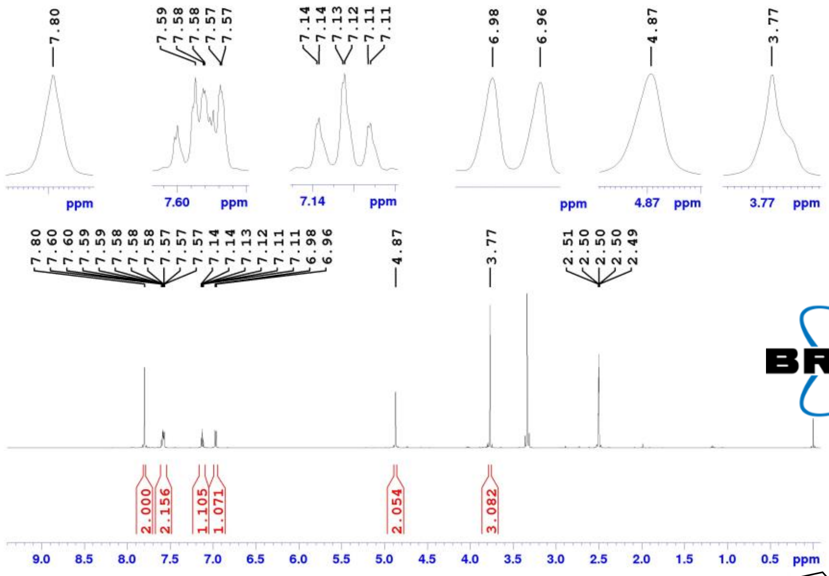

13C APT NMR

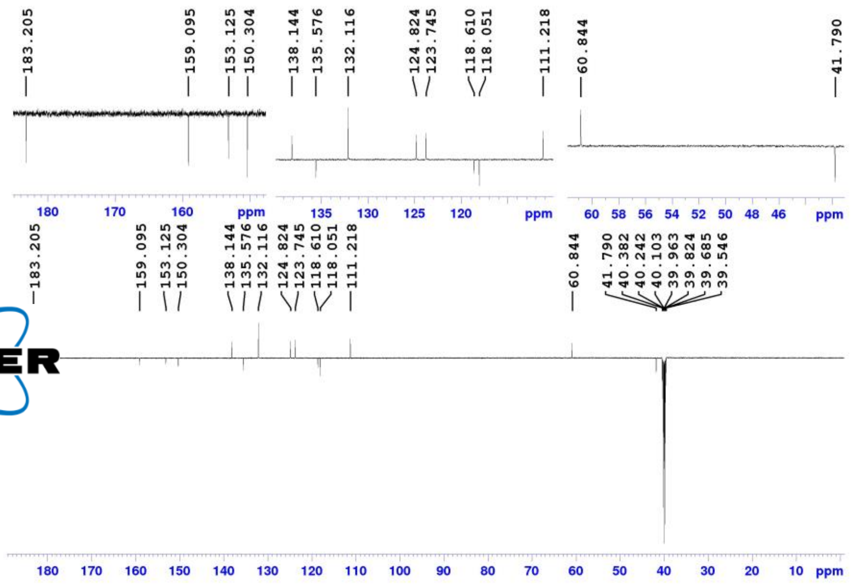

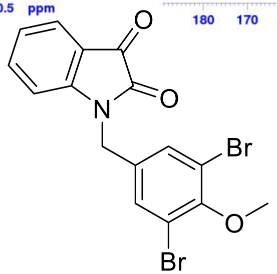

8c

HPLC purity (2 wavelength)

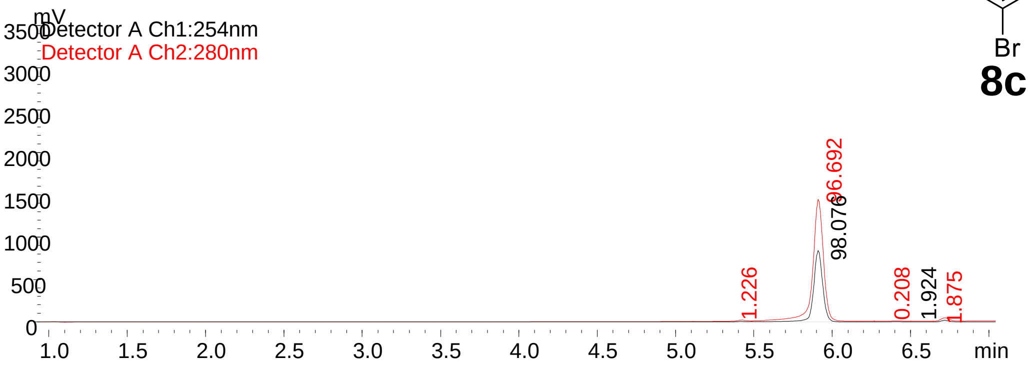

HRMS

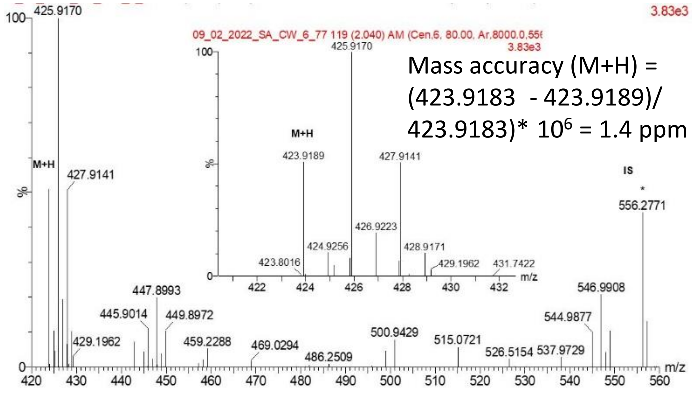

Mass accuracy (M+H) = (423.9183 - 423.9189)/ 423.9183)* 10^6 = 1.4 ppm

Analysis Report

^{1}H NMR

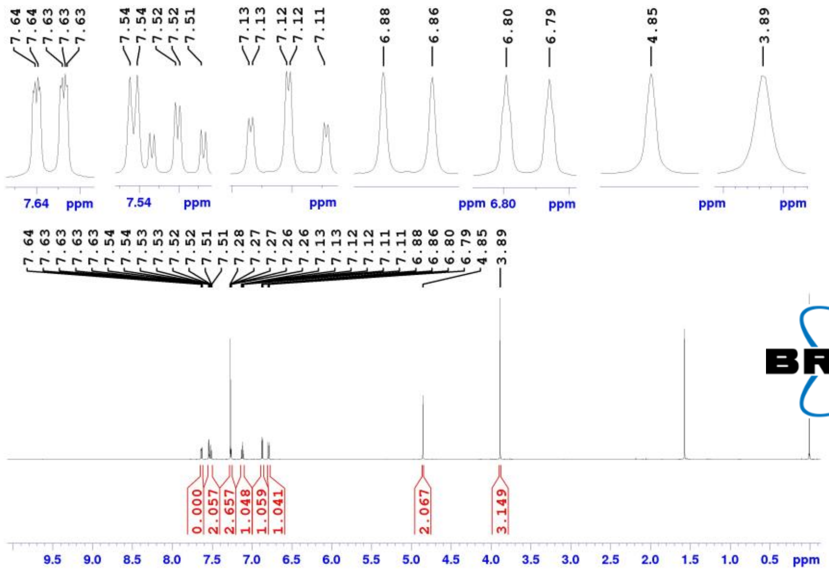

^{13}C APT NMR

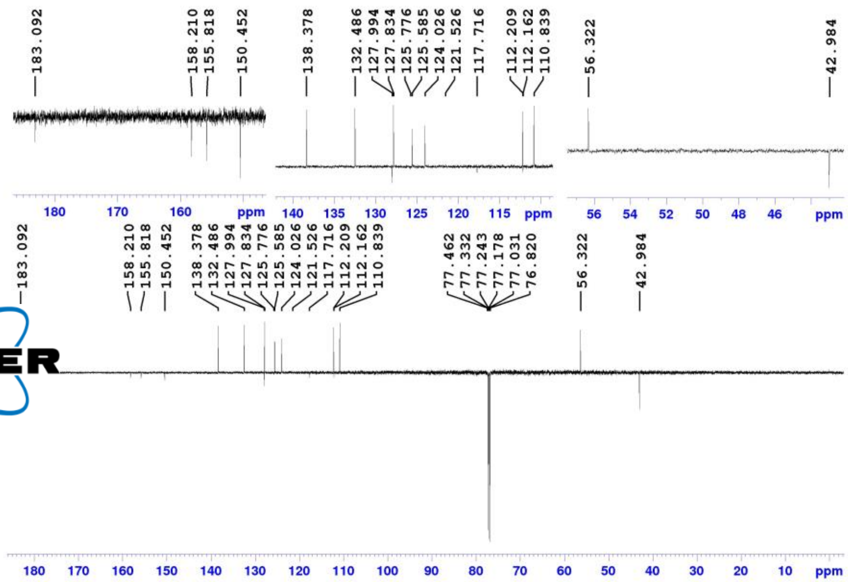

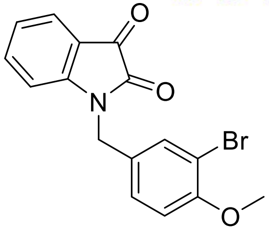

8d

HPLC purity (2 wavelength)

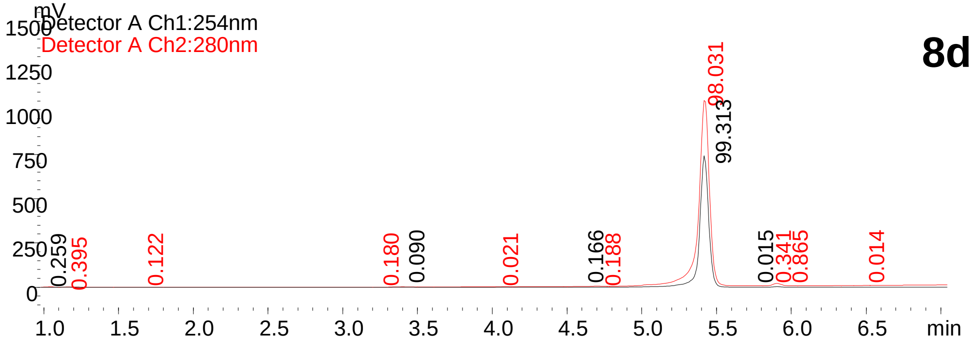

HRMS

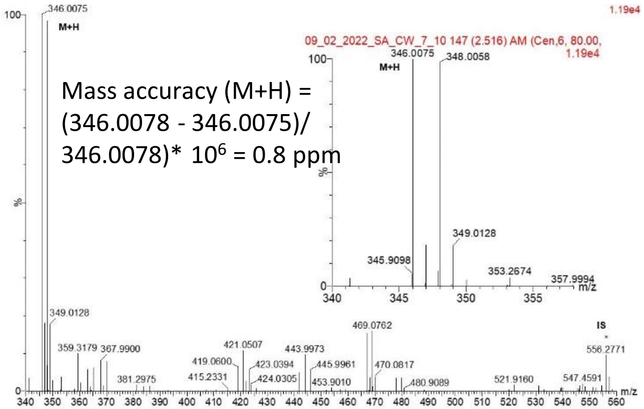

Analysis Report

^{1}H NMR

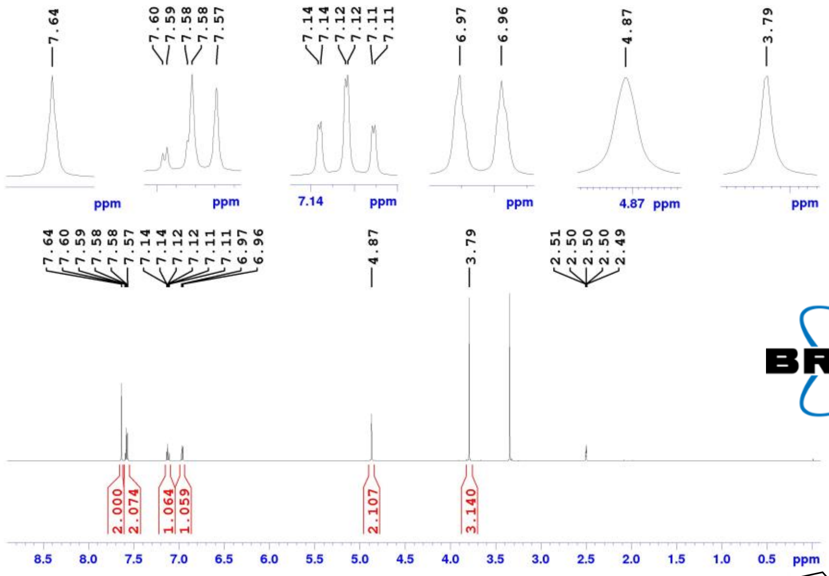

^{13}C APT NMR

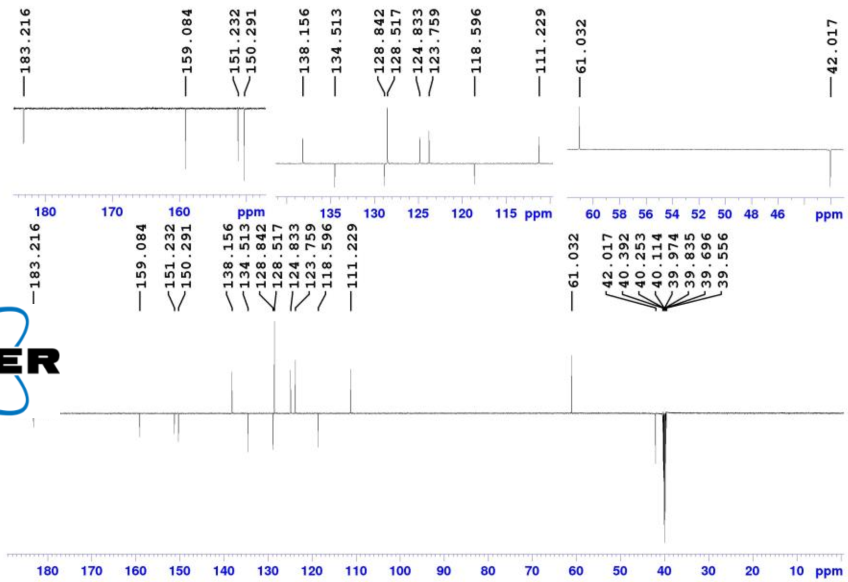

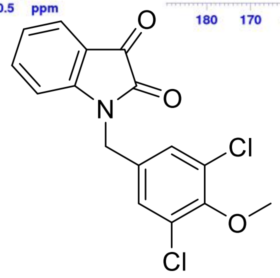

8e

HPLC purity
(2 wavelength)

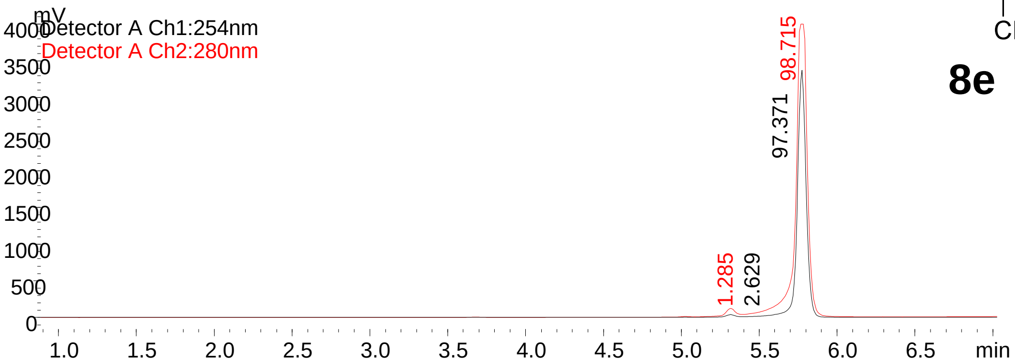

HRMS

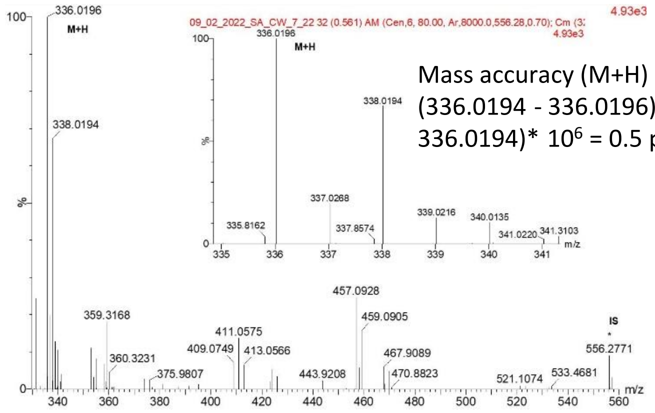

Mass accuracy (M+H) = (336.0194 - 336.0196)/ 336.0194)* 10^6 = 0.5 ppm

Analysis Report

^{1}H NMR

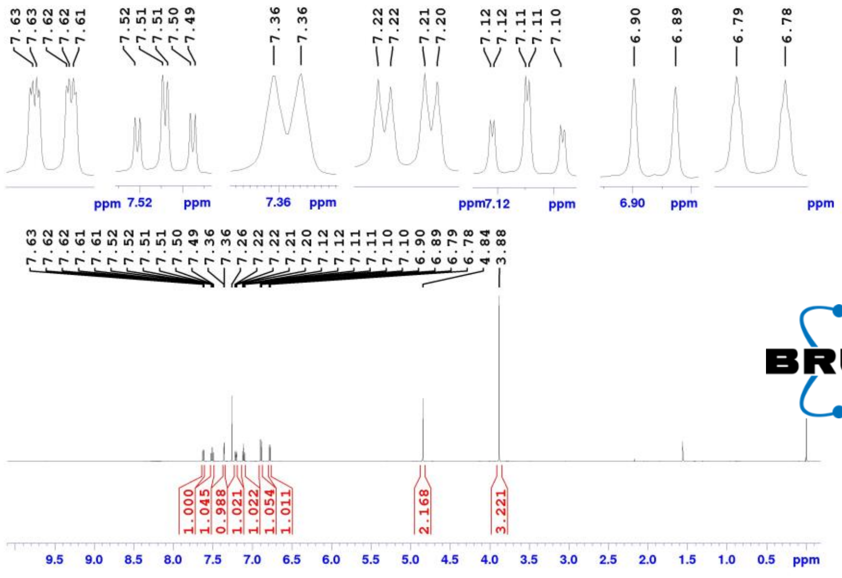

^{13}C APT NMR

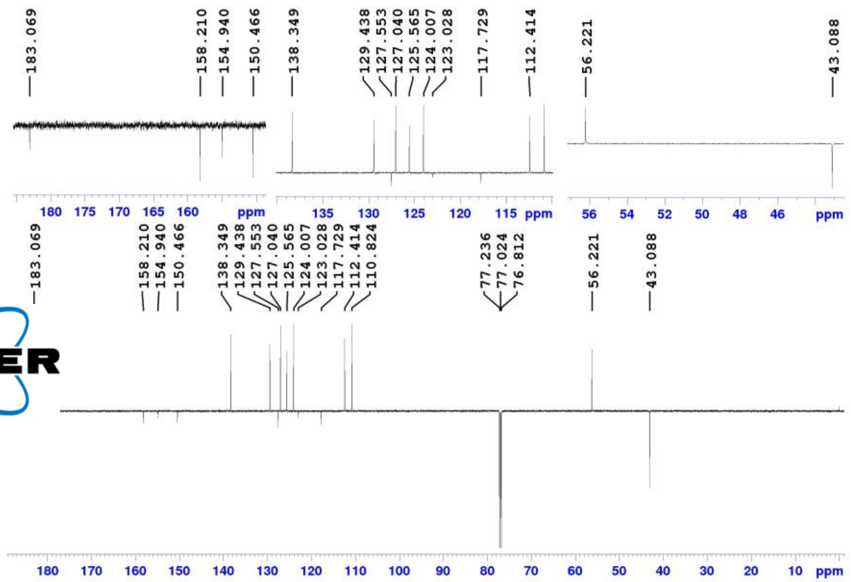

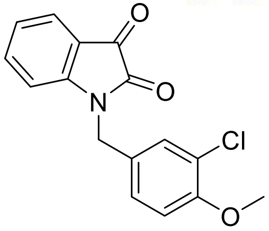

8f

HPLC purity (2 wavelength)

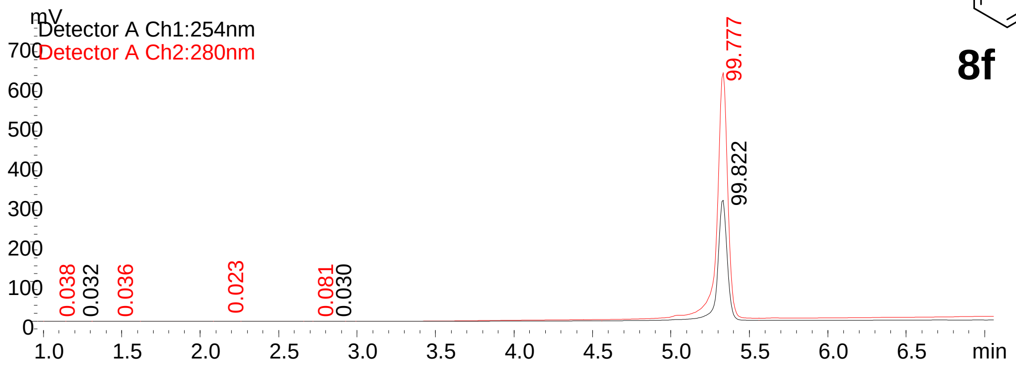

HRMS

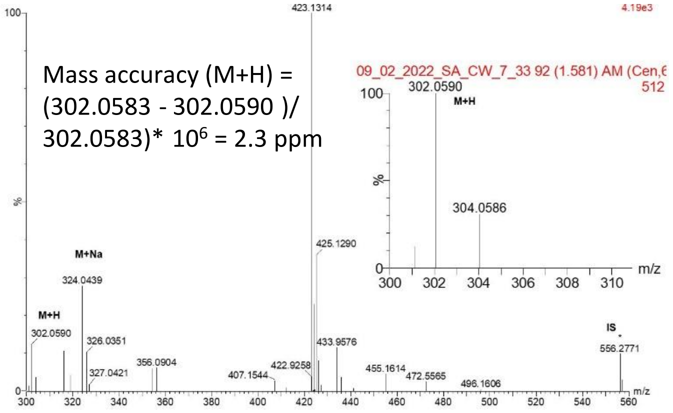

Analysis Report

^{1}H NMR

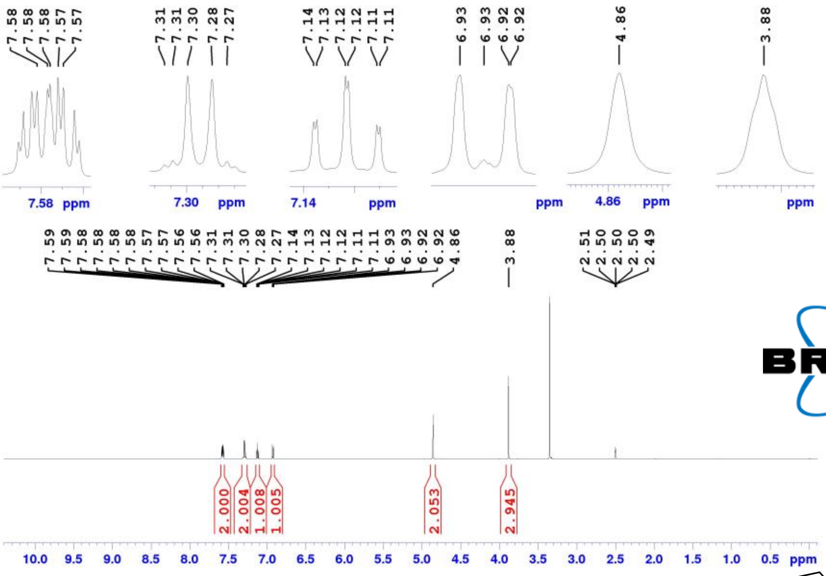

^{13}C APT NMR

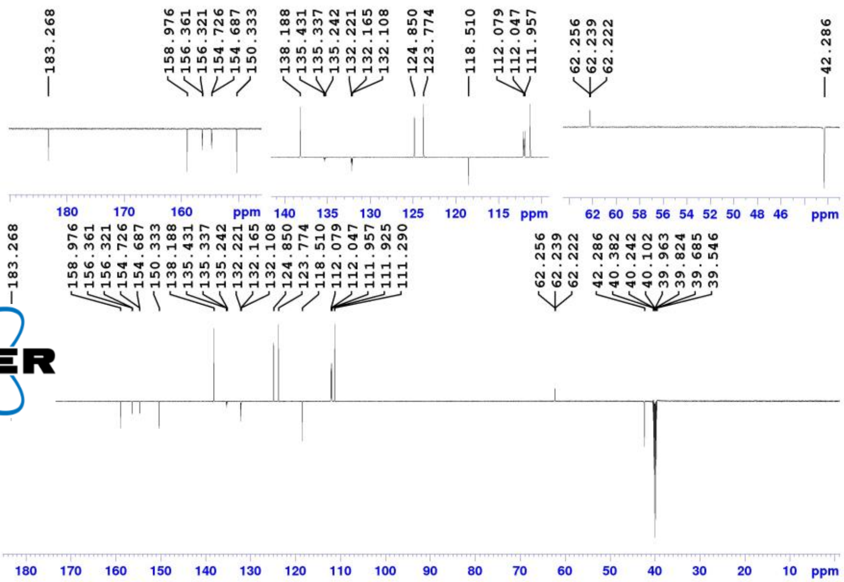

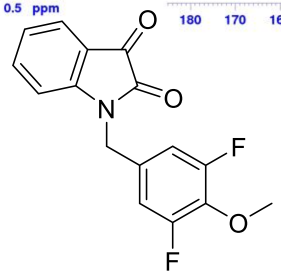

8g

HPLC purity
(2 wavelength)

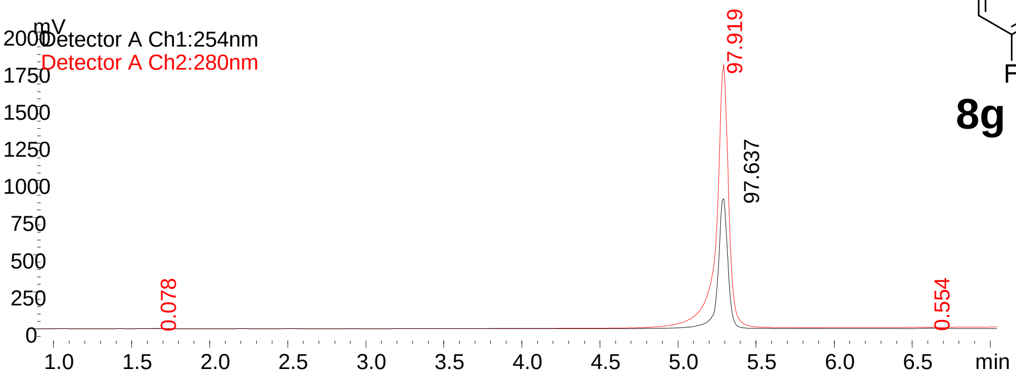

HRMS

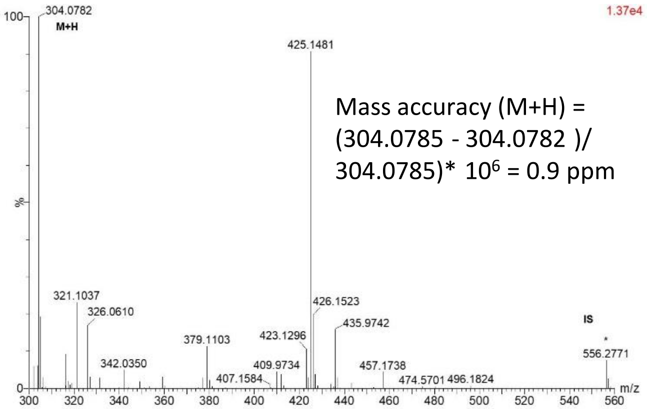

Mass accuracy (M+H) = (304.0785 - 304.0782)/ 304.0785)* 10^6 = 0.9 ppm

Analysis Report

^{1}H NMR

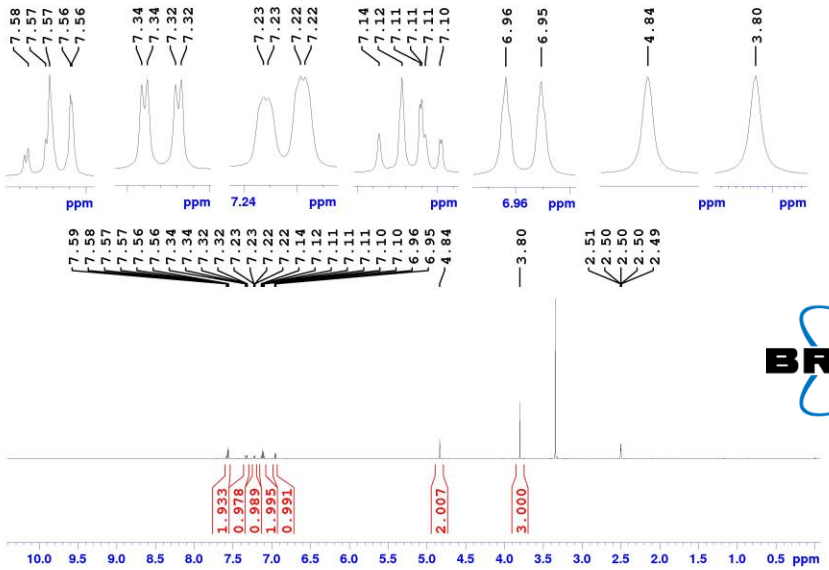

^{13}C APT NMR

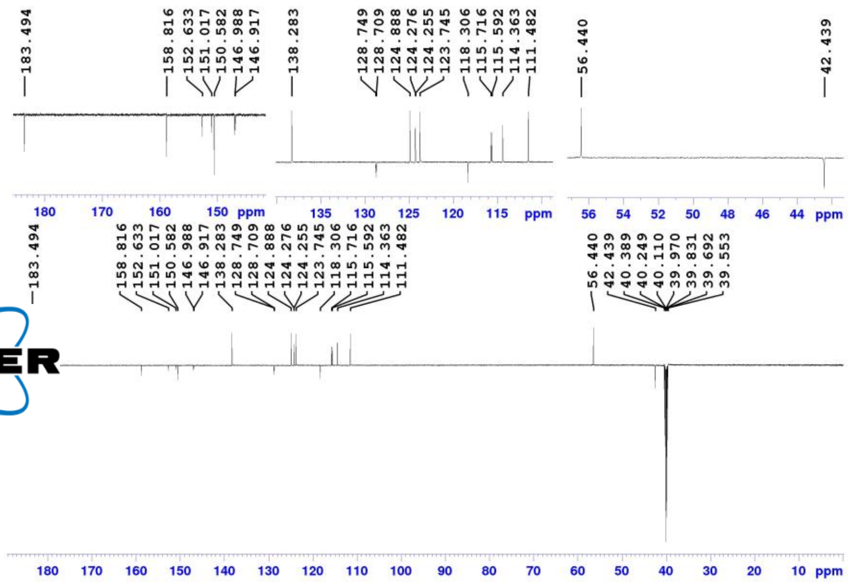

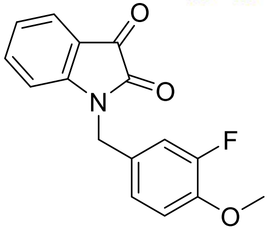

8h

HPLC purity (2 wavelength)

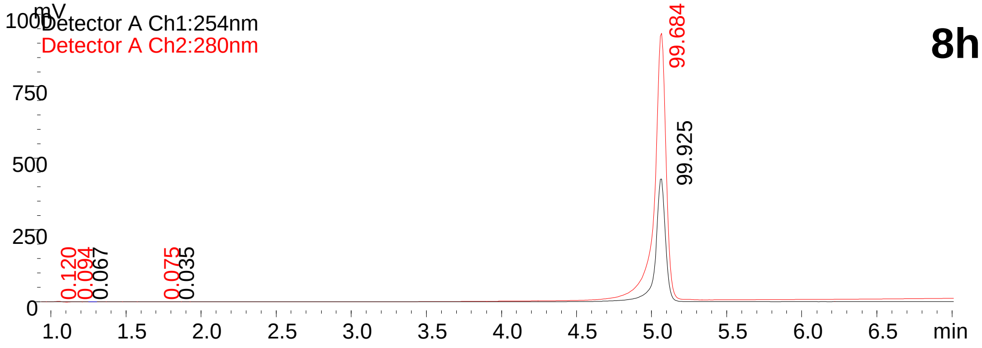

HRMS

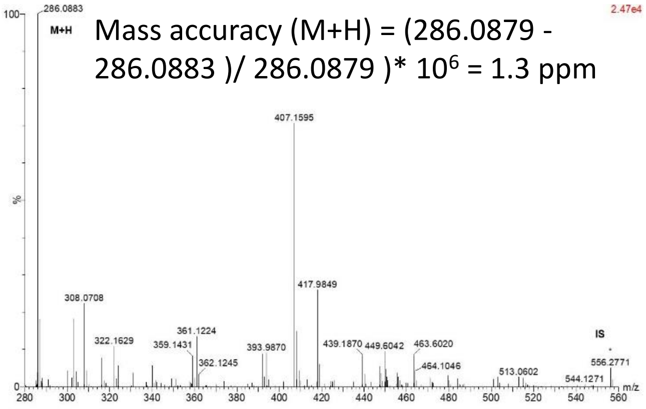

Mass accuracy (M+H) = (286.0879 - 286.0883)/ 286.0879)* 10^{6} = 1.3 ppm

Analysis Report

^{1}H NMR

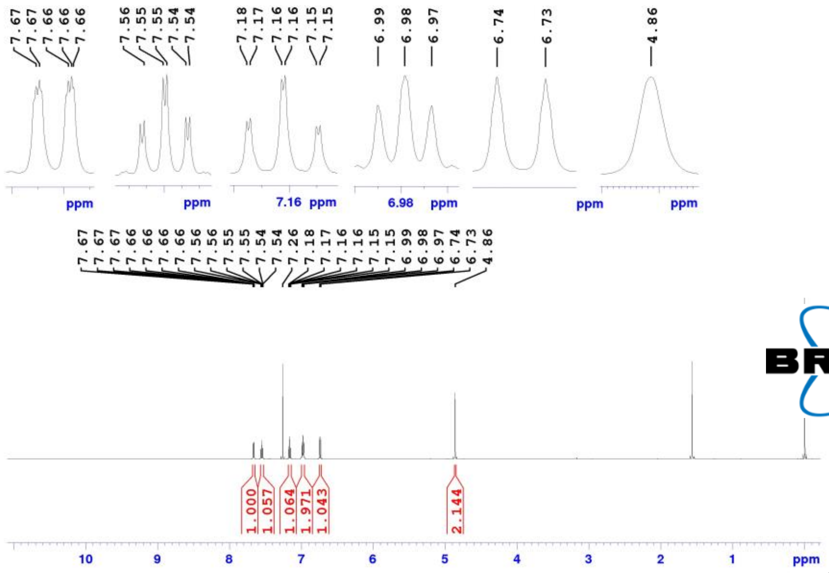

^{13}C APT NMR

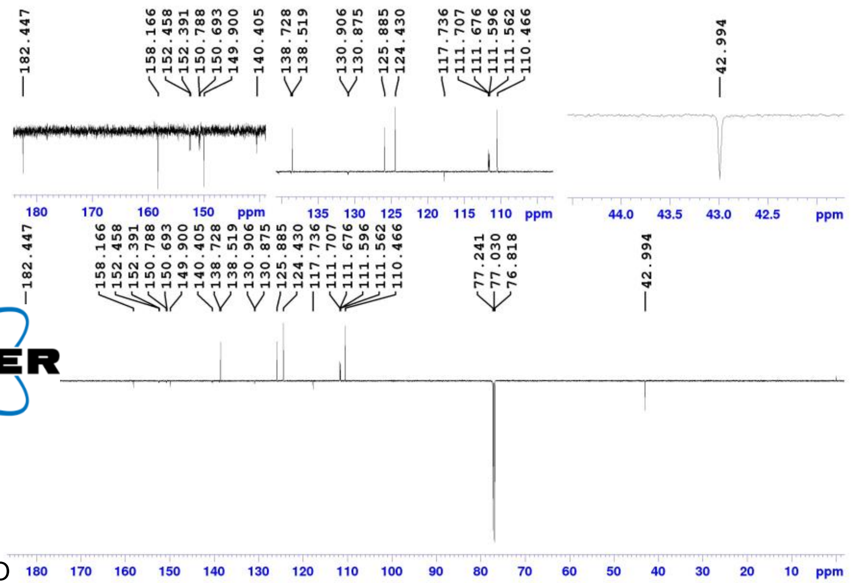

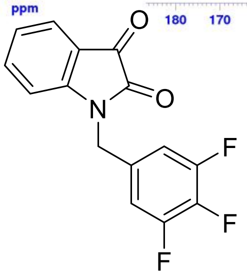

8i

HPLC purity
(2 wavelength)

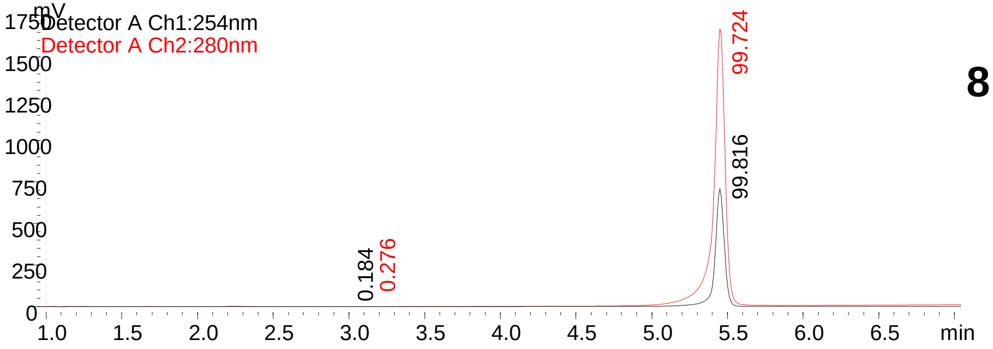

HRMS

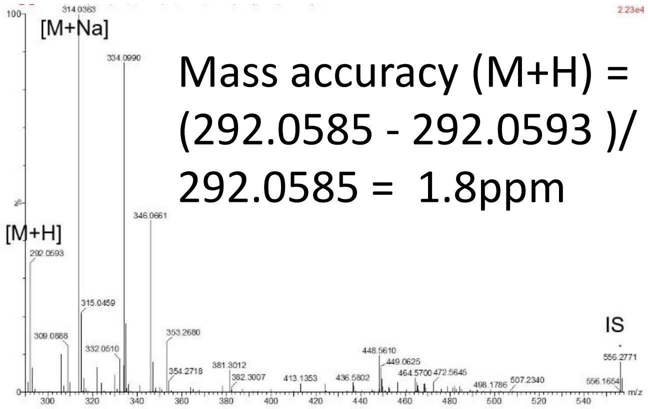

Mass accuracy (M+H) =
(292.0585 - 292.0593)/
292.0585 = 1.8ppm

Analysis Report

^{1}H NMR

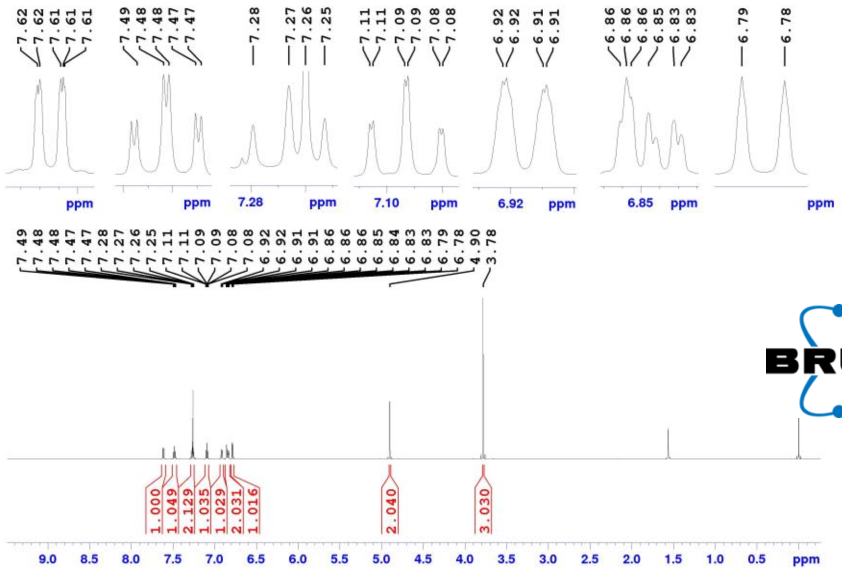

^{13}C APT NMR

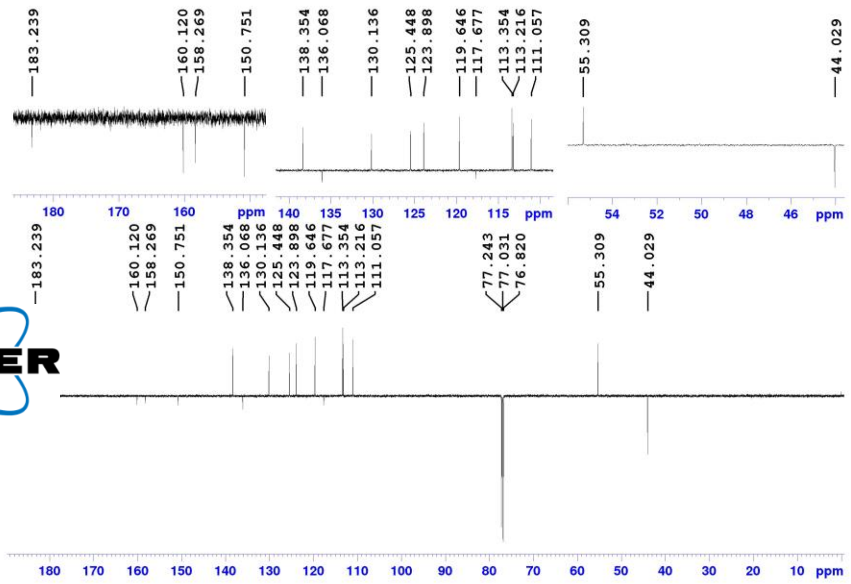

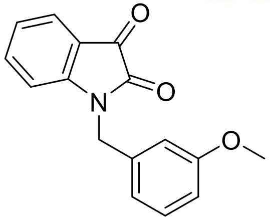

8j

HPLC purity (2 wavelength)

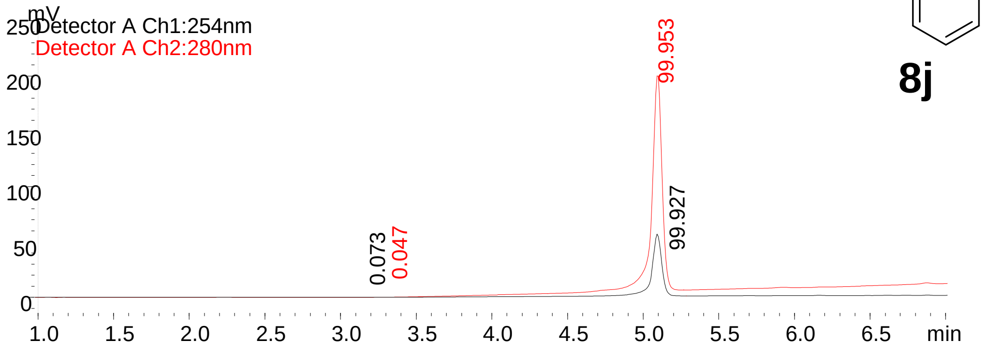

HRMS

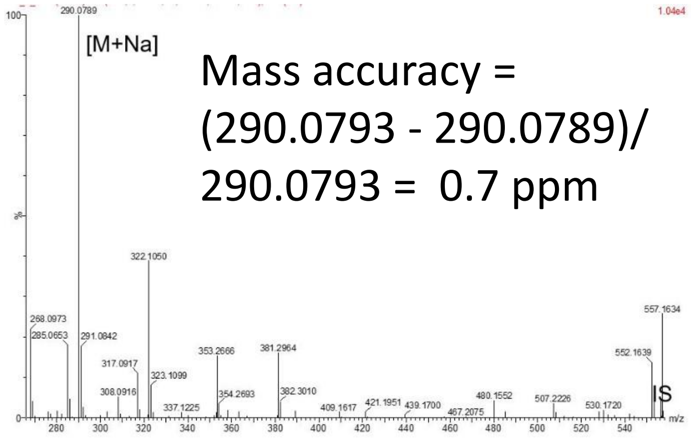

Mass accuracy = (290.0793 - 290.0789)/ 290.0793 = 0.7 ppm

Analysis Report

^{1}H NMR

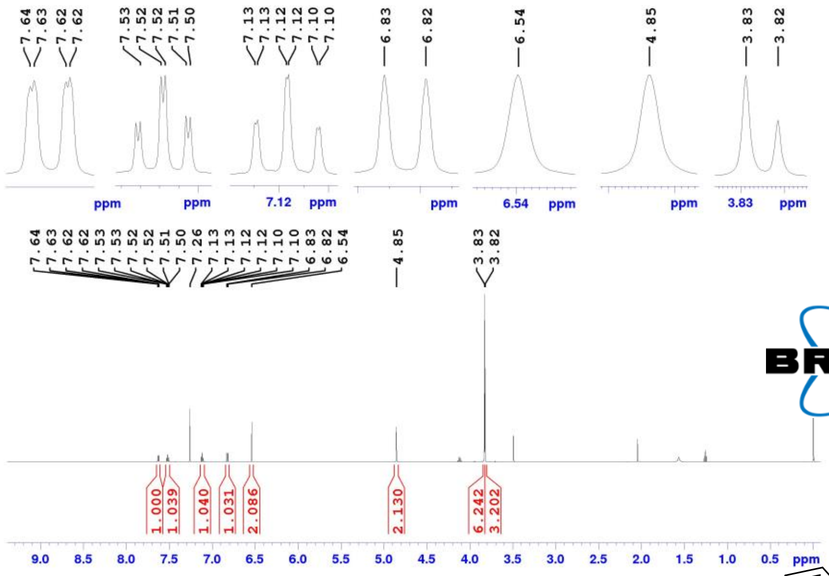

^{13}C APT NMR

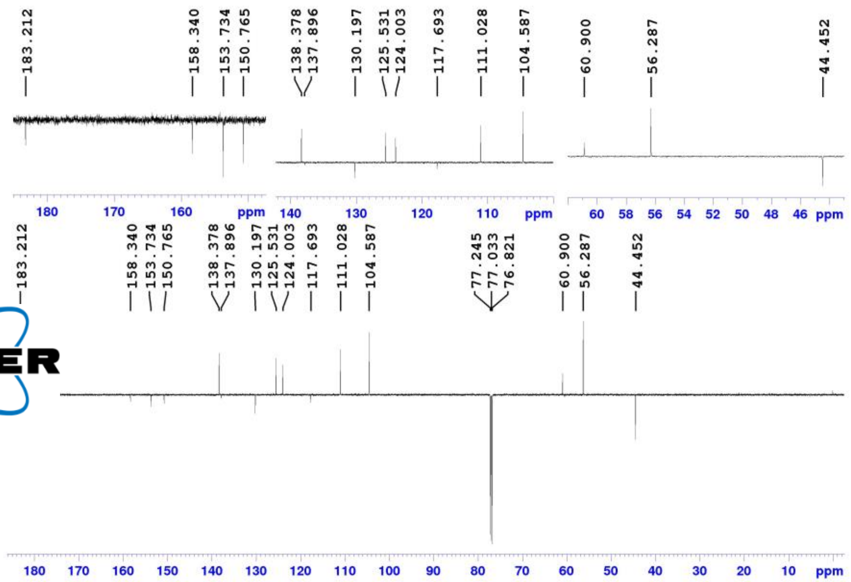

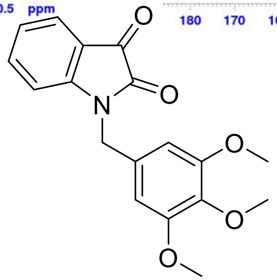

8k

HPLC purity
(2 wavelength)

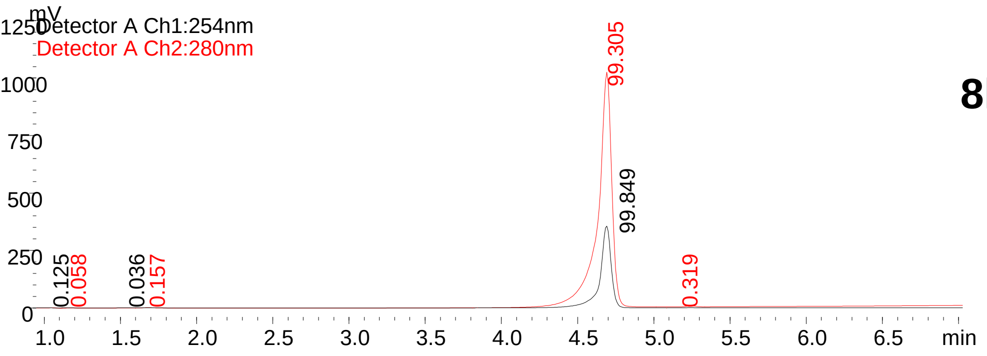

HRMS

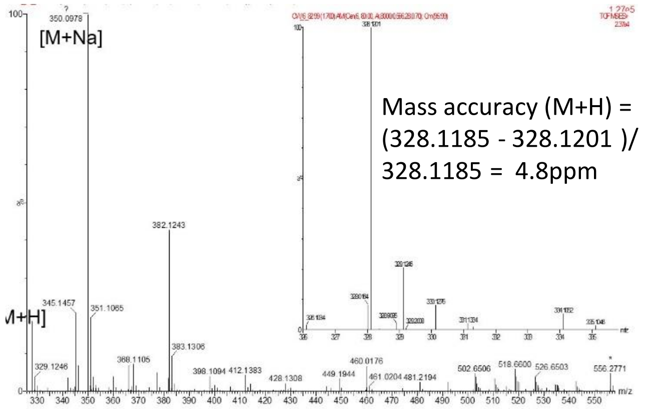

Analysis Report

^{1}H NMR

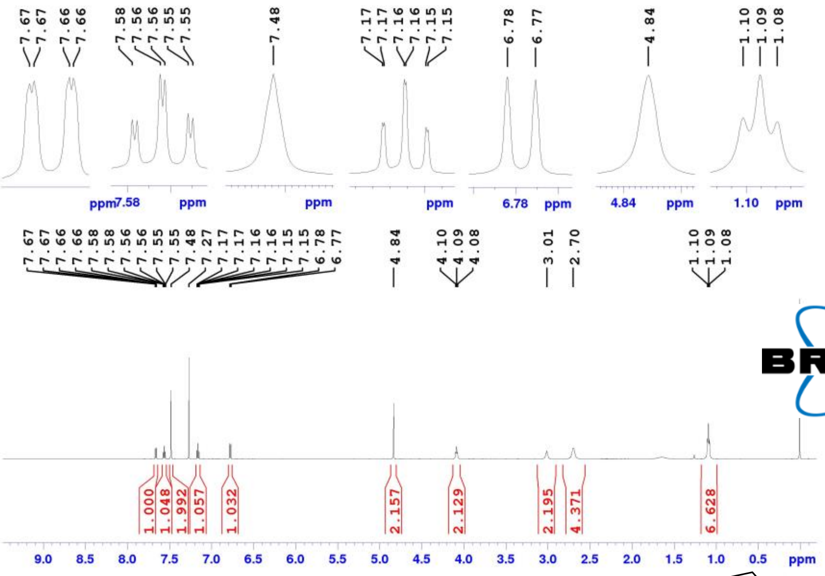

^{13}C APT NMR

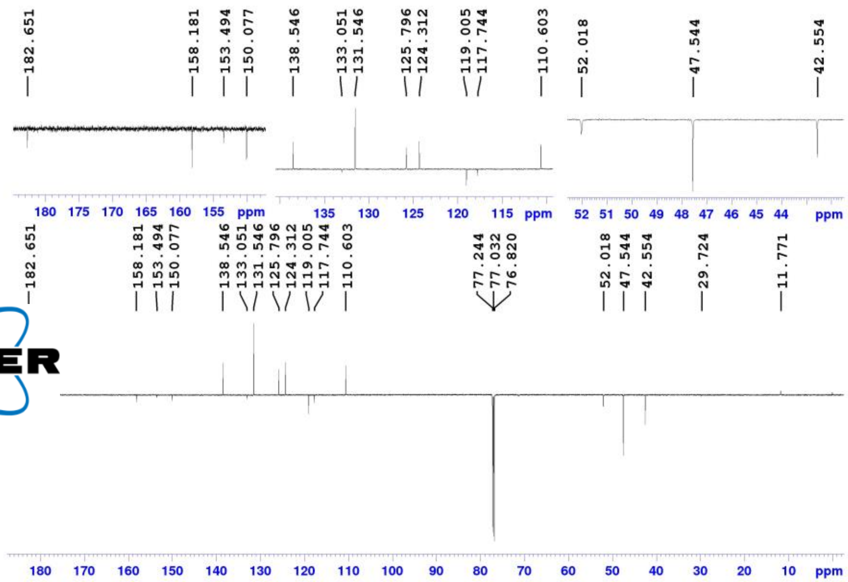

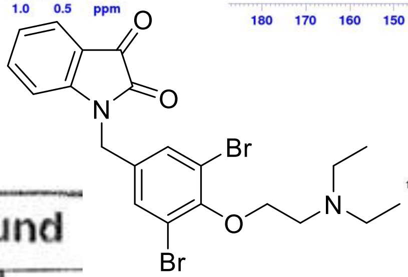

8l

Elemental Analysis purity

Element	Theory	Found
C	49.44	49.43
H	4.35	4.32
Br	31.32	31.09
N	5.49	5.49

HRMS

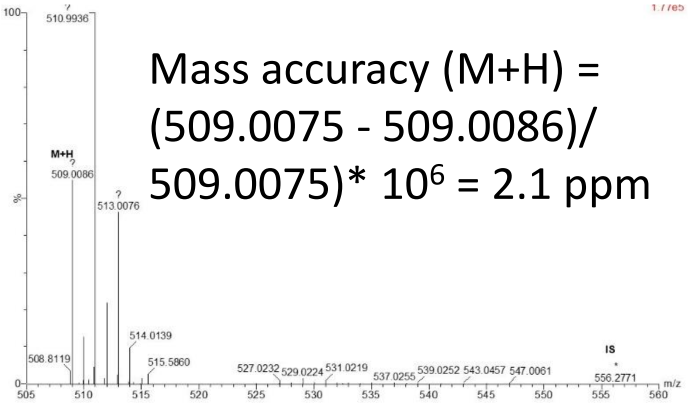

Mass accuracy (M+H) = (509.0075 - 509.0086)/ 509.0075)* 10^{6} = 2.1 ppm

Analysis Report

^{1}H NMR

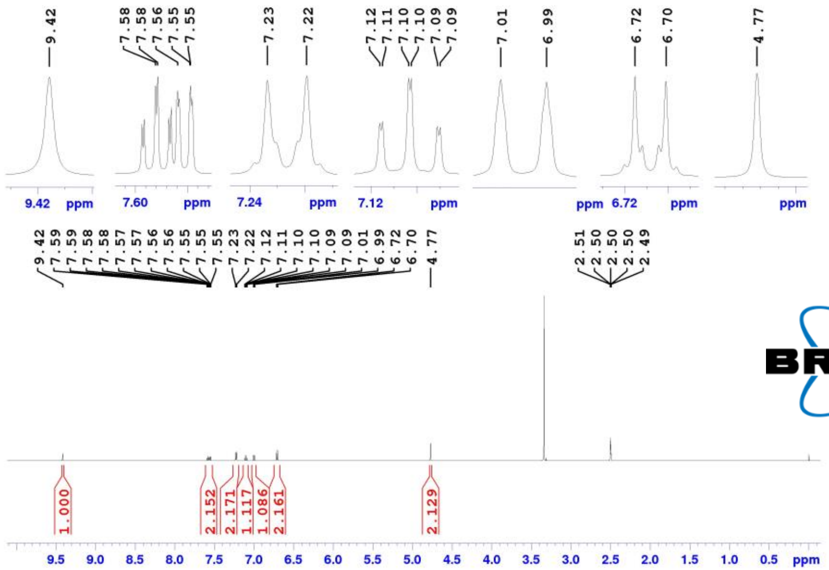

^{13}C APT NMR

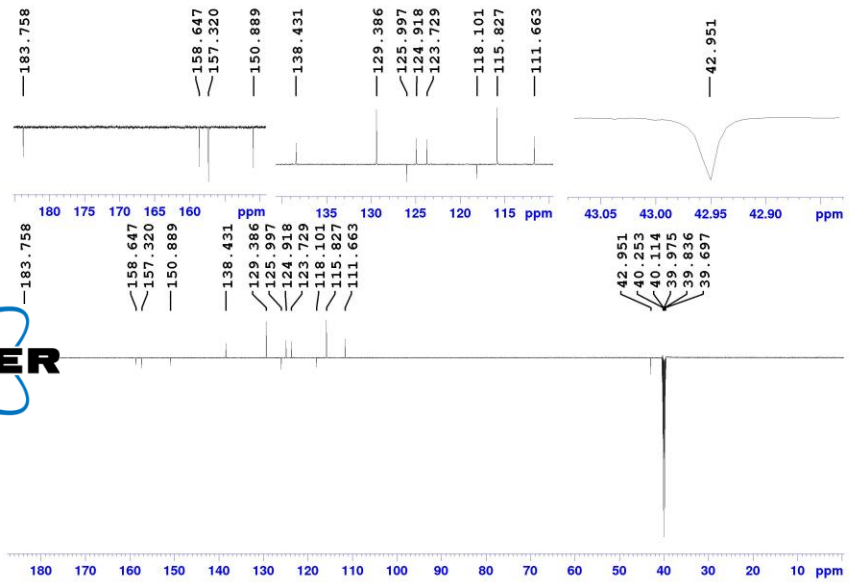

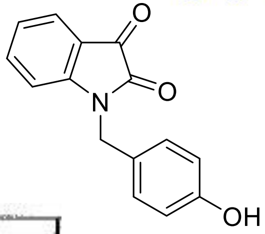

10

Elemental Analysis purity

Element	Theory	Found	
C	71.14	70.65	70.64
H	4.38	4.36	4.22
N	5.53	5.54	5.56

HRMS

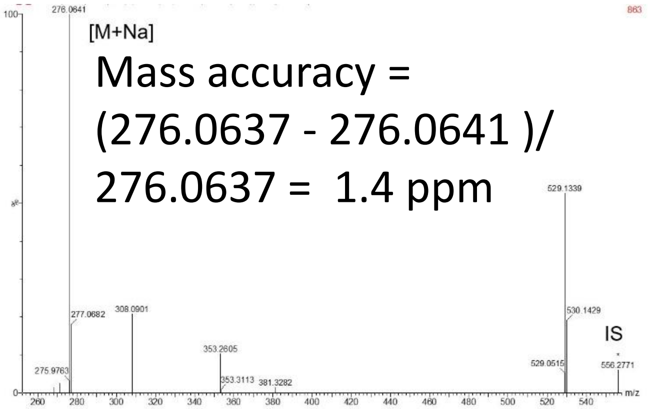

Supplement: Supplementary file 1 — Supplementary Figures. [file 41598_2024_65445_MOESM1_ESM.pdf]
